# The Ethanol‐Insoluble Low‐Molecular‐Weight Fraction LEP‐4 Derived From *Lentinula edodes* Mycelia Extract Attenuates Oxaliplatin‐Evoked Allodynia in Mice

**DOI:** 10.1155/adpp/4435116

**Published:** 2026-08-17

**Authors:** Masanobu Tsubaki, Taira Matsuo, Rie Komori, Tomoya Takeda, Noriaki Nagai, Toshio Morikawa, Shozo Nishida

**Affiliations:** ^1^ Laboratory of Pharmacotherapy, Faculty of Pharmaceutical Sciences at Kagawa Campus, Tokushima Bunri University, Sanuki 760-8542, Kagawa, Japan, bunri-u.ac.jp; ^2^ Faculty of Pharmacy, Osaka Ohtani University, Tondabayashishi 584-8540, Osaka, Japan, osaka-ohtani.ac.jp; ^3^ Department of Pharmacy, Kindai University, Higashiosaka 577-8502, Osaka, Japan, kindai.ac.jp; ^4^ Pharmaceutical Research and Technology Institute, Kindai University, Higashiosaka 577-8502, Osaka, Japan, kindai.ac.jp

**Keywords:** allodynia, ethanol-insoluble fraction, *Lentinula edodes* mycelia, neuropathy, oxaliplatin

## Abstract

Chemotherapy‐induced peripheral neuropathy (CIPN) is an adverse event caused by drugs such as bortezomib, vincristine, paclitaxel, and oxaliplatin (L‐OHP). This condition can lead to the discontinuation of anticancer drug administration and a diminished quality of life, posing a significant clinical problem. However, the only currently recognized effective treatment is duloxetine, and new therapeutic options are urgently required. *Lentinula edodes* mycelia extract (LEM) has been reported to improve CIPN in mouse models and alleviate numbness in patients with persistent neuropathy following L‐OHP treatment. However, the active ingredients responsible for the attenuation of neuropathy remain unknown. We extracted the fractions from LEM, and an L‐OHP‐evoked allodynia mouse model was used to investigate which fraction contained the active ingredient. LEM was separated into an ethanol‐soluble fraction (LES), an ethanol‐insoluble fraction (LEP), and LEM(−), a hot water extract from the LEM‐free medium. LEP abolished L‐OHP‐evoked allodynia, whereas LES and LEM(−) did not. In addition, fractionation of LEP based on molecular weight sieving yielded fractions LEP‐1 (molecular weight ≥ 100,000) and LEP‐2 (molecular weight < 100,000), the latter of which was found to inhibit L‐OHP‐evoked allodynia. Similarly, fractionation of LEP‐2 gave rise to LEP‐3 (molecular weight 3500–50,000) and LEP‐4 (molecular weight ≤ 3500), the former of which had no inhibitory effects on L‐OHP‐evoked allodynia, whereas an inhibitory effect was observed in response to the administration of LEP‐4. In addition, LEP‐4 was found to abrogate the expression of NR2A and Cav3.2 mRNAs in the dorsal root ganglion (DRG), and inhibited the activation of extracellular regulated‐protein kinase 1/2 (ERK1/2) in both the spinal cord and DRG. Moreover, oral administration of 0.25 g/kg LEP‐4 ameliorated allodynia, which had developed following L‐OHP administration. These findings indicate that the active component preventing CIPN in LEM resides in LEP‐4. This compound has prophylactic and therapeutic potential for L‐OHP‐evoked allodynia by abrogating NR2A and Cav3.2 mRNA expression and activating ERK1/2.

## 1. Introduction

Chemotherapy‐induced peripheral neuropathy (CIPN) is an adverse event induced by proteasome inhibitors such as bortezomib (BOR), vinca alkaloids such as vincristine (VCR), taxanes such as paclitaxel (PTX), and platinum‐based agents such as oxaliplatin (L‐OHP). It is an adverse event that often necessitates chemotherapy discontinuation or dose reduction [1, 2]. In addition, peripheral neuropathy was observed in 25%–40% of patients treated with L‐OHP at 2 years posttreatment, 41% of patients treated with PTX at 3 years posttreatment, and 13% of patients treated with VCR at 15 years posttreatment. This is associated with numbness and pain, causing a decline in the quality of life (QOL) [3–6]. Although duloxetine has received moderate recommendations by the American Society of Clinical Oncology as an effective drug for CIPN, its efficacy is limited [7]. Additionally, clinical trials on CIPN have been conducted using calmangafodipir, an iron chelator that mimics the activity of manganese superoxide dismutase, and the antioxidant N‐acetylcysteine; however, its efficacy has not been demonstrated [8, 9]. Therefore, it is necessary to develop new therapeutics for the treatment of CIPN.

*Lentinula edodes* mycelia extract (LEM) is a powder obtained by drying a solution extracted from the mycelia of *L. edodes* using a hot‐water extraction process [10, 11]. Oral administration of LEM has been demonstrated to improve the QOL of patients with breast and gastrointestinal cancers undergoing chemotherapy [12–14]. Furthermore, LEM exerts a protective effect against CCl_4_‐elicited liver dysfunction by suppressing the increase in hydroxyproline concentration in the mouse liver and the elevation of aspartate aminotransferase and alanine aminotransferase activities in plasma [15]. In addition, ethanol extracts of LEM have been demonstrated to inhibit tert‐butyl hydroperoxide‐induced cell death in HT22 neuronal cells [16]. We have previously demonstrated that oral administration of LEM exerts prophylactic and therapeutic effects against BOR‐, VCR‐, PTX‐, and L‐OHP‐evoked neuropathy [17]. Moreover, LEM has been shown to improve numbness in patients with colorectal cancer with peripheral neuropathy persisting for more than 3 months after L‐OHP therapy [18]. However, the active ingredient responsible for the CIPN‐ameliorating effect of LEM remains unknown. In this study, LEM fractions were extracted, and the fractions that abrogated L‐OHP‐evoked allodynia were investigated. On the basis of our use of dialysis membranes, we have identified LEP‐4, a fraction with a molecular weight of 3500–5,000, as the active component of LEM with effects against L‐OHP‐evoked neuropathy. Similar to LEM, we have demonstrated that LEP‐4 has preventive and therapeutic effects against L‐OHP‐evoked neuropathy, and established that its mechanism of action involves abrogation of the expression of Cav3.2 and NR2A mRNAs, and activation of ERK1/2 in the dorsal root ganglia (DRG) and spinal cord of mice. Accordingly, either LEM or LEP‐4 may be effective treatments for CIPN, including L‐OHP‐evoked neuropathy.

## 2. Materials and Methods

### 2.1. Drugs

LEM was supplied by Kobayashi Pharmaceutical Co., Ltd. (Osaka, Japan). As mentioned previously, LEM dried powder was obtained by cultivating shiitake fungus on a medium composed of bagasse and rice bran, extracting it with boiling water, and grinding it immediately before germination when the mycelia have spread throughout the medium [10, 11]. LEM(−) was prepared by extracting the LEM medium with boiling water and grinding the resulting material. LEM was prepared using a previously reported method and dissolved in phosphate‐buffered saline (PBS) [11, 17].

L‐OHP (FUJIFILM Wako, Osaka, Japan) was dissolved in 5% glucose. Duloxetine (FUJIFILM Wako, Osaka, Japan) was dissolved in dimethyl sulfoxide (DMSO) and then diluted to 0.5% DMSO in phosphate‐buffered saline.

### 2.2. Procedure for Preparing LEM Fractions

LEM (100.0 g) was dissolved in distilled water (1 L), and the solution was added to Celite545RVS (100 g, Nacalai Tesque Inc., Kyoto, Japan) and stirred at room temperature for 15 min. The mixture was filtered through a glass fiber filter GA200 (Advantec Co., Ltd., Tokyo, Japan) by suction filtration to obtain the filtrate. Fifty grams of Celite was added to the obtained filtrate, and after stirring for 15 min, the mixture was filtered as described above. Four times the volume of ethanol (99.5%, v/v, 4 L) was added to the resulting filtrate while stirring, following which the mixture was stored overnight at 4°C and centrifuged at 3000 rpm for 10 min to generate a precipitate (LEP, 33.45 g, 33.5%) and a supernatant (LES, 42.85 g, 42.9%). An aliquot of LEP (30.00 g) was dissolved in distilled water (300 mL), following which the solution was dialyzed using a dialysis membrane (SpectraPor Biotech CE Tubing molecular weight cutoff [MWCO] 100,000, Funakoshi Co., Ltd., Tokyo, Japan) to produce fractions of internal solution (LEP‐1, 4.29 g, 4.8%) and external solution (LEP‐2, 25.85 g, 28.9%). An aliquot of the external solution (20.00 g) was dialyzed using a dialysis membrane (MWCO 50,000) to generate fractions of internal solution (0.40 g, 0.6%) and external solution, which was further dialyzed (MWCO 3500–5000) to produce fractions of internal solution (LEP‐3, 8.07 g, 11.7%) and external solution (LEP‐4, 11.83 g, 17.1%) (Supporting Figure 1).

### 2.3. L‐OHP‐Evoked Allodynia Models

The Tokushima Bunri University Animal Care and Use Committee approved all animal experiments with approval numbers KP24‐51‐9 (May 20, 2024) and KP25‐51‐8 (March 17, 2025). Five‐week‐old male BALB/c mice were procured from SHIMIZU Laboratory Supplies Co., Ltd (Kyoto, Japan).

To evaluate the prophylactic effects of LEM, LES, LEP, LEM(−), LEP‐1, LEP‐2, LEP‐3, LEP‐4, and duloxetine oral treatments on allodynia, mice were administered 6 mg/kg L‐OHP (intraperitoneal injection [i.p.] on Days 0, 7, and 14), or vehicle (5% glucose, i.p.) (*n* = 10 for each group). LEM, LES, LEP, LEM(−), LEP‐1, LEP‐2, LEP‐3, LEP‐4, and duloxetine were administered to the mice 12 h after L‐OHP administration (Day 0). L‐OHP was administered at 08:00, whereas LEM, LES, LEP, LEM(−), LEP‐1, LEP‐2, LEP‐3, LEP‐4, and duloxetine were administered at 20:00. Behavioral assessments were performed on Days 0, 3, 6, 9, 12, and 15. LES, LEP, LEM(−), LEP‐1, LEP‐2, LEP‐3, LEP‐4, and duloxetine (1 g/kg, 1 g/kg, 1 g/kg, 0.5 g/kg, 0.5 g/kg, 0.25 g/kg, 0.25 g/kg, or 30 mg/kg orally [p.o.]) or PBS (vehicle, p.o.) were administered daily from Days 0–15 (*n* = 10 per group) after the behavioral evaluations.

To examine the therapeutic effect of oral administration of LEP‐4 on allodynia, mice were administered 6 mg/kg L‐OHP or vehicle (5% glucose) (*n* = 10 for each group) on Days 0, 7, 14, and 21. LEP‐4 was administered to the mice 12 h after the administration of L‐OHP (Day 6). l‐OHP was administered at 08:00, whereas LEP‐4 was administered at 20:00. Behavioral assessments were performed on Days 0, 3, 6, 9, 12, 15, 18, 21, and 24. Following behavioral assessments, mice (*n* = 10 per group) received daily (Days 6–24) LEP‐4 (0.25 g/kg, orally) or PBS (vehicle, orally).

All behavioral tests were conducted in blinded conditions. In addition, the sample size for the mice was determined by referring to our previous studies [17, 19–23].

### 2.4. Behavioral Assessments

Behavioral assessments were performed as described previously [17, 19–23]. The sensitivity to cold of each mouse was assessed by employing a hot/cold plate analgesiometer (Ugo Basile S.R.L., Milan, Italy). The mice were placed in the middle of a plate maintained at 10°C, and any pain‐related behaviors, such as licking or lifting their hindlimbs, were observed within 30 s (with a maximum observation period of 30 s).

Mechanical sensitivity was assessed using von Frey filaments (0.16, 0.4, and 1.4 g, Ugo Basile S.R.L.). The sensitivity of each filament was determined by five stimulations at 5‐s intervals and subsequently scored (0, no reaction; 1, pulling out the paw; 2, the stimulated paw immediately flinches). Five tests were performed on both hindlimbs of each mouse, and the avoidance response scores were measured as the averages and noted as the mean ± standard error of the mean (SEM).

### 2.5. Quantitative Reverse‐Transcription Polymerase Chain Reaction (qRT‐PCR)

The mRNA expression levels extracted from the DRG were determined by employing qRT‐PCR as previously described [17]. PCR conditions for amplification of NR2A, Cav3.2, and glyceraldehyde 3‐phosphate dehydrogenase (GAPDH) genes were 94°C for 2 min, followed by 40 cycles of 94°C for 0.5 min, 50°C for 0.5 min, and 72°C for 0.5 min. The following primer sequences were employed: NR2A: 5′‐ACC TCG CTC TGC TCC AGT TT‐3′ (forward) and 5′‐GTT GTG GCA GAT GCC CGT AA‐3′ (reverse), Cav3.2: 5′‐GTC TCG TTC CTG TCC ATC AC‐3′ (forward) and 5′‐TGC ATG GGC CAG TAC TCC TT‐3′ (reverse), and GAPDH: 5′‐ACT TTG TCA AGC TCA TTT‐3′ (forward) and 5′‐TGC AGC GAA CTT TAT TG‐3′ (reverse). To verify the efficiency of the primers during the reaction, we undertook a preliminary assessment and plotted a standard curve. Results were presented as target mRNA levels quantified against GAPDH levels.

### 2.6. Western Blotting

The protein expression levels extracted from the DRG and spinal cord were determined by western blotting as previously described [17]. Briefly, a protein sample (20 μg) was separated on a 10% SDS‐polyacrylamide gel and then blotted onto polyvinylidene fluoride membranes (#10600023; Cytiva, Marlborough, MA, USA). The membranes were initially blocked with 5% skimmed milk and then incubated with the following primary antibodies: antiextracellular regulated‐protein kinase 1/2 (ERK1/2) (rabbit 1:1000: #9102; Cell Signaling Technology, Beverly, MA, USA), anti–phospho‐ERK1/2 (rabbit 1:1000: #9101; Cell Signaling Technology), and anti–β‐actin (mouse 1:3000: sc‐47778; Santa Cruz Biotechnology, CA, USA). Following incubation with the corresponding horseradish peroxidase‐linked antirabbit and antimouse secondary antibodies (goat 1:3000: #7074 and horse 1:5000: #7076; Cell Signaling Technology), the membranes were visualized using Luminata Forte (#WBLUF0500; Merck Millipore, Nottingham, UK). Detection was performed using a ChemiDoc Touch MP Imaging System (Bio‐Rad, Hercules, CA, USA). Detection of ERK1/2 (following detection of phospho‐ERK1/2) and *β*‐actin (following detection of ERK1/2) was performed after stripping the antibodies from the membrane using a stripping buffer (#21059; Thermo Fisher Scientific, Waltham, MA, USA). *β*‐Actin was used as a loading control. The resolved signals were analyzed using Image Lab software Version 6.1 (Bio‐Rad) and normalized against the ERK1/2 protein.

### 2.7. Statistics

The mean ± SEM or standard deviation (SD) is displayed for at least three independent experiments. Tukey’s test was employed for multiple comparisons following analysis of variance, whereas Dunnett’s test was employed to compare the control group to the drug‐treated group. Normality of the data was assessed with the Shapiro–Wilk test; if not normally distributed, the Kruskal–Wallis test was employed, followed by the Scheffe test. Statistical significance was set at *p* < 0.05.

## 3. Results

### 3.1. LEP Attenuated L‐OHP‐Evoked Allodynia

First, to identify the active ingredient in LEM, it was separated into ethanol‐soluble fractions (LES) and ethanol‐insoluble fractions (LEP), and a medium alone (LEM(−)) was prepared. To evaluate the prophylactic effects of LES, LEP, and LEM(−) on L‐OHP‐evoked cold and mechanical allodynia, 1 g/kg LES, LEP, or LEM(−) was administered daily to mice treated with L‐OHP at a 1‐week interval (Days 0, 7, and 14). L‐OHP evoked a substantial decrease in the threshold for withdrawal at 10°C (Figure 1a and Supporting Table 1). Orally administered 2 g/kg LEM and 1 g/kg LEP markedly abrogated L‐OHP‐evoked cold allodynia; however, treatment with 1 g/kg LES and LEM(−) was ineffective (Figure 1a and Supporting Table 1). Mice administered L‐OHP, LEM, LES, LEP, or LEM(−) did not show a decrease in body weight (Figure 1b and Supporting Table 2). Although treatment with 1 g/kg LES and LEM(−) did not inhibit L‐OHP‐evoked mechanical allodynia, the administration of 2 g/kg LEM and 1 g/kg LEP abrogated this drug‐evoked mechanical allodynia (0.16, 0.4, and 1.4 g, respectively) (Figure 1c–e and Supporting Tables 3–5). These results indicated that LEP, the ethanol‐insoluble fraction of LEM, attenuated L‐OHP‐evoked cold and mechanical allodynia.

FIGURE 1LEP attenuated L‐OHP‐evoked allodynia. (a) The hindlimb withdrawal latency after cold stimulation (10°C) exposure is shown as the mean ± SEM (*n* = 10 per group). ^∗^ *p* < 0.01 versus vehicles (Shapiro–Wilk test and Kruskal–Wallis test followed by the Scheffe test). (b) The change in mouse body weight after L‐OHP, LEM, LES, LEP, or LEM(−) treatment is shown as the mean ± SEM (*n* = 10 per group) (Shapiro–Wilk test and one‐way analysis of variance [ANOVA] with Tukey’s test). (c–e) The number of times the paw was lifted in response to five mechanical stimulations utilizing von Frey filaments with bending forces of (a) 0.16 g, (b) 0.4 g, and (c) 1.4 g was recorded. The aversion response scores were determined by measuring the elevation of the hind paw. Data are displayed as means ± SEM (*n* = 10 per group). ^∗^ *p* < 0.01 versus vehicles (Shapiro–Wilk test and ANOVA with Tukey’s test).

**Figure:**
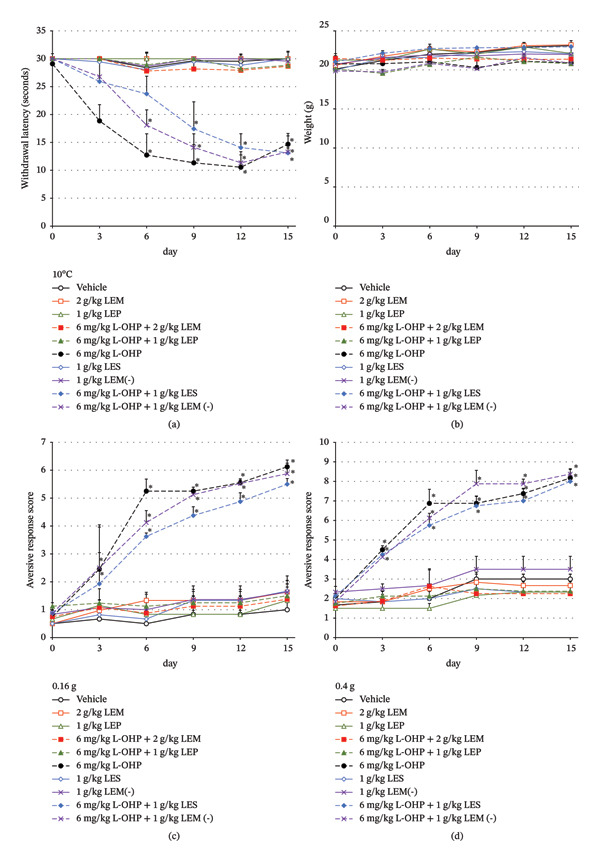

**Figure:**
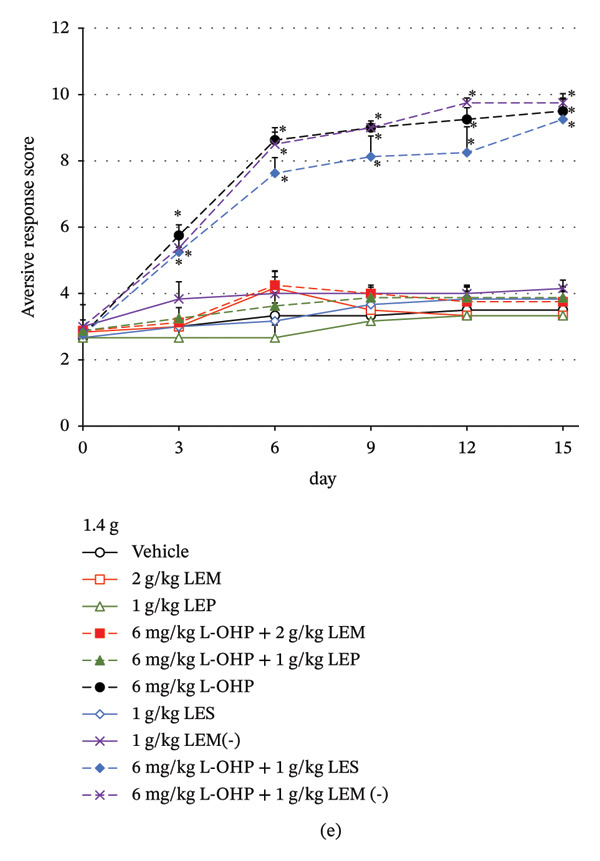

### 3.2. LEP‐2 Attenuated L‐OHP‐Evoked Allodynia

Next, LEP was separated using a dialysis membrane with a MWCO of 100,000 into fractions above this size (LEP‐1) and below (LEP‐2). We investigated whether these fractions exert preventive effects against L‐OHP‐evoked cold and mechanical allodynia. Orally administered 2 g/kg LEM, 1 g/kg LEP, and 0.5 g/kg LEP‐2 markedly abrogated L‐OHP‐evoked cold or mechanical allodynia; however, treatment with 0.5 g/kg LEP‐1 was ineffective (Figure 2a,c,d,e and Supporting Tables 6–9). Mice administered L‐OHP, LEM, LEP, LEP‐1, or LEP‐2 showed no decrease in body weight (Figure 2b and Supporting Table 10). These results demonstrated that LEP‐2, a fraction with a molecular weight of 100,000 or less from the ethanol‐insoluble fraction of LEM, attenuated cold and mechanical allodynia induced by L‐OHP.

FIGURE 2LEP‐2 attenuated L‐OHP‐evoked allodynia. (a) The hindlimb withdrawal latency after cold stimulation (10°C) exposure is shown as the mean ± SEM (*n* = 10 per group). ^∗^ *p* < 0.01 versus vehicles (Shapiro–Wilk test and Kruskal–Wallis test followed by the Scheffe test). (b) The change in mouse body weight after L‐OHP, LEM, LEP, LEP‐1, or LEP‐2 treatment is shown as the mean ± SEM (*n* = 10 per group) (Shapiro–Wilk test and one‐way analysis of variance [ANOVA] with Tukey’s test). (c–e) The number of times the paw was lifted in response to five mechanical stimulations utilizing von Frey filaments with bending forces of (a) 0.16 g, (b) 0.4 g, and (c) 1.4 g was recorded. The aversion response scores were determined by measuring the elevation of the hind paw. Data are displayed as means ± SEM (*n* = 10 per group). ^∗^ *p* < 0.01 versus vehicles (Shapiro–Wilk test and ANOVA with Tukey’s test).

**Figure:**
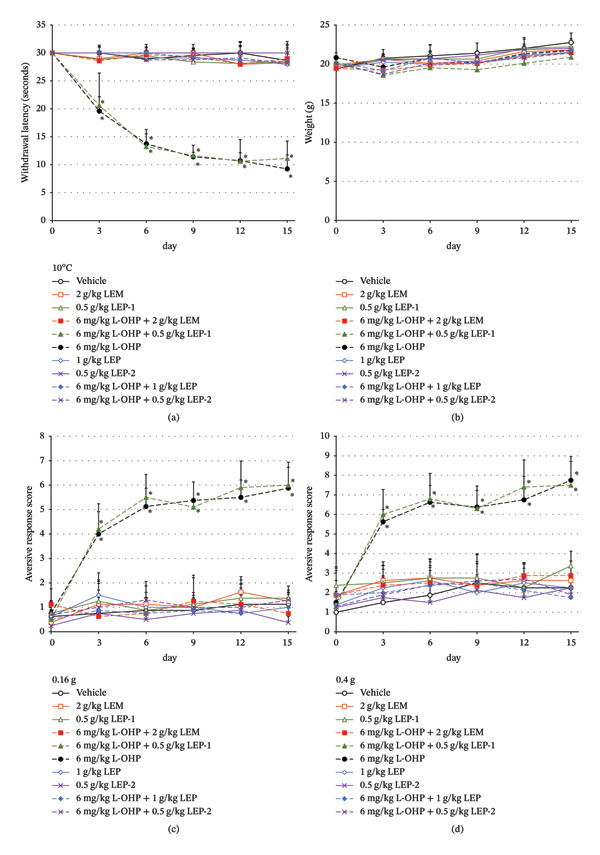

**Figure:**
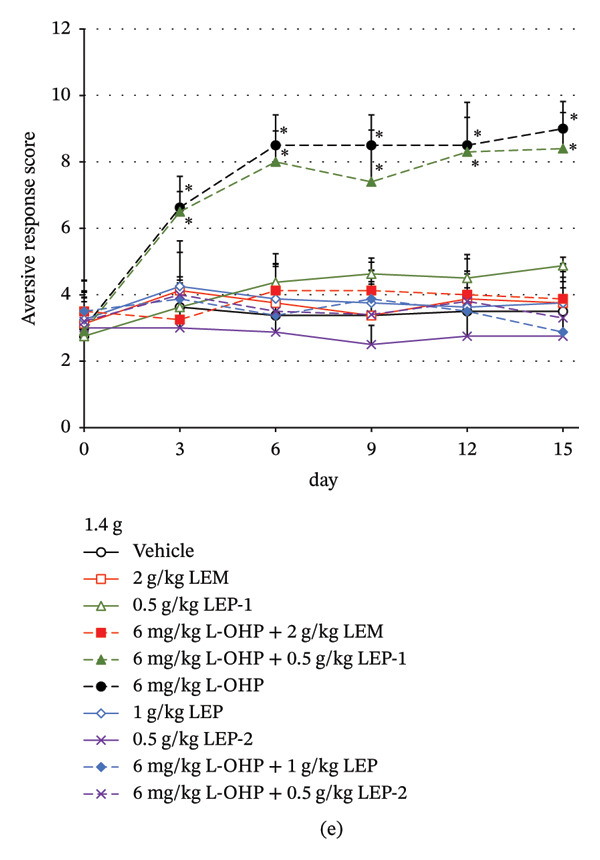

### 3.3. LEP‐4 Attenuated L‐OHP‐Evoked Allodynia

The dialysate obtained by separating LEP‐2 using a dialysis membrane with a MWCO of 50,000 was further separated using a dialysis membrane with a MWCO of 3500–5000. This resulted in fractions with molecular weights of 3500–50,000 (LEP‐3) and 3500 or less (LEP‐4). Orally administered 2 g/kg LEM, 0.5 g/kg LEP‐2, 0.25 g/kg LEP‐4, and 30 mg/kg duloxetine markedly abrogated L‐OHP‐evoked cold or mechanical allodynia; however, treatment with 0.25 g/kg LEP‐3 was ineffective (Figure 3a,c,d,e and Supporting Tables 11–14). Mice treated with L‐OHP, LEM, LEP‐2, LEP‐3, LEP‐4, or duloxetine showed no decrease in body weight (Figure 3b and Supporting Table 15). These results demonstrate that LEP‐4, a fraction with a molecular weight of 3500 or less from the ethanol‐insoluble fraction of LEM, attenuated cold and mechanical allodynia induced by L‐OHP. Additionally, oral administration of 0.25 g/kg LEP‐4 demonstrated efficacy equivalent to that of 30 mg/kg duloxetine. The effects of LEM fractions on L‐OHP‐evoked neuropathy based on the present findings are summarized in Table 1.

FIGURE 3LEP‐4 attenuated L‐OHP‐evoked allodynia. (a) The hindlimb withdrawal latency after cold stimulation (10°C) exposure is shown as the mean ± SEM (*n* = 10 per group). ^∗^ *p* < 0.01 versus vehicles (Shapiro–Wilk test and Kruskal–Wallis test followed by the Scheffe test). (b) The change in mouse body weight after L‐OHP, LEM, LEP‐2, LEP‐3, LEP‐4, or duloxetine treatment is shown as the mean ± SEM (*n* = 10 per group) (Shapiro–Wilk test and one‐way analysis of variance [ANOVA] with Tukey’s test). (c–e) The number of times the paw was lifted in response to five mechanical stimulations utilizing von Frey filaments with bending forces of (a) 0.16 g, (b) 0.4 g, and (c) 1.4 g was recorded. The aversion response scores were determined by measuring the elevation of the hind paw. Data are displayed as means ± SEM (*n* = 10 per group). ^∗^ *p* < 0.01 versus vehicles (Shapiro–Wilk test and ANOVA with Tukey’s test).

**Figure:**
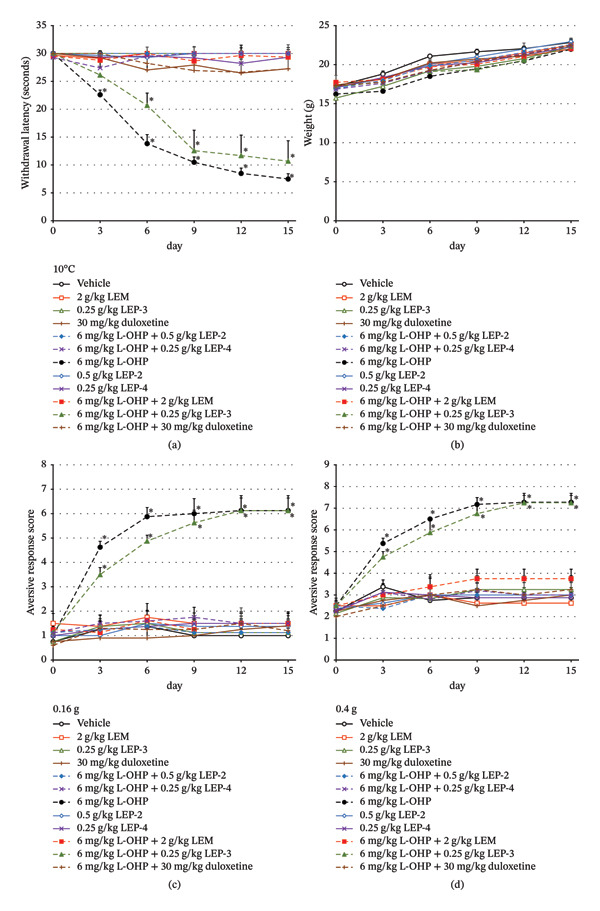

**Figure:**
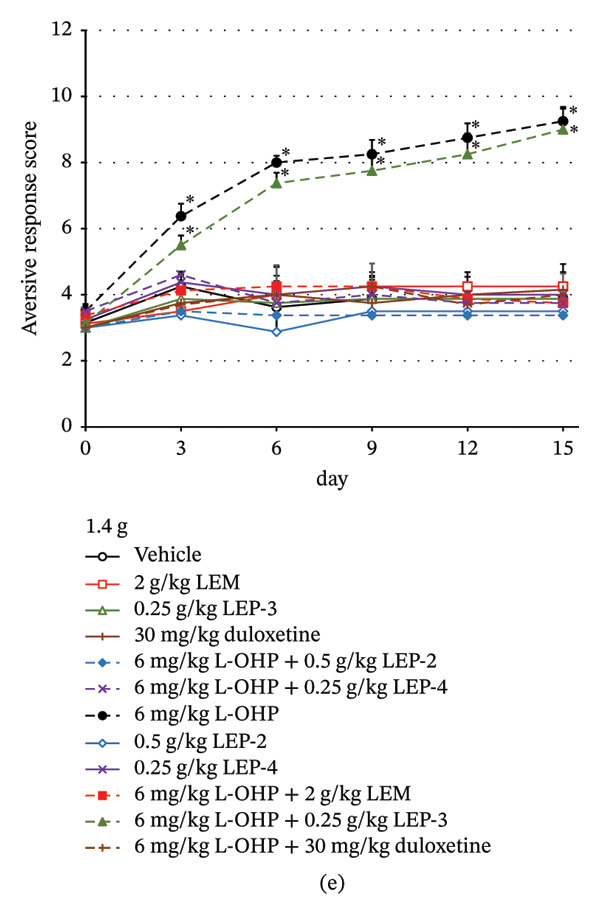

**TABLE 1:** A summary of the inhibitory effects of LEM fractions on L‐OHP‐evoked neuropathy.

LEM fractions	Dosage	Inhibitory effect on L‐OHP‐evoked neuropathy
LES (ethanol‐soluble fraction)	1 g/kg	Negative
LEP (ethanol‐insoluble fraction)	1 g/kg	Positive
LEM(−) (medium alone)	1 g/kg	Negative
LEP‐1 (LEP fraction with a MWCO of 100,000 or higher)	0.5 g/kg	Negative
LEP‐2 (LEP fraction with a MWCO of less than 100,000)	0.5 g/kg	Positive
LEP‐3 (LEP fraction with a MW of 3500–50,000)	0.25 g/kg	Negative
LEP‐4 (LEP fraction with a MW of 3500 or less)	0.25 g/kg	Positive

*Note:* MWCO, molecular weight cutoff.

### 3.4. LEP‐4 Abrogated L‐OHP‐Evoked Expression of NR2A and Cav3.2 MRNA in the DRG

Our previous results indicated that LEM inhibits L‐OHP‐elicited enhancement of NR2A and Cav3.2 mRNA expression in the DRG. Therefore, we evaluated whether LEP‐4 inhibits L‐OHP‐elicited enhancement of NR2A and Cav3.2 mRNA expression in the DRG. The administration of LEP‐4 abrogated L‐OHP‐evoked NR2A and Cav3.2 (Figure 4a,b) and Supporting Table 16). These results suggest that LEP‐4 abrogated L‐OHP‐evoked allodynia through a reduction in the expression of NR2A and Cav3.2 mRNA.

FIGURE 4LEP‐4 attenuated L‐OHP‐evoked enhancing expression of Cav3.2 and NR2A mRNA, and activation of ERK1/2. (a, b) (a) Cav3.2 and (b) NR2A mRNA levels were quantified using qRT‐PCR after treatment with L‐OHP and LEP‐4. (c–f) Western blotting was performed on (c) DRG and (e) spinal cord samples to evaluate the expression levels of phospho‐ERK1/2. (d, f) Phospho‐ERK1/2 expression was normalized to ERK1/2 expression. Results are indicative of three independent experiments and displayed as means ± S.D. (*n* = 4 per group). ^∗^ *p* < 0.01 versus vehicles (Shapiro–Wilk test and one‐way analysis of variance with Dunnett’s test).

**Figure:**
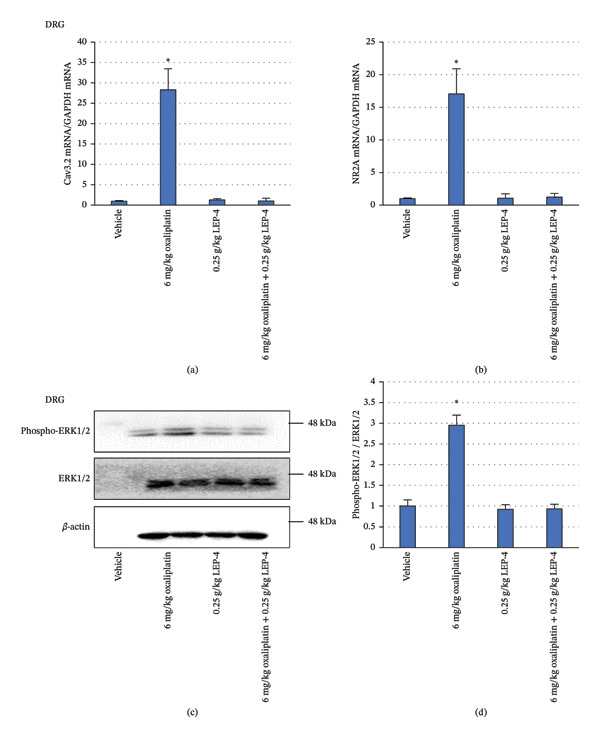

**Figure:**
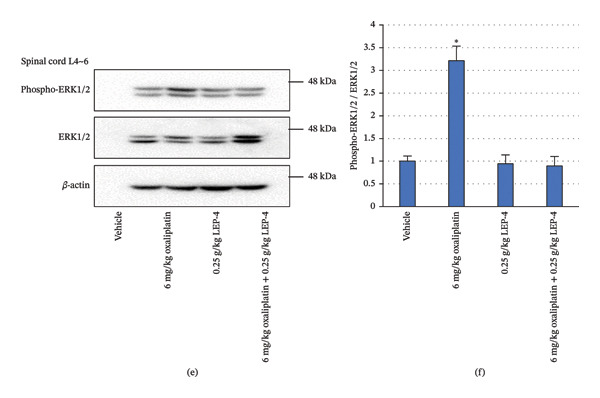

### 3.5. LEP‐4 Abrogated L‐OHP‐Evoked Activation of ERK1/2 in DRG and Spinal Cords

Our previous results indicate that LEM inhibits L‐OHP‐elicited activation of ERK1/2 in the DRG and spinal cord. Therefore, we evaluated whether LEP‐4 suppresses L‐OHP‐evoked ERK1/2 activation in the DRG and lumbar spinal segments L4–L6. The administration of LEP‐4 diminished L‐OHP‐evoked ERK1/2 activation to a degree comparable to that of the vehicle (Figure 4c–f and Supporting Table 17). These results suggest that LEP‐4 abrogated the expression of NR2A and Cav3.2 and NR2A mRNA by reducing the activation of ERK1/2.

### 3.6. LEP‐4 Improves L‐OHP‐Evoked Allodynia

To evaluate the therapeutic effects of LEP‐4 on L‐OHP‐evoked allodynia, mice that developed allodynia after L‐OHP treatment received 0.25 g/kg LEP‐4 once daily starting 6 days after L‐OHP treatment. The administration of LEP‐4 strikingly improved L‐OHP‐evoked allodynia (Figure 5a,c,d,e and Supporting Tables 18–21). Mice administered L‐OHP or LEP‐4 showed no decrease in body weight (Figure 5b and Supporting Table 22). These results suggest that LEP‐4 improved symptoms in mice that developed allodynia after L‐OHP treatment.

FIGURE 5LEP attenuated cold and mechanical allodynia that already existed following L‐OHP administration. (a) The hindlimb withdrawal latency after cold stimulation (10°C) exposure is shown as the mean ± SEM (*n* = 10 per group). ^∗^ *p* < 0.01 versus vehicles, ^#^ *p* < 0.01 versus 6 mg/kg oxaliplatin (Shapiro–Wilk test and Kruskal–Wallis test followed by the Scheffe test). (b) The change in mouse body weight after L‐OHP or LEP‐4 treatment is shown as the mean ± SEM (*n* = 10 per group) (Shapiro–Wilk test and one‐way analysis of variance [ANOVA] with Tukey’s test). (c–e) The number of times the paw was lifted in response to five mechanical stimulations utilizing von Frey filaments with bending forces of (a) 0.16 g, (b) 0.4 g, and (c) 1.4 g was recorded. The aversion response scores were determined by measuring the elevation of the hind paw. Data are displayed as means ± SEM (*n* = 10 per group). ^∗^ *p* < 0.01 versus vehicles, ^#^ *p* < 0.01 versus 6 mg/kg oxaliplatin (Shapiro–Wilk test and ANOVA with Tukey’s test).

**Figure:**
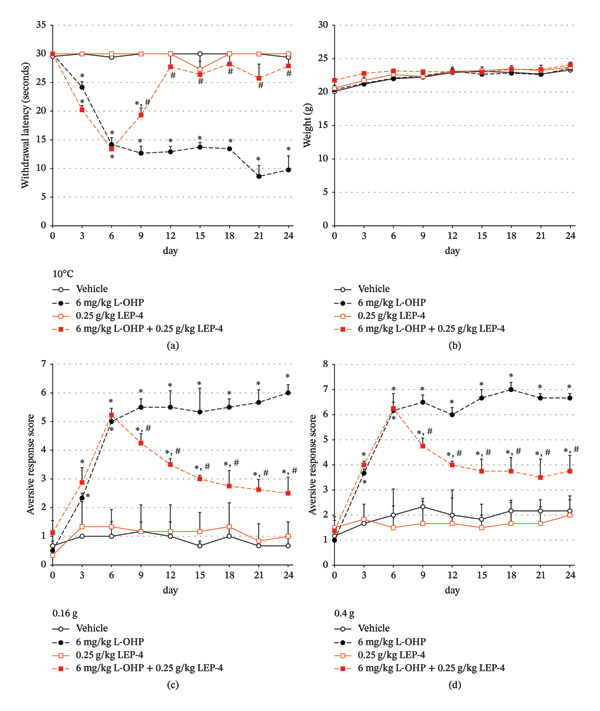

**Figure:**
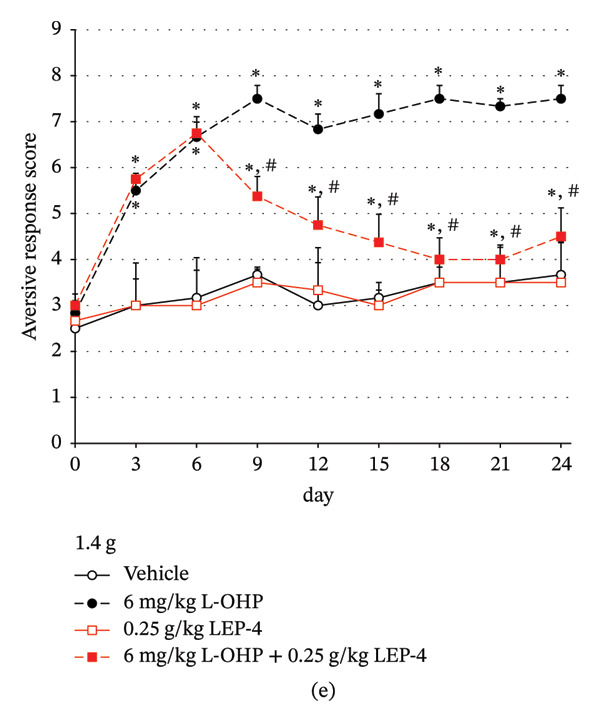

## 4. Discussion

We identified active components in LEM that attenuate chemotherapy‐induced neuropathy by first screening LES, LEP, and LEM(−) to abrogate L‐OHP‐evoked allodynia. Although LES and LEM(−) administration did not attenuate L‐OHP‐evoked neuropathy, LEP administration was effective in attenuating L‐OHP‐evoked neuropathy. Additionally, when fractions containing active components based on molecular weight were collected from LEP, administration of LEP‐4 (molecular weight ≤ 3500) abrogated L‐OHP‐evoked allodynia, whereas no inhibitory effect was observed with LEP‐3 (molecular weight 3500–50,000). Moreover, oral administration of 0.25 g/kg LEP‐4 had no effect on body weight in mice and demonstrated an L‐OHP‐evoked allodynia‐suppressing effect equivalent to that of 30 mg/kg duloxetine. Furthermore, LEP‐4 improved the allodynia induced by L‐OHP administration. LES contains polyphenols and inhibits D‐galactosamine‐induced hepatocyte cell death [24]. In addition, the extracts obtained by treating LEM with 50% ethanol and further treating the solution with 50% methanol have been reported to contain a low‐molecular‐weight compound consisting of lignin, which exhibits hepatoprotective effects [25]. However, in the present study, LES did not have any prophylactic effects against L‐OHP‐evoked allodynia, which suggests that the active constituents of LEM with effects against chemotherapy‐induced neuropathy are not the hydrophobic compounds, polyphenols, or low‐molecular‐weight lignins contained in the ethanol‐soluble fraction LES. Furthermore, the representative constituents in LEM include an arabinoxylan‐like polysaccharide and *α*‐glucan, which have molecular weights of 8500 (as found in LEP‐3) and 250,000, respectively [10], and do not correspond to any of the LEP‐4 candidates. These findings indicate that substances within the molecular weight range of LEP‐3 are not the active components responsible for suppressing chemotherapy‐induced neuropathy, and that the active agents are substances with lower molecular weights. In addition, vanillic and syringic acids have been reported as components of LEM [26]. In streptozotocin‐induced diabetic rats, syringic acid has been shown to enhance motor function at an oral dose of 100 mg/kg [27], whereas vanillic acid suppressed diabetic neuropathy at an oral dose of 50 mg/kg [28]. The LEM used in the present study contained 0.65 mg syringic acid and 1 mg vanillic acid per gram [11], and given that LEP‐4 accounted for 17.1% of the fractionated 100 g LEM, we estimated that 1 g LEM yielded 171 mg LEP‐4. Consequently, the contents of syringic and vanillic acids present in LEP‐4 were assumed to be 0.112 and 0.171 mg, respectively. The dose of LEP‐4 used in this study for L‐OHP‐evoked allodynia was 0.25 g/kg (250 mg/kg), with corresponding amounts of syringic and vanillic acids of 0.163 and 0.25 mg, respectively, which are substantially lower than those previously reported to suppress diabetic neuropathy. Furthermore, although ergothioneine, an antioxidant found in LEM, has been shown to suppress L‐OHP‐induced neuropathy in rats, no correlation between the levels of serum ergothioneine and the prevalence of L‐OHP‐induced peripheral neuropathy has been reported in humans [29–31]. However, whereas botryosferan, a *β*‐glucan extracted from the ascomycete *Botryosphaeria rhodina* MAMB‐05, has been found to have suppressive effects on chronic pain associated with inflammation [32], glucomannan, a *β*‐glucan derived from the lichen *Heterodermia obscurata*, suppresses acute and chronic pain induced by partial sciatic nerve ligation, although given its high molecular weight of between approximately 1,000,000 and 2,000,000, it does not appear to be a candidate constituent of LEP‐4 [33]. LEM also contains a *β*‐glucan, a representative example of which is lentinan, which has a molecular weight of approximately 440,000 [34], and thus similarly would not appear to be a constituent of LEP‐4. On the basis of these findings, we speculate that the active constituent of LEM that attenuates L‐OHP‐evoked allodynia is a substance other than arabinoxylan‐like polysaccharides, *α*‐glucan, *β*‐glucan, syringic acid, and vanillic acid. As this study did not identify the active ingredient, future research is required to clarify this.

The present study demonstrated that LEP‐4 abrogates L‐OHP‐evoked allodynia by attenuating NR2A and Cav3.2 mRNA expression in the DRGs and suppressing ERK1/2 activation in the DRGs and spinal cord. We previously demonstrated that LEM prevents chemotherapy‐induced neuropathy by inhibiting mRNA expression or activating similar molecules [17]. In addition, dimiracetam suppressed L‐OHP‐evoked mechanical allodynia by attenuating NR2A expression [35]. Furthermore, the inhibition of Cav3.2 suppressed L‐OHP‐induced mechanical allodynia [36]. These findings indicate that the downregulation of NR2A, Cav3.2, and phosphorylated ERK1/2 expression in LEP‐4, a fraction of LEM, is likely involved in attenuating L‐OHP‐evoked allodynia.

In this study, we used doses of 1, 1, 0.5, 0.5, 0.25, and 0.25 g/kg for the LEM fractions LEP, LES, LEP‐1, LEP‐2, LEP‐3, and LEP‐4, respectively. Given that the ultimate goal was to identify active compounds in LEM that are effective against chemotherapy‐induced neuropathy, the dosages of these fractions used in this study were determined based on the yield of each fraction. Specifically, as the yields of LES and LEP from LEM were 42.9% and 33.5%, respectively, we estimated that the fractionated material accounted for approximately half of the total, and thus set the dose of LEM at 1 g/kg. This is half the dose at which it had an inhibitory effect on L‐OHP‐evoked allodynia. Similarly, for LEP‐1 and LEP‐2, the yields from LEM were 4.8% and 28.9%, respectively, and given that a majority was isolated as LEP‐2, we concluded that the active constituent was probably contained within this fraction. Consequently, on the basis of the yield of LEP‐2, the dosage was set at 0.5 g/kg, which is one‐fourth of the LEM dosage. Furthermore, applying the same rationale to LEP‐3 and LEP‐4, the percentage recoveries from LEM were 11.7% and 17.1%, respectively. As this indicates that approximately one‐eighth of the LEM is recovered, the dosage was set at 0.25 g/kg, which is one‐eighth of the original amount. The findings of previous studies have indicated that although oral administration of 1 or 2 g/kg of LEM suppresses B16F10 tumor growth [11, 37], it has been demonstrated that administration of 1 g/kg does not suppress chemotherapy‐induced neuropathy, whereas administration of 2 g/kg has been shown to provide significant preventive and therapeutic effects [17]. On the basis of these findings, we believe that LEP‐4, a fraction isolated from LEM, has preventive and therapeutic effects against L‐OHP‐evoked neuropathy at a dosage of 0.25 g/kg.

This study had several limitations. The active ingredient was identified as a substance with a molecular weight of 3500 or less. Based on the LEP‐4 dosage used, the substances and their concentrations in past LEM formulations, and previous studies on the dosage‐related neuropathy suppression, the active ingredient could be a substance other than the arabinoxylan‐like polysaccharide, *α*‐glucan, syringic acid, or vanillic acid. However, as the active constituent of LEM responsible for abrogating chemotherapy‐induced neuropathy has yet to be identified, we initially plan to identify this constituent using the following approach. Firstly, we will subject LEP‐4 to reverse‐phase silica gel column chromatography to obtain the water‐ and methanol‐soluble fractions of LEP‐4. The latter will be separated and purified using high‐performance liquid chromatography (HPLC). If identification of the compounds in this fraction is possible by comparison with standard samples, the HPLC‐purified fraction will be verified by nuclear magnetic resonance. If necessary, further verification can be performed using mass spectrometry. By conducting these analyses to identify candidate compounds and verifying their efficacy in suppressing chemotherapy‐induced neuropathy in mice, we aim to identify the active constituents in LEM. In addition, although we assessed the protective effects of LEM and LEM fractions against L‐OHP‐evoked neuropathy using male mice, we did not perform similar assessments using female mice. In this regard, previous studies using Swiss mice have shown that whereas L‐OHP‐evoked neuropathy occurs regardless of sex, susceptibility differs according to sex. Furthermore, it has been reported that 5‐((4‐methoxyphenyl)thio)benzo[c] [1, 2, 5]thiadiazole, an acetylcholinesterase inhibitor, suppresses L‐OHP‐evoked neuropathy in both male and female mice [38]. Moreover, it has been shown that in C57BL/6J mice, L‐OHP‐evoked neuropathy is induced to similar extents in both males and females, and that this neuropathy is inhibited by the alkaloid mitragynine [39], and, likewise, in BALB/c mice, there are no gender‐related differences in L‐OHP‐evoked neuropathy, and this neuropathy can be suppressed by ELB00824, a peroxisome proliferator‐activated receptor *γ* agonist [40]. On the basis of these findings, it is conceivable that sex‐related differences in effects may be dependent on the strain of mouse assessed. However, given that this was not verified in the present study, in future studies, it will be necessary to investigate sex‐specific susceptibility to L‐OHP‐evoked neuropathy and the inhibitory effects of LEM or LEP‐4 administration. Furthermore, the maximum LEP‐4 administration period in this study was 24 days, and long‐term toxicity was not evaluated. However, a placebo‐controlled Phase 2 trial of LEM for oxaliplatin‐induced peripheral neuropathy reported that 12 weeks of administration demonstrated tolerability, safety, and efficacy in decreasing oxaliplatin‐induced numbness [18]. Therefore, LEM and LEP‐4 may be effective against oxaliplatin‐induced neuropathy and demonstrate high tolerability and safety; however, further studies are warranted to confirm these findings.

## 5. Conclusion

In this study, we demonstrate for the first time that LEP‐4, an ethanol‐insoluble fraction of LEM with a molecular weight of less than 3,500, contains active components that suppress L‐OHP‐evoked neuropathy. We also established that LEP‐4 abrogates the expression of Cav3.2 and NR2A mRNAs, and the activation of ERK1/2 in the DRG and spinal cord of mice. Furthermore, treatment with LEP‐4 contributed to ameliorating allodynia in mice that had developed neuropathy in response to the administration of L‐OHP. Collectively, these findings provide evidence that LEP‐4 may serve as an effective prophylactic and therapeutic agent against L‐OHP‐evoked neuropathy. Given that identifying the active compounds in LEM holds considerable promise for developing therapeutic interventions for L‐OHP‐evoked neuropathy, further studies are warranted.

## Author Contributions

Masanobu Tsubaki: conceptualization, formal analysis, investigation, data curation, writing–original draft, and writing–review and editing. Taira Matsuo: formal analysis, investigation, and data curation. Rie Komori: formal analysis, investigation, and data curation. Tomoya Takeda: formal analysis, investigation, and data curation. Noriaki Nagai: formal analysis and investigation. Toshio Morikawa: investigation, data curation, and writing–review and editing. Shozo Nishida: conceptualization and writing–review and editing.

## Funding

This work was supported in part by a Grant‐in‐Aid for Scientific Research (C) (Grant number 25K10102) from JSPS.

## Conflicts of Interest

The authors declare no conflicts of interest.

## Supporting Information

Additional supporting information can be found online in the Supporting Information section.

## Supporting information


**Supporting Information 1** Supporting Figure 1: Preparation of LEP‐4 from LEM. Details of the preparation method are described in the Materials and Methods section.

**Supporting Information 2** Supporting Table 1: Effect sizes and 95% confidence intervals for significant results in Figure 1a. Supporting Table 2: Effect sizes and 95% confidence intervals for significant results in Figure 1b. Supporting Table 3: Effect sizes and 95% confidence intervals for significant results in Figure 1c. Supporting Table 4: Effect sizes and 95% confidence intervals for significant results in Figure 1d. Supporting Table 5: Effect sizes and 95% confidence intervals for significant results in Figure 1e. Supporting Table 6: Effect sizes and 95% confidence intervals for significant results in Figure 2a. Supporting Table 7: Effect sizes and 95% confidence intervals for significant results in Figure 2c. Supporting Table 8: Effect sizes and 95% confidence intervals for significant results in Figure 2d. Supporting Table 9: Effect sizes and 95% confidence intervals for significant results in Figure 2e. Supporting Table 10: Effect sizes and 95% confidence intervals for significant results in Figure 2b. Supporting Table 11: Effect sizes and 95% confidence intervals for significant results in Figure 3a. Supporting Table 12: Effect sizes and 95% confidence intervals for significant results in Figure 3c. Supporting Table 13: Effect sizes and 95% confidence intervals for significant results in Figure 3d. Supporting Table 14: Effect sizes and 95% confidence intervals for significant results in Figure 3e. Supporting Table 15: Effect sizes and 95% confidence intervals for significant results in Figure 3b. Supporting Table 16: Effect sizes and 95% confidence intervals for significant results in Figure 4a and 4b. Supporting Table 17: Effect sizes and 95% confidence intervals for significant results in Figure 4d and 4f. Supporting Table 18: Effect sizes and 95% confidence intervals for significant results in Figure 5a. Supporting Table 19: Effect sizes and 95% confidence intervals for significant results in Figure 5c. Supporting Table 20: Effect sizes and 95% confidence intervals for significant results in Figure 5d. Supporting Table 21: Effect sizes and 95% confidence intervals for significant results in Figure 5e. Supporting Table 22: Effect sizes and 95% confidence intervals for significant results in Figure 5b.

## Data Availability

The data that support the findings of this study are available from the corresponding author upon reasonable request.
