# Supplementary material for: The Ethanol‐Insoluble Low‐Molecular‐Weight Fraction LEP‐4 Derived From Lentinula edodes Mycelia Extract Attenuates Oxaliplatin‐Evoked Allodynia in Mice
Source: Adv Pharmacol Pharm Sci. 2026 Aug 17;2026:4435116. doi: 10.1155/adpp/4435116 (PMC13479112; doi:10.1155/adpp/4435116)
Supplement: Supplementary file 1 [file ADPP-2026-4435116-s001.docx]

Supporting Figure 1: Preparation of LEP-4 from LEM. Details of the preparation method are described in the Materials and Methods section.
