# Supplementary material for: The Ethanol‐Insoluble Low‐Molecular‐Weight Fraction LEP‐4 Derived From Lentinula edodes Mycelia Extract Attenuates Oxaliplatin‐Evoked Allodynia in Mice
Source: Adv Pharmacol Pharm Sci. 2026 Aug 17;2026:4435116. doi: 10.1155/adpp/4435116 (PMC13479112; doi:10.1155/adpp/4435116)
Supplement: Supplementary file 2 [file ADPP-2026-4435116-s002.docx]

Supporting Table 1: Effect sizes and 95% confident interval for significant results in Figure 1a.

| Days | Comparison | 95% confidence intervals | | P value | *r* |
| --- | --- | --- | --- | --- | --- |
|  |  | Lower limit | Upper limit |  |  |
| 0 day | vehicle vs. 6 mg/kg oxaliplatin | -52.26 | 89.16 | 0.542 | 0.702 |
|  | vehicle vs. 2 g/kg LEM | -70.71 | 70.71 | 1.000 | 0 |
|  | vehicle vs. 1 g/kg LES | -70.71 | 70.71 | 1.000 | 0 |
|  | vehicle vs. 1 g/kg LEP | -70.71 | 70.71 | 1.000 | 0 |
|  | vehicle vs. 1 g/kg LEM(-) | -70.71 | 70.71 | 1.000 | 0 |
|  | vehicle vs. 6 mg/kg oxaliplatin + 2 g/kg LEM | -70.71 | 70.71 | 1.000 | 0 |
|  | vehicle vs. 6 mg/kg oxaliplatin + 1 g/kg LES | -70.71 | 70.71 | 1.000 | 0 |
|  | vehicle vs. 6 mg/kg oxaliplatin + 1 g/kg LEP | -70.71 | 70.71 | 1.000 | 0 |
|  | vehicle vs. 6 mg/kg oxaliplatin + 1 g/kg LEM(-) | -70.71 | 70.71 | 1.000 | 0 |
| 3 day | vehicle vs. 6 mg/kg oxaliplatin | -4.11 | 137.31 | < 0.001 | 1.259 |
|  | vehicle vs. 2 g/kg LEM | -70.71 | 70.71 | 1.000 | 0 |
|  | vehicle vs. 1 g/kg LES | -55.86 | 85.56 | 0.995 | 0.280 |
|  | vehicle vs. 1 g/kg LEP | -70.71 | 70.71 | 1.000 | 0 |
|  | vehicle vs. 1 g/kg LEM(-) | -70.71 | 70.71 | 1.000 | 0 |
|  | vehicle vs. 6 mg/kg oxaliplatin + 2 g/kg LEM | -70.71 | 70.71 | 1.000 | 0 |
|  | vehicle vs. 6 mg/kg oxaliplatin + 1 g/kg LES | -49.26 | 92.16 | 0.945 | 0.405 |
|  | vehicle vs. 6 mg/kg oxaliplatin + 1 g/kg LEP | -70.71 | 70.71 | 1.000 | 0 |
|  | vehicle vs. 6 mg/kg oxaliplatin + 1 g/kg LEM(-) | -41.76 | 99.66 | 0.692 | 0.547 |
| 6 day | vehicle vs. 6 mg/kg oxaliplatin | -9.01 | 132.41 | < 0.001 | 1.207 |
|  | vehicle vs. 2 g/kg LEM | -86.91 | 54.51 | 0.999 | 0.264 |
|  | vehicle vs. 1 g/kg LES | -62.01 | 79.41 | 1.000 | 0.142 |
|  | vehicle vs. 1 g/kg LEP | -86.91 | 54.51 | 0.999 | 0.264 |
|  | vehicle vs. 1 g/kg LEM(-) | -73.11 | 68.31 | 1.000 | 0.039 |
|  | vehicle vs. 6 mg/kg oxaliplatin + 2 g/kg LEM | -68.11 | 73.31 | 1.000 | 0.042 |
|  | vehicle vs. 6 mg/kg oxaliplatin + 1 g/kg LES | -39.71 | 101.71 | 0.986 | 0.506 |
|  | vehicle vs. 6 mg/kg oxaliplatin + 1 g/kg LEP | -71.61 | 69.81 | 1.000 | 0.014 |
|  | vehicle vs. 6 mg/kg oxaliplatin + 1 g/kg LEM(-) | -12.01 | 122.41 | 0.009 | 1.084 |
| 9 day | vehicle vs. 6 mg/kg oxaliplatin | -12.36 | 133.06 | < 0.001 | 1.219 |
|  | vehicle vs. 2 g/kg LEM | -80.16 | 61.26 | 1.000 | 0.183 |
|  | vehicle vs. 1 g/kg LES | -69.81 | 71.61 | 1.000 | 0.017 |
|  | vehicle vs. 1 g/kg LEP | -80.16 | 61.26 | 1.000 | 0.183 |
|  | vehicle vs. 1 g/kg LEM(-) | -80.16 | 61.26 | 1.000 | 0.183 |
|  | vehicle vs. 6 mg/kg oxaliplatin + 2 g/kg LEM | -64.96 | 76.46 | 1.000 | 0.111 |
|  | vehicle vs. 6 mg/kg oxaliplatin + 1 g/kg LES | -18.36 | 120.56 | 0.009 | 1.077 |
|  | vehicle vs. 6 mg/kg oxaliplatin + 1 g/kg LEP | -80.16 | 61.26 | 1.000 | 0.183 |
|  | vehicle vs. 6 mg/kg oxaliplatin + 1 g/kg LEM(-) | -16.26 | 122.16 | 0.008 | 1.086 |
| 12 day | vehicle vs. 6 mg/kg oxaliplatin | -11.36 | 133.06 | < 0.001 | 1.293 |
|  | vehicle vs. 2 g/kg LEM | -79.56 | 61.86 | 1.000 | 0.168 |
|  | vehicle vs. 1 g/kg LES | -68.91 | 72.51 | 1.000 | 0.034 |
|  | vehicle vs. 1 g/kg LEP | -79.56 | 61.86 | 1.000 | 0.168 |
|  | vehicle vs. 1 g/kg LEM(-) | -79.56 | 61.86 | 1.000 | 0.168 |
|  | vehicle vs. 6 mg/kg oxaliplatin + 2 g/kg LEM | -64.96 | 76.46 | 1.000 | 0.109 |
|  | vehicle vs. 6 mg/kg oxaliplatin + 1 g/kg LES | -19.76 | 121.66 | 0.009 | 1.077 |
|  | vehicle vs. 6 mg/kg oxaliplatin + 1 g/kg LEP | -64.66 | 76.76 | 1.000 | 0.114 |
|  | vehicle vs. 6 mg/kg oxaliplatin + 1 g/kg LEM(-) | -19.56 | 131.86 | 0.008 | 1.097 |
| 15 day | vehicle vs. 6 mg/kg oxaliplatin | -10.71 | 130.71 | < 0.001 | 1.298 |
|  | vehicle vs. 2 g/kg LEM | -70.71 | 70.71 | 1.000 | 0 |
|  | vehicle vs. 1 g/kg LES | -70.71 | 70.71 | 1.000 | 0 |
|  | vehicle vs. 1 g/kg LEP | -70.71 | 70.71 | 1.000 | 0 |
|  | vehicle vs. 1 g/kg LEM(-) | -60.96 | 80.46 | 1.000 | 0.192 |
|  | vehicle vs. 6 mg/kg oxaliplatin + 2 g/kg LEM | -63.11 | 78.31 | 1.000 | 0.150 |
|  | vehicle vs. 6 mg/kg oxaliplatin + 1 g/kg LES | -14.31 | 127.11 | 0.003 | 1.113 |
|  | vehicle vs. 6 mg/kg oxaliplatin + 1 g/kg LEP | -60.06 | 81.36 | 1.000 | 0.210 |
|  | vehicle vs. 6 mg/kg oxaliplatin + 1 g/kg LEM(-) | -15.11 | 126.31 | 0.004 | 1.098 |

Supporting Table 2: Effect sizes and 95% confident interval for significant results in Figure 1b.

| Days | Comparison | 95% confidence intervals | | P value | *d* |
| --- | --- | --- | --- | --- | --- |
|  |  | Lower limit | Upper limit |  |  |
| 0 day | vehicle vs. 6 mg/kg oxaliplatin | -1.406 | 0.746 | 0.998 | 0.389 |
|  | vehicle vs. 2 g/kg LEM | -1.986 | 0.366 | 0.346 | 1.288 |
|  | vehicle vs. 1 g/kg LES | -1.696 | 0.645 | 0.860 | 0.673 |
|  | vehicle vs. 1 g/kg LEP | -1.596 | 0.556 | 0.954 | 0.705 |
|  | vehicle vs. 1 g/kg LEM(-) | -0.936 | 1.216 | 1.000 | 0.208 |
|  | vehicle vs. 6 mg/kg oxaliplatin + 2 g/kg LEM | -0.666 | 1.486 | 0.993 | 0.557 |
|  | vehicle vs. 6 mg/kg oxaliplatin + 1 g/kg LES | -1.156 | 0.243 | 0.130 | 1.311 |
|  | vehicle vs. 6 mg/kg oxaliplatin + 1 g/kg LEP | -1.296 | 0.143 | 0.105 | 1.510 |
|  | vehicle vs. 6 mg/kg oxaliplatin + 1 g/kg LEM(-) | -1.696 | 0.456 | 0.860 | 0.742 |
| 3 day | vehicle vs. 6 mg/kg oxaliplatin | -1.431 | 1.211 | 1.000 | 0.131 |
|  | vehicle vs. 2 g/kg LEM | -1.231 | 1.411 | 1.000 | 0.109 |
|  | vehicle vs. 1 g/kg LES | -0.721 | 1.921 | 0.899 | 0.617 |
|  | vehicle vs. 1 g/kg LEP | -1.731 | 0.911 | 0.991 | 0.511 |
|  | vehicle vs. 1 g/kg LEM(-) | -1.551 | 1.091 | 1.000 | 0.373 |
|  | vehicle vs. 6 mg/kg oxaliplatin + 2 g/kg LEM | -0.191 | 2.451 | 0.162 | 1.306 |
|  | vehicle vs. 6 mg/kg oxaliplatin + 1 g/kg LES | -1.481 | 1.161 | 1.000 | 0.159 |
|  | vehicle vs. 6 mg/kg oxaliplatin + 1 g/kg LEP | -1.751 | 0.908 | 0.901 | 0.601 |
|  | vehicle vs. 6 mg/kg oxaliplatin + 1 g/kg LEM(-) | -1.511 | 1.131 | 1.000 | 0.222 |
| 6 day | vehicle vs. 6 mg/kg oxaliplatin | -1.241 | 1.021 | 1.000 | 0.135 |
|  | vehicle vs. 2 g/kg LEM | -1.171 | 1.091 | 1.000 | 0.042 |
|  | vehicle vs. 1 g/kg LES | -0.171 | 2.091 | 0.169 | 1.088 |
|  | vehicle vs. 1 g/kg LEP | -1.781 | 0.481 | 0.693 | 0.842 |
|  | vehicle vs. 1 g/kg LEM(-) | -0.741 | 1.521 | 0.982 | 0.567 |
|  | vehicle vs. 6 mg/kg oxaliplatin + 2 g/kg LEM | -0.538 | 2.801 | 0.165 | 1.867 |
|  | vehicle vs. 6 mg/kg oxaliplatin + 1 g/kg LES | -1.691 | 0.571 | 0.842 | 0.617 |
|  | vehicle vs. 6 mg/kg oxaliplatin + 1 g/kg LEP | -2.231 | 0.131 | 0.164 | 1.361 |
|  | vehicle vs. 6 mg/kg oxaliplatin + 1 g/kg LEM(-) | -1.401 | 0.861 | 0.999 | 0.293 |
| 9 day | vehicle vs. 6 mg/kg oxaliplatin | -1.260 | 1.040 | 1.000 | 0.133 |
|  | vehicle vs. 2 g/kg LEM | -0.200 | 2.100 | 0.198 | 1.161 |
|  | vehicle vs. 1 g/kg LES | -0.259 | 2.860 | 0.151 | 1.801 |
|  | vehicle vs. 1 g/kg LEP | -1.350 | 0.950 | 1.000 | 0.260 |
|  | vehicle vs. 1 g/kg LEM(-) | -1.160 | 1.140 | 1.000 | 0.015 |
|  | vehicle vs. 6 mg/kg oxaliplatin + 2 g/kg LEM | -0.449 | 2.750 | 0.141 | 1.900 |
|  | vehicle vs. 6 mg/kg oxaliplatin + 1 g/kg LES | -1.010 | 1.290 | 1.000 | 0.219 |
|  | vehicle vs. 6 mg/kg oxaliplatin + 1 g/kg LEP | -1.860 | 0.440 | 0.599 | 0.894 |
|  | vehicle vs. 6 mg/kg oxaliplatin + 1 g/kg LEM(-) | -1.100 | 1.200 | 1.000 | 0.063 |
| 12 day | vehicle vs. 6 mg/kg oxaliplatin | -1.377 | 1.077 | 1.000 | 0.166 |
|  | vehicle vs. 2 g/kg LEM | -0.647 | 1.807 | 0.875 | 0.648 |
|  | vehicle vs. 1 g/kg LES | -0.362 | 2.817 | 0.102 | 1.531 |
|  | vehicle vs. 1 g/kg LEP | -1.537 | 0.917 | 0.998 | 0.375 |
|  | vehicle vs. 1 g/kg LEM(-) | -0.717 | 1.737 | 0.939 | 0.706 |
|  | vehicle vs. 6 mg/kg oxaliplatin + 2 g/kg LEM | -0.882 | 3.337 | 0.105 | 2.321 |
|  | vehicle vs. 6 mg/kg oxaliplatin + 1 g/kg LES | -1.357 | 1.097 | 1.000 | 0.153 |
|  | vehicle vs. 6 mg/kg oxaliplatin + 1 g/kg LEP | -2.087 | 0.367 | 0.417 | 0.982 |
|  | vehicle vs. 6 mg/kg oxaliplatin + 1 g/kg LEM(-) | -1.087 | 1.367 | 1.000 | 0.166 |
| 15 day | vehicle vs. 6 mg/kg oxaliplatin | -0.907 | 1.887 | 0.979 | 0.540 |
|  | vehicle vs. 2 g/kg LEM | -0.422 | 3.217 | 0.122 | 2.343 |
|  | vehicle vs. 1 g/kg LES | -0.572 | 3.367 | 0.111 | 1.947 |
|  | vehicle vs. 1 g/kg LEP | -1.637 | 1.157 | 1.000 | 0.284 |
|  | vehicle vs. 1 g/kg LEM(-) | -0.427 | 2.367 | 0.430 | 1.611 |
|  | vehicle vs. 6 mg/kg oxaliplatin + 2 g/kg LEM | -0.502 | 3.297 | 0.101 | 2.403 |
|  | vehicle vs. 6 mg/kg oxaliplatin + 1 g/kg LES | -0.507 | 2.287 | 0.556 | 0.950 |
|  | vehicle vs. 6 mg/kg oxaliplatin + 1 g/kg LEP | -1.767 | 1.027 | 0.997 | 0.392 |
|  | vehicle vs. 6 mg/kg oxaliplatin + 1 g/kg LEM(-) | -0.117 | 2.677 | 0.102 | 1.351 |

Supporting Table 3: Effect sizes and 95% confident interval for significant results in Figure 1c.

| Days | Comparison | 95% confidence intervals | | P value | *d* |
| --- | --- | --- | --- | --- | --- |
|  |  | Lower limit | Upper limit |  |  |
| 0 day | vehicle vs. 6 mg/kg oxaliplatin | -0.556 | 0.156 | 0.720 | 0.435 |
|  | vehicle vs. 2 g/kg LEM | -0.356 | 0.356 | 1.000 | 0 |
|  | vehicle vs. 1 g/kg LES | -0.356 | 0.356 | 1.000 | 0 |
|  | vehicle vs. 1 g/kg LEP | -0.456 | 0.256 | 0.996 | 0.223 |
|  | vehicle vs. 1 g/kg LEM(-) | -0.606 | 0.106 | 0.414 | 0.547 |
|  | vehicle vs. 6 mg/kg oxaliplatin + 2 g/kg LEM | -0.506 | 0.206 | 0.934 | 0.328 |
|  | vehicle vs. 6 mg/kg oxaliplatin + 1 g/kg LES | -0.706 | 0.106 | 0.158 | 0.814 |
|  | vehicle vs. 6 mg/kg oxaliplatin + 1 g/kg LEP | -0.906 | 0.193 | 0.148 | 1.280 |
|  | vehicle vs. 6 mg/kg oxaliplatin + 1 g/kg LEM(-) | -0.606 | 0.106 | 0.414 | 0.464 |
| 3 day | vehicle vs. 6 mg/kg oxaliplatin | -2.911 | -2.188 | < 0.001 | 4.992 |
|  | vehicle vs. 2 g/kg LEM | -0.811 | 0.088 | 0.104 | 0.900 |
|  | vehicle vs. 1 g/kg LES | -0.211 | 0.511 | 0.940 | 0.328 |
|  | vehicle vs. 1 g/kg LEP | -0.611 | 0.111 | 0.437 | 0.547 |
|  | vehicle vs. 1 g/kg LEM(-) | -0.611 | 0.111 | 0.437 | 0.547 |
|  | vehicle vs. 6 mg/kg oxaliplatin + 2 g/kg LEM | -0.311 | 0.411 | 1.000 | 0.100 |
|  | vehicle vs. 6 mg/kg oxaliplatin + 1 g/kg LES | -2.561 | 0.038 | 0.065 | 1.543 |
|  | vehicle vs. 6 mg/kg oxaliplatin + 1 g/kg LEP | -0.711 | 0.111 | 0.267 | 0.593 |
|  | vehicle vs. 6 mg/kg oxaliplatin + 1 g/kg LEM(-) | -2.511 | -1.788 | < 0.001 | 4.301 |
| 6 day | vehicle vs. 6 mg/kg oxaliplatin | -5.405 | -3.794 | < 0.001 | 5.923 |
|  | vehicle vs. 2 g/kg LEM | -1.405 | 0.205 | 0.329 | 0.929 |
|  | vehicle vs. 1 g/kg LES | -0.805 | 0.805 | 1.000 | 0 |
|  | vehicle vs. 1 g/kg LEP | -0.955 | 0.655 | 1.000 | 0.275 |
|  | vehicle vs. 1 g/kg LEM(-) | -1.205 | 0.405 | 0.839 | 0.755 |
|  | vehicle vs. 6 mg/kg oxaliplatin + 2 g/kg LEM | -1.105 | 0.505 | 0.969 | 0.420 |
|  | vehicle vs. 6 mg/kg oxaliplatin + 1 g/kg LES | -3.755 | -2.144 | < 0.001 | 5.426 |
|  | vehicle vs. 6 mg/kg oxaliplatin + 1 g/kg LEP | -1.105 | 0.505 | 0.969 | 0.398 |
|  | vehicle vs. 6 mg/kg oxaliplatin + 1 g/kg LEM(-) | -4.205 | -2.594 | < 0.001 | 4.402 |
| 9 day | vehicle vs. 6 mg/kg oxaliplatin | -5.067 | -3.633 | < 0.001 | 8.079 |
|  | vehicle vs. 2 g/kg LEM | -1.117 | 0.317 | 0.727 | 0.595 |
|  | vehicle vs. 1 g/kg LES | -1.167 | 0.267 | 0.576 | 0.852 |
|  | vehicle vs. 1 g/kg LEP | -0.617 | 0.817 | 1.000 | 0.158 |
|  | vehicle vs. 1 g/kg LEM(-) | -0.817 | 0.617 | 1.000 | 0.166 |
|  | vehicle vs. 6 mg/kg oxaliplatin + 2 g/kg LEM | -0.817 | 0.617 | 1.000 | 0.142 |
|  | vehicle vs. 6 mg/kg oxaliplatin + 1 g/kg LES | -4.267 | -2.833 | < 0.001 | 5.393 |
|  | vehicle vs. 6 mg/kg oxaliplatin + 1 g/kg LEP | -1.017 | 0.417 | 0.937 | 0.397 |
|  | vehicle vs. 6 mg/kg oxaliplatin + 1 g/kg LEM(-) | -4.917 | -3.483 | < 0.001 | 8.181 |
| 12 day | vehicle vs. 6 mg/kg oxaliplatin | -5.067 | -3.633 | < 0.001 | 8.079 |
|  | vehicle vs. 2 g/kg LEM | -1.117 | 0.317 | 0.727 | 0.595 |
|  | vehicle vs. 1 g/kg LES | -1.167 | 0.267 | 0.576 | 0.852 |
|  | vehicle vs. 1 g/kg LEP | -0.617 | 0.817 | 1.000 | 0.158 |
|  | vehicle vs. 1 g/kg LEM(-) | -0.817 | 0.617 | 1.000 | 0.166 |
|  | vehicle vs. 6 mg/kg oxaliplatin + 2 g/kg LEM | -0.817 | 0.617 | 1.000 | 0.142 |
|  | vehicle vs. 6 mg/kg oxaliplatin + 1 g/kg LES | -4.267 | -2.833 | < 0.001 | 5.393 |
|  | vehicle vs. 6 mg/kg oxaliplatin + 1 g/kg LEP | -1.017 | 0.417 | 0.937 | 0.397 |
|  | vehicle vs. 6 mg/kg oxaliplatin + 1 g/kg LEM(-) | -4.917 | -3.483 | < 0.001 | 8.181 |
| 15 day | vehicle vs. 6 mg/kg oxaliplatin | -6.141 | -4.158 | < 0.001 | 8.043 |
|  | vehicle vs. 2 g/kg LEM | -1.491 | 0.491 | 0.827 | 0.821 |
|  | vehicle vs. 1 g/kg LES | -1.691 | 0.291 | 0.406 | 1.212 |
|  | vehicle vs. 1 g/kg LEP | -1.241 | 0.741 | 0.998 | 0.278 |
|  | vehicle vs. 1 g/kg LEM(-) | -1.141 | 0.841 | 1.000 | 0.213 |
|  | vehicle vs. 6 mg/kg oxaliplatin + 2 g/kg LEM | -1.241 | 0.741 | 0.998 | 0.303 |
|  | vehicle vs. 6 mg/kg oxaliplatin + 1 g/kg LES | -4.541 | -2.558 | < 0.001 | 5.694 |
|  | vehicle vs. 6 mg/kg oxaliplatin + 1 g/kg LEP | -1.341 | 0.641 | 0.978 | 0.425 |
|  | vehicle vs. 6 mg/kg oxaliplatin + 1 g/kg LEM(-) | -5.891 | -3.908 | < 0.001 | 6.710 |

Supporting Table 4: Effect sizes and 95% confident interval for significant results in Figure 1d.

| Days | Comparison | 95% confidence intervals | | P value | *d* |
| --- | --- | --- | --- | --- | --- |
|  |  | Lower limit | Upper limit |  |  |
| 0 day | vehicle vs. 6 mg/kg oxaliplatin | -0.810 | 0.410 | 0.987 | 0.303 |
|  | vehicle vs. 2 g/kg LEM | -0.810 | 0.410 | 0.987 | 0.321 |
|  | vehicle vs. 1 g/kg LES | -0.810 | 0.410 | 0.987 | 0.303 |
|  | vehicle vs. 1 g/kg LEP | -0.460 | 0.760 | 0.998 | 0.237 |
|  | vehicle vs. 1 g/kg LEM(-) | -0.560 | 0.660 | 1.000 | 0.072 |
|  | vehicle vs. 6 mg/kg oxaliplatin + 2 g/kg LEM | -0.560 | 0.660 | 1.000 | 0.063 |
|  | vehicle vs. 6 mg/kg oxaliplatin + 1 g/kg LES | -0.610 | 0.610 | 1.000 | 0 |
|  | vehicle vs. 6 mg/kg oxaliplatin + 1 g/kg LEP | -0.760 | 0.460 | 0.998 | 0.240 |
|  | vehicle vs. 6 mg/kg oxaliplatin + 1 g/kg LEM(-) | -0.860 | 0.360 | 0.944 | 0.406 |
| 3 day | vehicle vs. 6 mg/kg oxaliplatin | -3.329 | -1.870 | < 0.001 | 3.686 |
|  | vehicle vs. 2 g/kg LEM | -0.679 | 0.779 | 1.000 | 0.075 |
|  | vehicle vs. 1 g/kg LES | -0.729 | 0.729 | 1.000 | 0 |
|  | vehicle vs. 1 g/kg LEP | -0.379 | 1.079 | 0.865 | 0.486 |
|  | vehicle vs. 1 g/kg LEM(-) | -0.679 | 0.779 | 1.000 | 0.075 |
|  | vehicle vs. 6 mg/kg oxaliplatin + 2 g/kg LEM | -0.629 | 0.829 | 1.000 | 0.112 |
|  | vehicle vs. 6 mg/kg oxaliplatin + 1 g/kg LES | -3.229 | -1.770 | < 0.001 | 3.220 |
|  | vehicle vs. 6 mg/kg oxaliplatin + 1 g/kg LEP | -1.079 | 0.379 | 0.865 | 0.483 |
|  | vehicle vs. 6 mg/kg oxaliplatin + 1 g/kg LEM(-) | -3.029 | -1.570 | < 0.001 | 3.492 |
| 6 day | vehicle vs. 6 mg/kg oxaliplatin | -5.287 | -3.112 | < 0.001 | 4.730 |
|  | vehicle vs. 2 g/kg LEM | -0.987 | 1.187 | 1.000 | 0.157 |
|  | vehicle vs. 1 g/kg LES | -0.587 | 1.587 | 0.892 | 0.645 |
|  | vehicle vs. 1 g/kg LEP | -0.137 | 2.037 | 0.141 | 1.427 |
|  | vehicle vs. 1 g/kg LEM(-) | -0.587 | 1.587 | 0.892 | 0.787 |
|  | vehicle vs. 6 mg/kg oxaliplatin + 2 g/kg LEM | -0.837 | 1.337 | 0.999 | 0.272 |
|  | vehicle vs. 6 mg/kg oxaliplatin + 1 g/kg LES | -4.437 | -2.262 | < 0.001 | 4.430 |
|  | vehicle vs. 6 mg/kg oxaliplatin + 1 g/kg LEP | -0.737 | 1.437 | 0.989 | 0.556 |
|  | vehicle vs. 6 mg/kg oxaliplatin + 1 g/kg LEM(-) | -4.687 | -2.512 | < 0.001 | 5.762 |
| 9 day | vehicle vs. 6 mg/kg oxaliplatin | -5.073 | -3.026 | < 0.001 | 5.449 |
|  | vehicle vs. 2 g/kg LEM | -0.873 | 1.173 | 1.000 | 0.186 |
|  | vehicle vs. 1 g/kg LES | -0.623 | 1.423 | 0.958 | 0.604 |
|  | vehicle vs. 1 g/kg LEP | -0.273 | 1.773 | 0.352 | 1.271 |
|  | vehicle vs. 1 g/kg LEM(-) | -0.973 | 1.073 | 1.000 | 0.067 |
|  | vehicle vs. 6 mg/kg oxaliplatin + 2 g/kg LEM | -0.873 | 1.173 | 1.000 | 0.209 |
|  | vehicle vs. 6 mg/kg oxaliplatin + 1 g/kg LES | -4.973 | -2.926 | < 0.001 | 4.935 |
|  | vehicle vs. 6 mg/kg oxaliplatin + 1 g/kg LEP | -0.823 | 1.223 | 1.000 | 0.279 |
|  | vehicle vs. 6 mg/kg oxaliplatin + 1 g/kg LEM(-) | -5.873 | -3.826 | < 0.001 | 5.312 |
| 12 day | vehicle vs. 6 mg/kg oxaliplatin | -5.073 | -3.026 | < 0.001 | 5.449 |
|  | vehicle vs. 2 g/kg LEM | -0.873 | 1.173 | 1.000 | 0.186 |
|  | vehicle vs. 1 g/kg LES | -0.623 | 1.423 | 0.958 | 0.604 |
|  | vehicle vs. 1 g/kg LEP | -0.273 | 1.773 | 0.352 | 1.271 |
|  | vehicle vs. 1 g/kg LEM(-) | -0.973 | 1.073 | 1.000 | 0.067 |
|  | vehicle vs. 6 mg/kg oxaliplatin + 2 g/kg LEM | -0.873 | 1.173 | 1.000 | 0.209 |
|  | vehicle vs. 6 mg/kg oxaliplatin + 1 g/kg LES | -4.973 | -2.926 | < 0.001 | 4.935 |
|  | vehicle vs. 6 mg/kg oxaliplatin + 1 g/kg LEP | -0.823 | 1.223 | 1.000 | 0.279 |
|  | vehicle vs. 6 mg/kg oxaliplatin + 1 g/kg LEM(-) | -5.873 | -3.826 | < 0.001 | 5.312 |
| 15 day | vehicle vs. 6 mg/kg oxaliplatin | -5.709 | -2.991 | < 0.001 | 6.794 |
|  | vehicle vs. 2 g/kg LEM | -0.959 | 1.759 | 0.994 | 0.482 |
|  | vehicle vs. 1 g/kg LES | -0.809 | 1.909 | 0.948 | 0.673 |
|  | vehicle vs. 1 g/kg LEP | -0.759 | 1.959 | 0.914 | 0.821 |
|  | vehicle vs. 1 g/kg LEM(-) | -1.309 | 1.409 | 1.000 | 0.076 |
|  | vehicle vs. 6 mg/kg oxaliplatin + 2 g/kg LEM | -0.509 | 2.209 | 0.581 | 0.848 |
|  | vehicle vs. 6 mg/kg oxaliplatin + 1 g/kg LES | -5.559 | -2.841 | < 0.001 | 4.743 |
|  | vehicle vs. 6 mg/kg oxaliplatin + 1 g/kg LEP | -0.509 | 2.209 | 0.581 | 0.940 |
|  | vehicle vs. 6 mg/kg oxaliplatin + 1 g/kg LEM(-) | -6.509 | -3.791 | < 0.001 | 6.671 |

Supporting Table 5: Effect sizes and 95% confident interval for significant results in Figure 1e.

| Days | Comparison | 95% confidence intervals | | P value | *d* |
| --- | --- | --- | --- | --- | --- |
|  |  | Lower limit | Upper limit |  |  |
| 0 day | vehicle vs. 6 mg/kg oxaliplatin | -0.475 | 0.275 | 0.997 | 0.198 |
|  | vehicle vs. 2 g/kg LEM | -0.575 | 0.175 | 0.776 | 0.406 |
|  | vehicle vs. 1 g/kg LES | -0.425 | 0.325 | 1.000 | 0.100 |
|  | vehicle vs. 1 g/kg LEP | -0.274 | 1.025 | 0.152 | 0.870 |
|  | vehicle vs. 1 g/kg LEM(-) | -0.525 | 0.225 | 0.952 | 0.275 |
|  | vehicle vs. 6 mg/kg oxaliplatin + 2 g/kg LEM | -0.625 | 0.125 | 0.491 | 0.525 |
|  | vehicle vs. 6 mg/kg oxaliplatin + 1 g/kg LES | -0.475 | 0.275 | 0.997 | 0.198 |
|  | vehicle vs. 6 mg/kg oxaliplatin + 1 g/kg LEP | -0.625 | 0.125 | 0.491 | 0.525 |
|  | vehicle vs. 6 mg/kg oxaliplatin + 1 g/kg LEM(-) | -0.675 | 0.075 | 0.236 | 0.543 |
| 3 day | vehicle vs. 6 mg/kg oxaliplatin | -3.384 | -2.015 | < 0.001 | 3.827 |
|  | vehicle vs. 2 g/kg LEM | -0.634 | 0.734 | 1.000 | 0.076 |
|  | vehicle vs. 1 g/kg LES | -0.734 | 0.634 | 1.000 | 0.076 |
|  | vehicle vs. 1 g/kg LEP | -0.115 | 1.484 | 0.111 | 0.898 |
|  | vehicle vs. 1 g/kg LEM(-) | -0.834 | 0.534 | 0.999 | 0.263 |
|  | vehicle vs. 6 mg/kg oxaliplatin + 2 g/kg LEM | -0.834 | 0.534 | 0.999 | 0.263 |
|  | vehicle vs. 6 mg/kg oxaliplatin + 1 g/kg LES | -2.934 | -1.565 | < 0.001 | 3.882 |
|  | vehicle vs. 6 mg/kg oxaliplatin + 1 g/kg LEP | -0.884 | 0.484 | 0.994 | 0.283 |
|  | vehicle vs. 6 mg/kg oxaliplatin + 1 g/kg LEM(-) | -2.984 | -1.615 | < 0.001 | 3.018 |
| 6 day | vehicle vs. 6 mg/kg oxaliplatin | -6.381 | -4.218 | < 0.001 | 7.172 |
|  | vehicle vs. 2 g/kg LEM | -1.081 | 1.081 | 1.000 | 0 |
|  | vehicle vs. 1 g/kg LES | -0.881 | 1.281 | 1.000 | 0.248 |
|  | vehicle vs. 1 g/kg LEP | -0.031 | 2.131 | 0.065 | 0.697 |
|  | vehicle vs. 1 g/kg LEM(-) | -0.931 | 1.231 | 1.000 | 0.226 |
|  | vehicle vs. 6 mg/kg oxaliplatin + 2 g/kg LEM | -1.531 | 0.631 | 0.939 | 0.556 |
|  | vehicle vs. 6 mg/kg oxaliplatin + 1 g/kg LES | -5.481 | -3.318 | < 0.001 | 4.995 |
|  | vehicle vs. 6 mg/kg oxaliplatin + 1 g/kg LEP | -1.331 | 0.831 | 0.999 | 0.258 |
|  | vehicle vs. 6 mg/kg oxaliplatin + 1 g/kg LEM(-) | -6.381 | -4.218 | < 0.001 | 5.928 |
| 9 day | vehicle vs. 6 mg/kg oxaliplatin | -5.739 | -3.860 | < 0.001 | 7.211 |
|  | vehicle vs. 2 g/kg LEM | -1.089 | 0.789 | 1.000 | 0.225 |
|  | vehicle vs. 1 g/kg LES | -1.239 | 0.639 | 0.989 | 0.404 |
|  | vehicle vs. 1 g/kg LEP | -0.889 | 0.989 | 1.000 | 0.057 |
|  | vehicle vs. 1 g/kg LEM(-) | -1.089 | 0.789 | 1.000 | 0.225 |
|  | vehicle vs. 6 mg/kg oxaliplatin + 2 g/kg LEM | -1.339 | 0.539 | 0.930 | 0.561 |
|  | vehicle vs. 6 mg/kg oxaliplatin + 1 g/kg LES | -5.889 | -4.010 | < 0.001 | 5.560 |
|  | vehicle vs. 6 mg/kg oxaliplatin + 1 g/kg LEP | -1.389 | 0.489 | 0.866 | 0.617 |
|  | vehicle vs. 6 mg/kg oxaliplatin + 1 g/kg LEM(-) | -7.039 | -5.160 | < 0.001 | 9.394 |
| 12 day | vehicle vs. 6 mg/kg oxaliplatin | -5.739 | -3.860 | < 0.001 | 7.211 |
|  | vehicle vs. 2 g/kg LEM | -1.089 | 0.789 | 1.000 | 0.225 |
|  | vehicle vs. 1 g/kg LES | -1.239 | 0.639 | 0.989 | 0.404 |
|  | vehicle vs. 1 g/kg LEP | -0.889 | 0.989 | 1.000 | 0.057 |
|  | vehicle vs. 1 g/kg LEM(-) | -1.089 | 0.789 | 1.000 | 0.225 |
|  | vehicle vs. 6 mg/kg oxaliplatin + 2 g/kg LEM | -1.339 | 0.539 | 0.930 | 0.561 |
|  | vehicle vs. 6 mg/kg oxaliplatin + 1 g/kg LES | -5.889 | -4.010 | < 0.001 | 5.560 |
|  | vehicle vs. 6 mg/kg oxaliplatin + 1 g/kg LEP | -1.389 | 0.489 | 0.866 | 0.617 |
|  | vehicle vs. 6 mg/kg oxaliplatin + 1 g/kg LEM(-) | -7.039 | -5.160 | < 0.001 | 9.394 |
| 15 day | vehicle vs. 6 mg/kg oxaliplatin | -6.442 | -4.157 | < 0.001 | 6.636 |
|  | vehicle vs. 2 g/kg LEM | -1.342 | 0.942 | 1.000 | 0.223 |
|  | vehicle vs. 1 g/kg LES | -1.642 | 0.642 | 0.918 | 0.607 |
|  | vehicle vs. 1 g/kg LEP | -1.142 | 1.142 | 1.000 | 0 |
|  | vehicle vs. 1 g/kg LEM(-) | -1.542 | 0.742 | 0.980 | 0.590 |
|  | vehicle vs. 6 mg/kg oxaliplatin + 2 g/kg LEM | -1.542 | 0.742 | 0.980 | 0.504 |
|  | vehicle vs. 6 mg/kg oxaliplatin + 1 g/kg LES | -6.342 | -4.057 | < 0.001 | 5.174 |
|  | vehicle vs. 6 mg/kg oxaliplatin + 1 g/kg LEP | -1.692 | 0.592 | 0.862 | 0.691 |
|  | vehicle vs. 6 mg/kg oxaliplatin + 1 g/kg LEM(-) | -7.592 | -5.307 | < 0.001 | 9.432 |

Supporting Table 6: Effect sizes and 95% confident interval for significant results in Figure 2a.

| Days | Comparison | 95% confidence intervals | | P value | *r* |
| --- | --- | --- | --- | --- | --- |
|  |  | Lower limit | Upper limit |  |  |
| 0 day | vehicle vs. 6 mg/kg oxaliplatin | -70.212 | 71.212 | 1.000 | 0.016 |
|  | vehicle vs. 2 g/kg LEM | -70.312 | 71.112 | 1.000 | 0.013 |
|  | vehicle vs. 1 g/kg LEP | -70.912 | 70.512 | 1.000 | 0.006 |
|  | vehicle vs. 0.5 g/kg LEP-1 | -70.512 | 70.912 | 1.000 | 0.006 |
|  | vehicle vs. 0.5 g/kg LEP-2 | -71.012 | 70.412 | 1.000 | 0.009 |
|  | vehicle vs. 6 mg/kg oxaliplatin + 2 g/kg LEM | -71.112 | 70.312 | 1.000 | 0.013 |
|  | vehicle vs. 6 mg/kg oxaliplatin + 1 g/kg LEP | -70.612 | 70.812 | 1.000 | 0.003 |
|  | vehicle vs. 6 mg/kg oxaliplatin + 0.5 g/kg LEP-1 | -70.412 | 71.012 | 1.000 | 0.009 |
|  | vehicle vs. 6 mg/kg oxaliplatin + 0.5 g/kg LEP-2 | -70.812 | 70.612 | 1.000 | 0.003 |
| 3 day | vehicle vs. 6 mg/kg oxaliplatin | -14.612 | 126.812 | < 0.001 | 1.237 |
|  | vehicle vs. 2 g/kg LEM | -59.312 | 82.112 | 0.999 | 0.251 |
|  | vehicle vs. 1 g/kg LEP | -70.712 | 70.712 | 1.000 | 0 |
|  | vehicle vs. 0.5 g/kg LEP-1 | -62.612 | 78.812 | 1.000 | 0.178 |
|  | vehicle vs. 0.5 g/kg LEP-2 | -70.712 | 70.712 | 1.000 | 0 |
|  | vehicle vs. 6 mg/kg oxaliplatin + 2 g/kg LEM | -61.812 | 79.612 | 1.000 | 0.196 |
|  | vehicle vs. 6 mg/kg oxaliplatin + 1 g/kg LEP | -70.712 | 70.712 | 1.000 | 0 |
|  | vehicle vs. 6 mg/kg oxaliplatin + 0.5 g/kg LEP-1 | -20.212 | 121.212 | 0.003 | 1.113 |
|  | vehicle vs. 6 mg/kg oxaliplatin + 0.5 g/kg LEP-2 | -70.712 | 70.712 | 1.000 | 0 |
| 6 day | vehicle vs. 6 mg/kg oxaliplatin | -17.312 | 124.112 | < 0.001 | 1.294 |
|  | vehicle vs. 2 g/kg LEM | -83.612 | 57.812 | 0.998 | 0.265 |
|  | vehicle vs. 1 g/kg LEP | -71.612 | 69.812 | 1.000 | 0.018 |
|  | vehicle vs. 0.5 g/kg LEP-1 | -72.512 | 68.912 | 1.000 | 0.037 |
|  | vehicle vs. 0.5 g/kg LEP-2 | -83.612 | 57.812 | 0.998 | 0.265 |
|  | vehicle vs. 6 mg/kg oxaliplatin + 2 g/kg LEM | -76.712 | 64.712 | 1.000 | 0.123 |
|  | vehicle vs. 6 mg/kg oxaliplatin + 1 g/kg LEP | -83.612 | 57.812 | 0.998 | 0.265 |
|  | vehicle vs. 6 mg/kg oxaliplatin + 0.5 g/kg LEP-1 | -16.912 | 124.512 | < 0.001 | 1.102 |
|  | vehicle vs. 6 mg/kg oxaliplatin + 0.5 g/kg LEP-2 | -74.512 | 66.912 | 1.000 | 0.078 |
| 9 day | vehicle vs. 6 mg/kg oxaliplatin | -14.662 | 126.762 | < 0.001 | 1.324 |
|  | vehicle vs. 2 g/kg LEM | -81.662 | 59.762 | 1.000 | 0.219 |
|  | vehicle vs. 1 g/kg LEP | -73.062 | 68.362 | 1.000 | 0.047 |
|  | vehicle vs. 0.5 g/kg LEP-1 | -62.612 | 78.812 | 1.000 | 0.162 |
|  | vehicle vs. 0.5 g/kg LEP-2 | -81.662 | 59.762 | 1.000 | 0.219 |
|  | vehicle vs. 6 mg/kg oxaliplatin + 2 g/kg LEM | -73.862 | 67.562 | 1.000 | 0.063 |
|  | vehicle vs. 6 mg/kg oxaliplatin + 1 g/kg LEP | -73.462 | 67.962 | 1.000 | 0.055 |
|  | vehicle vs. 6 mg/kg oxaliplatin + 0.5 g/kg LEP-1 | -12.662 | 128.762 | < 0.001 | 1.264 |
|  | vehicle vs. 6 mg/kg oxaliplatin + 0.5 g/kg LEP-2 | -72.262 | 69.162 | 1.000 | 0.031 |
| 12 day | vehicle vs. 6 mg/kg oxaliplatin | -14.912 | 126.512 | < 0.001 | 1.161 |
|  | vehicle vs. 2 g/kg LEM | -61.712 | 79.712 | 1.000 | 0.187 |
|  | vehicle vs. 1 g/kg LEP | -63.312 | 78.112 | 1.000 | 0.154 |
|  | vehicle vs. 0.5 g/kg LEP-1 | -62.512 | 78.912 | 1.000 | 0.170 |
|  | vehicle vs. 0.5 g/kg LEP-2 | -70.712 | 70.712 | 1.000 | 0 |
|  | vehicle vs. 6 mg/kg oxaliplatin + 2 g/kg LEM | -62.112 | 79.312 | 1.000 | 0.179 |
|  | vehicle vs. 6 mg/kg oxaliplatin + 1 g/kg LEP | -62.912 | 78.512 | 1.000 | 0.162 |
|  | vehicle vs. 6 mg/kg oxaliplatin + 0.5 g/kg LEP-1 | -14.512 | 126.912 | 0.001 | 1.169 |
|  | vehicle vs. 6 mg/kg oxaliplatin + 0.5 g/kg LEP-2 | -63.712 | 77.712 | 1.000 | 0.145 |
| 15 day | vehicle vs. 6 mg/kg oxaliplatin | -17.912 | 123.512 | < 0.001 | 1.042 |
|  | vehicle vs. 2 g/kg LEM | -63.112 | 78.312 | 1.000 | 0.150 |
|  | vehicle vs. 1 g/kg LEP | -69.112 | 72.312 | 1.000 | 0.031 |
|  | vehicle vs. 0.5 g/kg LEP-1 | -69.912 | 71.512 | 1.000 | 0.015 |
|  | vehicle vs. 0.5 g/kg LEP-2 | -78.712 | 62.712 | 1.000 | 0.157 |
|  | vehicle vs. 6 mg/kg oxaliplatin + 2 g/kg LEM | -71.912 | 69.512 | 1.000 | 0.023 |
|  | vehicle vs. 6 mg/kg oxaliplatin + 1 g/kg LEP | -70.312 | 71.112 | 1.000 | 0.007 |
|  | vehicle vs. 6 mg/kg oxaliplatin + 0.5 g/kg LEP-1 | -15.512 | 129.912 | < 0.001 | 1.071 |
|  | vehicle vs. 6 mg/kg oxaliplatin + 0.5 g/kg LEP-2 | -63.912 | 77.512 | 1.000 | 0.134 |

Supporting Table 7: Effect sizes and 95% confident interval for significant results in Figure 2c.

| Days | Comparison | 95% confidence intervals | | P value | *d* |
| --- | --- | --- | --- | --- | --- |
|  |  | Lower limit | Upper limit |  |  |
| 0 day | vehicle vs. 6 mg/kg oxaliplatin | -0.876 | 0.276 | 0.799 | 0.424 |
|  | vehicle vs. 2 g/kg LEM | -0.476 | 0.676 | 1.000 | 0.159 |
|  | vehicle vs. 1 g/kg LEP | -0.776 | 0.376 | 0.981 | 0.309 |
|  | vehicle vs. 0.5 g/kg LEP-1 | -0.876 | 0.276 | 0.799 | 0.464 |
|  | vehicle vs. 0.5 g/kg LEP-2 | -0.376 | 0.776 | 0.981 | 0.309 |
|  | vehicle vs. 6 mg/kg oxaliplatin + 2 g/kg LEM | -1.176 | 0.223 | 0.334 | 0.811 |
|  | vehicle vs. 6 mg/kg oxaliplatin + 1 g/kg LEP | -0.476 | 0.676 | 1.000 | 0.143 |
|  | vehicle vs. 6 mg/kg oxaliplatin + 0.5 g/kg LEP-1 | -0.576 | 0.576 | 1.000 | 0 |
|  | vehicle vs. 6 mg/kg oxaliplatin + 0.5 g/kg LEP-2 | -0.776 | 0.376 | 0.981 | 0.267 |
| 3 day | vehicle vs. 6 mg/kg oxaliplatin | -4.360 | -2.039 | < 0.001 | 3.471 |
|  | vehicle vs. 2 g/kg LEM | -1.860 | 0.460 | 0.631 | 0.774 |
|  | vehicle vs. 1 g/kg LEP | -2.160 | 0.160 | 0.154 | 1.111 |
|  | vehicle vs. 0.5 g/kg LEP-1 | -1.760 | 0.560 | 0.805 | 0.679 |
|  | vehicle vs. 0.5 g/kg LEP-2 | -1.160 | 1.160 | 1.000 | 0 |
|  | vehicle vs. 6 mg/kg oxaliplatin + 2 g/kg LEM | -1.160 | 1.160 | 1.000 | 0 |
|  | vehicle vs. 6 mg/kg oxaliplatin + 1 g/kg LEP | -1.360 | 0.960 | 1.000 | 0.213 |
|  | vehicle vs. 6 mg/kg oxaliplatin + 0.5 g/kg LEP-1 | -4.760 | -2.439 | < 0.001 | 3.772 |
|  | vehicle vs. 6 mg/kg oxaliplatin + 0.5 g/kg LEP-2 | -1.560 | 0.760 | 0.982 | 0.416 |
| 6 day | vehicle vs. 6 mg/kg oxaliplatin | -5.246 | -3.554 | < 0.001 | 6.467 |
|  | vehicle vs. 2 g/kg LEM | -1.146 | 0.546 | 0.978 | 0.462 |
|  | vehicle vs. 1 g/kg LEP | -0.846 | 0.846 | 1.000 | 0 |
|  | vehicle vs. 0.5 g/kg LEP-1 | -0.846 | 0.846 | 1.000 | 0 |
|  | vehicle vs. 0.5 g/kg LEP-2 | -0.546 | 1.146 | 0.978 | 0.545 |
|  | vehicle vs. 6 mg/kg oxaliplatin + 2 g/kg LEM | -0.646 | 1.046 | 0.999 | 0.293 |
|  | vehicle vs. 6 mg/kg oxaliplatin + 1 g/kg LEP | -0.746 | 0.946 | 1.000 | 0.163 |
|  | vehicle vs. 6 mg/kg oxaliplatin + 0.5 g/kg LEP-1 | -5.446 | -3.754 | < 0.001 | 6.223 |
|  | vehicle vs. 6 mg/kg oxaliplatin + 0.5 g/kg LEP-2 | -1.246 | 0.446 | 0.875 | 0.587 |
| 9 day | vehicle vs. 6 mg/kg oxaliplatin | -5.262 | -3.337 | < 0.001 | 5.787 |
|  | vehicle vs. 2 g/kg LEM | -0.862 | 1.062 | 1.000 | 0.151 |
|  | vehicle vs. 1 g/kg LEP | -0.962 | 0.962 | 1.000 | 0 |
|  | vehicle vs. 0.5 g/kg LEP-1 | -1.062 | 0.862 | 1.000 | 0.151 |
|  | vehicle vs. 0.5 g/kg LEP-2 | -0.662 | 1.262 | 0.991 | 0.496 |
|  | vehicle vs. 6 mg/kg oxaliplatin + 2 g/kg LEM | -1.162 | 0.762 | 1.000 | 0.251 |
|  | vehicle vs. 6 mg/kg oxaliplatin + 1 g/kg LEP | -0.662 | 1.262 | 0.991 | 0.325 |
|  | vehicle vs. 6 mg/kg oxaliplatin + 0.5 g/kg LEP-1 | -5.062 | -3.137 | < 0.001 | 4.836 |
|  | vehicle vs. 6 mg/kg oxaliplatin + 0.5 g/kg LEP-2 | -0.962 | 0.962 | 1.000 | 0 |
| 12 day | vehicle vs. 6 mg/kg oxaliplatin | -5.374 | -3.425 | < 0.001 | 7.009 |
|  | vehicle vs. 2 g/kg LEM | -1.474 | 0.474 | 0.812 | 0.829 |
|  | vehicle vs. 1 g/kg LEP | -0.674 | 1.274 | 0.992 | 0.619 |
|  | vehicle vs. 0.5 g/kg LEP-1 | -1.174 | 0.774 | 1.000 | 0.308 |
|  | vehicle vs. 0.5 g/kg LEP-2 | -0.774 | 1.174 | 1.000 | 0.331 |
|  | vehicle vs. 6 mg/kg oxaliplatin + 2 g/kg LEM | -0.974 | 0.974 | 1.000 | 0 |
|  | vehicle vs. 6 mg/kg oxaliplatin + 1 g/kg LEP | -0.474 | 1.474 | 0.812 | 0.636 |
|  | vehicle vs. 6 mg/kg oxaliplatin + 0.5 g/kg LEP-1 | -5.774 | -3.825 | < 0.001 | 6.113 |
|  | vehicle vs. 6 mg/kg oxaliplatin + 0.5 g/kg LEP-2 | -0.874 | 1.074 | 1.000 | 0.144 |
| 15 day | vehicle vs. 6 mg/kg oxaliplatin | -5.642 | -4.057 | < 0.001 | 6.278 |
|  | vehicle vs. 2 g/kg LEM | -0.942 | 0.642 | 1.000 | 0.259 |
|  | vehicle vs. 1 g/kg LEP | -0.592 | 0.992 | 0.998 | 0.320 |
|  | vehicle vs. 0.5 g/kg LEP-1 | -0.992 | 0.592 | 0.998 | 0.349 |
|  | vehicle vs. 0.5 g/kg LEP-2 | -0.107 | 1.592 | 0.146 | 0.899 |
|  | vehicle vs. 6 mg/kg oxaliplatin + 2 g/kg LEM | -0.442 | 1.142 | 0.913 | 0.495 |
|  | vehicle vs. 6 mg/kg oxaliplatin + 1 g/kg LEP | -0.692 | 0.892 | 1.000 | 0.135 |
|  | vehicle vs. 6 mg/kg oxaliplatin + 0.5 g/kg LEP-1 | -5.642 | -4.057 | < 0.001 | 6.030 |
|  | vehicle vs. 6 mg/kg oxaliplatin + 0.5 g/kg LEP-2 | -0.942 | 0.642 | 1.000 | 0.217 |

Supporting Table 8: Effect sizes and 95% confident interval for significant results in Figure 2d.

| Days | Comparison | 95% confidence intervals | | P value | *d* |
| --- | --- | --- | --- | --- | --- |
|  |  | Lower limit | Upper limit |  |  |
| 0 day | vehicle vs. 6 mg/kg oxaliplatin | -0.990 | 0.690 | 1.000 | 0.240 |
|  | vehicle vs. 2 g/kg LEM | -1.140 | 0.540 | 0.977 | 0.456 |
|  | vehicle vs. 1 g/kg LEP | -0.890 | 0.790 | 1.000 | 0.085 |
|  | vehicle vs. 0.5 g/kg LEP-1 | -0.840 | 0.840 | 1.000 | 0 |
|  | vehicle vs. 0.5 g/kg LEP-2 | -0.990 | 0.690 | 1.000 | 0.226 |
|  | vehicle vs. 6 mg/kg oxaliplatin + 2 g/kg LEM | -1.390 | 0.290 | 0.517 | 0.821 |
|  | vehicle vs. 6 mg/kg oxaliplatin + 1 g/kg LEP | -0.740 | 0.940 | 1.000 | 0.164 |
|  | vehicle vs. 6 mg/kg oxaliplatin + 0.5 g/kg LEP-1 | -0.890 | 0.790 | 1.000 | 0.063 |
|  | vehicle vs. 6 mg/kg oxaliplatin + 0.5 g/kg LEP-2 | -0.890 | 0.790 | 1.000 | 0.074 |
| 3 day | vehicle vs. 6 mg/kg oxaliplatin | -5.219 | -2.680 | < 0.001 | 4.254 |
|  | vehicle vs. 2 g/kg LEM | -2.369 | 0.169 | 0.148 | 1.157 |
|  | vehicle vs. 1 g/kg LEP | -1.769 | 0.769 | 0.956 | 0.493 |
|  | vehicle vs. 0.5 g/kg LEP-1 | -2.069 | 0.469 | 0.570 | 0.763 |
|  | vehicle vs. 0.5 g/kg LEP-2 | -1.519 | 1.019 | 1.000 | 0.250 |
|  | vehicle vs. 6 mg/kg oxaliplatin + 2 g/kg LEM | -2.069 | 0.469 | 0.570 | 0.796 |
|  | vehicle vs. 6 mg/kg oxaliplatin + 1 g/kg LEP | -1.669 | 0.869 | 0.990 | 0.460 |
|  | vehicle vs. 6 mg/kg oxaliplatin + 0.5 g/kg LEP-1 | -5.669 | -3.130 | < 0.001 | 4.037 |
|  | vehicle vs. 6 mg/kg oxaliplatin + 0.5 g/kg LEP-2 | -1.569 | 0.969 | 0.999 | 0.294 |
| 6 day | vehicle vs. 6 mg/kg oxaliplatin | -5.162 | -3.437 | < 0.001 | 6.278 |
|  | vehicle vs. 2 g/kg LEM | -0.962 | 0.762 | 1.000 | 0.146 |
|  | vehicle vs. 1 g/kg LEP | -0.562 | 1.162 | 0.980 | 0.424 |
|  | vehicle vs. 0.5 g/kg LEP-1 | -0.862 | 0.862 | 1.000 | 0 |
|  | vehicle vs. 0.5 g/kg LEP-2 | -0.337 | 2.062 | 0.501 | 0.866 |
|  | vehicle vs. 6 mg/kg oxaliplatin + 2 g/kg LEM | -0.762 | 0.962 | 1.000 | 0.171 |
|  | vehicle vs. 6 mg/kg oxaliplatin + 1 g/kg LEP | -0.062 | 1.662 | 0.093 | 1.197 |
|  | vehicle vs. 6 mg/kg oxaliplatin + 0.5 g/kg LEP-1 | -4.662 | -2.937 | < 0.001 | 4.662 |
|  | vehicle vs. 6 mg/kg oxaliplatin + 0.5 g/kg LEP-2 | -1.162 | 0.562 | 0.980 | 0.400 |
| 9 day | vehicle vs. 6 mg/kg oxaliplatin | -5.108 | -2.691 | < 0.001 | 5.236 |
|  | vehicle vs. 2 g/kg LEM | -1.208 | 1.208 | 1.000 | 0 |
|  | vehicle vs. 1 g/kg LEP | -0.608 | 1.808 | 0.839 | 0.780 |
|  | vehicle vs. 0.5 g/kg LEP-1 | -1.408 | 1.008 | 1.000 | 0.237 |
|  | vehicle vs. 0.5 g/kg LEP-2 | -0.908 | 1.508 | 0.998 | 0.545 |
|  | vehicle vs. 6 mg/kg oxaliplatin + 2 g/kg LEM | -1.108 | 1.308 | 1.000 | 0.123 |
|  | vehicle vs. 6 mg/kg oxaliplatin + 1 g/kg LEP | -1.308 | 1.108 | 1.000 | 0.125 |
|  | vehicle vs. 6 mg/kg oxaliplatin + 0.5 g/kg LEP-1 | -5.108 | -2.691 | < 0.001 | 4.534 |
|  | vehicle vs. 6 mg/kg oxaliplatin + 0.5 g/kg LEP-2 | -1.408 | 1.008 | 1.000 | 0.216 |
| 12 day | vehicle vs. 6 mg/kg oxaliplatin | -5.555 | -3.344 | < 0.001 | 5.607 |
|  | vehicle vs. 2 g/kg LEM | -1.455 | 0.755 | 0.990 | 0.550 |
|  | vehicle vs. 1 g/kg LEP | -1.455 | 0.755 | 0.990 | 0.625 |
|  | vehicle vs. 0.5 g/kg LEP-1 | -1.155 | 1.055 | 1.000 | 0.074 |
|  | vehicle vs. 0.5 g/kg LEP-2 | -0.605 | 1.605 | 0.901 | 0.983 |
|  | vehicle vs. 6 mg/kg oxaliplatin + 2 g/kg LEM | -1.855 | 0.355 | 0.464 | 1.179 |
|  | vehicle vs. 6 mg/kg oxaliplatin + 1 g/kg LEP | -0.955 | 1.255 | 1.000 | 0.182 |
|  | vehicle vs. 6 mg/kg oxaliplatin + 0.5 g/kg LEP-1 | -6.305 | -4.094 | < 0.001 | 5.876 |
|  | vehicle vs. 6 mg/kg oxaliplatin + 0.5 g/kg LEP-2 | -1.605 | 0.605 | 0.901 | 0.691 |
| 15 day | vehicle vs. 6 mg/kg oxaliplatin | -6.572 | -4.327 | < 0.001 | 7.087 |
|  | vehicle vs. 2 g/kg LEM | -1.522 | 0.722 | 0.977 | 0.795 |
|  | vehicle vs. 1 g/kg LEP | -1.122 | 1.122 | 1.000 | 0 |
|  | vehicle vs. 0.5 g/kg LEP-1 | -1.422 | 0.822 | 0.997 | 0.506 |
|  | vehicle vs. 0.5 g/kg LEP-2 | -1.122 | 1.122 | 1.000 | 0 |
|  | vehicle vs. 6 mg/kg oxaliplatin + 2 g/kg LEM | -1.622 | 0.622 | 0.909 | 0.816 |
|  | vehicle vs. 6 mg/kg oxaliplatin + 1 g/kg LEP | -0.622 | 1.622 | 0.909 | 0.562 |
|  | vehicle vs. 6 mg/kg oxaliplatin + 0.5 g/kg LEP-1 | -6.372 | -4.127 | < 0.001 | 5.801 |
|  | vehicle vs. 6 mg/kg oxaliplatin + 0.5 g/kg LEP-2 | -0.772 | 1.472 | 0.991 | 0.457 |

Supporting Table 9: Effect sizes and 95% confident interval for significant results in Figure 2e.

| Days | Comparison | 95% confidence intervals | | P value | *d* |
| --- | --- | --- | --- | --- | --- |
|  |  | Lower limit | Upper limit |  |  |
| 0 day | vehicle vs. 6 mg/kg oxaliplatin | -1.283 | 1.283 | 1.000 | 0 |
|  | vehicle vs. 2 g/kg LEM | -1.483 | 1.083 | 1.000 | 0.201 |
|  | vehicle vs. 1 g/kg LEP | -1.583 | 0.983 | 0.999 | 0.332 |
|  | vehicle vs. 0.5 g/kg LEP-1 | -0.883 | 1.683 | 0.991 | 0.384 |
|  | vehicle vs. 0.5 g/kg LEP-2 | -1.5833 | 0.983 | 0.999 | 0.362 |
|  | vehicle vs. 6 mg/kg oxaliplatin + 2 g/kg LEM | -1.883 | 0.683 | 0.882 | 0.758 |
|  | vehicle vs. 6 mg/kg oxaliplatin + 1 g/kg LEP | -1.783 | 0.783 | 0.959 | 0.540 |
|  | vehicle vs. 6 mg/kg oxaliplatin + 0.5 g/kg LEP-1 | -1.383 | 1.183 | 1.000 | 0.104 |
|  | vehicle vs. 6 mg/kg oxaliplatin + 0.5 g/kg LEP-2 | -1.683 | 0.883 | 0.991 | 0.432 |
| 3 day | vehicle vs. 6 mg/kg oxaliplatin | -4.218 | -1.681 | < 0.001 | 5.180 |
|  | vehicle vs. 2 g/kg LEM | -1.618 | 0.918 | 0.996 | 0.389 |
|  | vehicle vs. 1 g/kg LEP | -1.968 | 0.568 | 0.739 | 1.445 |
|  | vehicle vs. 0.5 g/kg LEP-1 | -1.368 | 1.168 | 1.000 | 0.158 |
|  | vehicle vs. 0.5 g/kg LEP-2 | -0.818 | 1.718 | 0.978 | 0.554 |
|  | vehicle vs. 6 mg/kg oxaliplatin + 2 g/kg LEM | -0.868 | 1.668 | 0.990 | 0.496 |
|  | vehicle vs. 6 mg/kg oxaliplatin + 1 g/kg LEP | -1.468 | 1.068 | 1.000 | 0.355 |
|  | vehicle vs. 6 mg/kg oxaliplatin + 0.5 g/kg LEP-1 | -4.168 | -1.631 | < 0.001 | 3.727 |
|  | vehicle vs. 6 mg/kg oxaliplatin + 0.5 g/kg LEP-2 | -1.668 | 0.868 | 0.990 | 0.475 |
| 6 day | vehicle vs. 6 mg/kg oxaliplatin | -6.051 | -3.848 | < 0.001 | 5.611 |
|  | vehicle vs. 2 g/kg LEM | -1.301 | 0.901 | 1.000 | 0.213 |
|  | vehicle vs. 1 g/kg LEP | -1.601 | 0.601 | 0.899 | 0.725 |
|  | vehicle vs. 0.5 g/kg LEP-1 | -1.401 | 0.801 | 0.997 | 0.383 |
|  | vehicle vs. 0.5 g/kg LEP-2 | -0.501 | 1.701 | 0.753 | 0.737 |
|  | vehicle vs. 6 mg/kg oxaliplatin + 2 g/kg LEM | -1.301 | 0.901 | 1.000 | 0.265 |
|  | vehicle vs. 6 mg/kg oxaliplatin + 1 g/kg LEP | -1.051 | 1.151 | 1.000 | 0.065 |
|  | vehicle vs. 6 mg/kg oxaliplatin + 0.5 g/kg LEP-1 | -5.701 | -3.498 | < 0.001 | 5.111 |
|  | vehicle vs. 6 mg/kg oxaliplatin + 0.5 g/kg LEP-2 | -1.201 | 1.001 | 1.000 | 0.098 |
| 9 day | vehicle vs. 6 mg/kg oxaliplatin | -6.097 | -3.602 | < 0.001 | 5.686 |
|  | vehicle vs. 2 g/kg LEM | -0.897 | 1.597 | 0.996 | 0.395 |
|  | vehicle vs. 1 g/kg LEP | -1.497 | 0.997 | 1.000 | 0.331 |
|  | vehicle vs. 0.5 g/kg LEP-1 | -1.447 | 1.047 | 1.000 | 0.273 |
|  | vehicle vs. 0.5 g/kg LEP-2 | -0.397 | 2.097 | 0.458 | 1.138 |
|  | vehicle vs. 6 mg/kg oxaliplatin + 2 g/kg LEM | -1.547 | 0.947 | 0.999 | 0.389 |
|  | vehicle vs. 6 mg/kg oxaliplatin + 1 g/kg LEP | -1.697 | 0.797 | 0.975 | 0.558 |
|  | vehicle vs. 6 mg/kg oxaliplatin + 0.5 g/kg LEP-1 | -5.197 | -2.702 | < 0.001 | 3.885 |
|  | vehicle vs. 6 mg/kg oxaliplatin + 0.5 g/kg LEP-2 | -1.197 | 1.297 | 1.000 | 0.051 |
| 12 day | vehicle vs. 6 mg/kg oxaliplatin | -6.442 | -3.857 | < 0.001 | 5.341 |
|  | vehicle vs. 2 g/kg LEM | -1.692 | 0.892 | 0.991 | 0.479 |
|  | vehicle vs. 1 g/kg LEP | -1.342 | 1.242 | 1.000 | 0.058 |
|  | vehicle vs. 0.5 g/kg LEP-1 | -1.492 | 1.092 | 1.000 | 0.247 |
|  | vehicle vs. 0.5 g/kg LEP-2 | -0.442 | 2.142 | 0.509 | 0.994 |
|  | vehicle vs. 6 mg/kg oxaliplatin + 2 g/kg LEM | -1.792 | 0.792 | 0.961 | 0.624 |
|  | vehicle vs. 6 mg/kg oxaliplatin + 1 g/kg LEP | -1.442 | 1.142 | 1.000 | 0.147 |
|  | vehicle vs. 6 mg/kg oxaliplatin + 0.5 g/kg LEP-1 | -6.142 | -3.557 | < 0.001 | 5.319 |
|  | vehicle vs. 6 mg/kg oxaliplatin + 0.5 g/kg LEP-2 | -1.642 | 0.942 | 0.997 | 0.398 |
| 15 day | vehicle vs. 6 mg/kg oxaliplatin | -6.923 | -4.276 | < 0.001 | 6.996 |
|  | vehicle vs. 2 g/kg LEM | -1.523 | 1.123 | 1.000 | 0.255 |
|  | vehicle vs. 1 g/kg LEP | -1.523 | 1.123 | 1.000 | 0.221 |
|  | vehicle vs. 0.5 g/kg LEP-1 | -1.673 | 0.973 | 0.997 | 0.428 |
|  | vehicle vs. 0.5 g/kg LEP-2 | -0.623 | 2.023 | 0.784 | 0.922 |
|  | vehicle vs. 6 mg/kg oxaliplatin + 2 g/kg LEM | -1.523 | 1.123 | 1.000 | 0.236 |
|  | vehicle vs. 6 mg/kg oxaliplatin + 1 g/kg LEP | -0.723 | 1.923 | 0.900 | 0.604 |
|  | vehicle vs. 6 mg/kg oxaliplatin + 0.5 g/kg LEP-1 | -6.323 | -3.676 | < 0.001 | 5.527 |
|  | vehicle vs. 6 mg/kg oxaliplatin + 0.5 g/kg LEP-2 | -1.223 | 1.423 | 1.000 | 0.101 |

Supporting Table 10: Effect sizes and 95% confident interval for significant results in Figure 2b.

| Days | Comparison | 95% confidence intervals | | P value | *d* |
| --- | --- | --- | --- | --- | --- |
|  |  | Lower limit | Upper limit |  |  |
| 0 day | vehicle vs. 6 mg/kg oxaliplatin | -1.409 | 0.470 | 0.544 | 0.580 |
|  | vehicle vs. 2 g/kg LEM | -1.229 | 0.709 | 0.997 | 0.254 |
|  | vehicle vs. 1 g/kg LEP | -1.009 | 0.929 | 1.000 | 0.043 |
|  | vehicle vs. 0.5 g/kg LEP-1 | -1.199 | 0.739 | 0.999 | 0.277 |
|  | vehicle vs. 0.5 g/kg LEP-2 | -0.909 | 1.029 | 1.000 | 0.067 |
|  | vehicle vs. 6 mg/kg oxaliplatin + 2 g/kg LEM | -0.999 | 0.939 | 1.000 | 0.034 |
|  | vehicle vs. 6 mg/kg oxaliplatin + 1 g/kg LEP | -1.739 | 0.199 | 0.244 | 0.910 |
|  | vehicle vs. 6 mg/kg oxaliplatin + 0.5 g/kg LEP-1 | -1.739 | 0.199 | 0.244 | 0.906 |
|  | vehicle vs. 6 mg/kg oxaliplatin + 0.5 g/kg LEP-2 | -1.539 | 0.399 | 0.664 | 0.561 |
| 3 day | vehicle vs. 6 mg/kg oxaliplatin | -0.009 | 2.429 | 0.053 | 1.185 |
|  | vehicle vs. 2 g/kg LEM | -1.019 | 1.419 | 1.000 | 0.218 |
|  | vehicle vs. 1 g/kg LEP | -0.639 | 1.799 | 0.871 | 0.631 |
|  | vehicle vs. 0.5 g/kg LEP-1 | -1.139 | 1.299 | 1.000 | 0.089 |
|  | vehicle vs. 0.5 g/kg LEP-2 | -1.029 | 1.409 | 1.000 | 0.217 |
|  | vehicle vs. 6 mg/kg oxaliplatin + 2 g/kg LEM | -0.240 | 1.679 | 0.165 | 0.971 |
|  | vehicle vs. 6 mg/kg oxaliplatin + 1 g/kg LEP | -0.330 | 1.769 | 0.221 | 0.879 |
|  | vehicle vs. 6 mg/kg oxaliplatin + 0.5 g/kg LEP-1 | -1.000 | 1.439 | 0.287 | 0.972 |
|  | vehicle vs. 6 mg/kg oxaliplatin + 0.5 g/kg LEP-2 | -0.880 | 1.319 | 0.564 | 0.830 |
| 6 day | vehicle vs. 6 mg/kg oxaliplatin | -0.966 | 1.906 | 0.987 | 0.399 |
|  | vehicle vs. 2 g/kg LEM | -0.666 | 2.206 | 0.770 | 0.766 |
|  | vehicle vs. 1 g/kg LEP | -0.296 | 2.576 | 0.245 | 1.176 |
|  | vehicle vs. 0.5 g/kg LEP-1 | -1.026 | 1.846 | 0.995 | 0.413 |
|  | vehicle vs. 0.5 g/kg LEP-2 | -1.146 | 1.726 | 1.000 | 0.315 |
|  | vehicle vs. 6 mg/kg oxaliplatin + 2 g/kg LEM | -0.196 | 2.676 | 0.152 | 1.189 |
|  | vehicle vs. 6 mg/kg oxaliplatin + 1 g/kg LEP | -1.116 | 1.756 | 0.999 | 0.314 |
|  | vehicle vs. 6 mg/kg oxaliplatin + 0.5 g/kg LEP-1 | -0.113 | 2.886 | 0.106 | 1.147 |
|  | vehicle vs. 6 mg/kg oxaliplatin + 0.5 g/kg LEP-2 | -0.566 | 2.306 | 0.625 | 0.746 |
| 9 day | vehicle vs. 6 mg/kg oxaliplatin | -0.168 | 2.511 | 0.081 | 1.170 |
|  | vehicle vs. 2 g/kg LEM | -0.141 | 2.301 | 0.130 | 1.223 |
|  | vehicle vs. 1 g/kg LEP | -0.168 | 2.511 | 0.080 | 1.242 |
|  | vehicle vs. 0.5 g/kg LEP-1 | -0.541 | 1.901 | 0.729 | 0.657 |
|  | vehicle vs. 0.5 g/kg LEP-2 | -0.981 | 1.461 | 1.000 | 0.264 |
|  | vehicle vs. 6 mg/kg oxaliplatin + 2 g/kg LEM | -0.198 | 2.541 | 0.084 | 1.286 |
|  | vehicle vs. 6 mg/kg oxaliplatin + 1 g/kg LEP | -0.091 | 2.351 | 0.094 | 1.203 |
|  | vehicle vs. 6 mg/kg oxaliplatin + 0.5 g/kg LEP-1 | -0.928 | 3.371 | 0.095 | 1.372 |
|  | vehicle vs. 6 mg/kg oxaliplatin + 0.5 g/kg LEP-2 | -0.108 | 2.451 | 0.097 | 1.149 |
| 12 day | vehicle vs. 6 mg/kg oxaliplatin | -0.609 | 1.949 | 0.793 | 0.625 |
|  | vehicle vs. 2 g/kg LEM | -0.809 | 1.749 | 0.972 | 0.486 |
|  | vehicle vs. 1 g/kg LEP | -0.269 | 2.289 | 0.252 | 0.993 |
|  | vehicle vs. 0.5 g/kg LEP-1 | -1.389 | 1.169 | 1.000 | 0.099 |
|  | vehicle vs. 0.5 g/kg LEP-2 | -1.229 | 1.329 | 1.000 | 0.051 |
|  | vehicle vs. 6 mg/kg oxaliplatin + 2 g/kg LEM | -0.159 | 2.399 | 0.139 | 1.231 |
|  | vehicle vs. 6 mg/kg oxaliplatin + 1 g/kg LEP | -0.529 | 2.029 | 0.668 | 0.765 |
|  | vehicle vs. 6 mg/kg oxaliplatin + 0.5 g/kg LEP-1 | -0.066 | 3.219 | 0.091 | 1.181 |
|  | vehicle vs. 6 mg/kg oxaliplatin + 0.5 g/kg LEP-2 | -0.059 | 2.499 | 0.075 | 1.072 |
| 15 day | vehicle vs. 6 mg/kg oxaliplatin | -0.321 | 2.161 | 0.336 | 0.916 |
|  | vehicle vs. 2 g/kg LEM | -0.261 | 2.221 | 0.252 | 1.129 |
|  | vehicle vs. 1 g/kg LEP | -0.001 | 2.481 | 0.074 | 1.260 |
|  | vehicle vs. 0.5 g/kg LEP-1 | -0.791 | 1.691 | 0.974 | 0.445 |
|  | vehicle vs. 0.5 g/kg LEP-2 | -0.591 | 1.891 | 0.793 | 0.716 |
|  | vehicle vs. 6 mg/kg oxaliplatin + 2 g/kg LEM | -0.018 | 2.501 | 0.084 | 1.322 |
|  | vehicle vs. 6 mg/kg oxaliplatin + 1 g/kg LEP | -0.131 | 2.351 | 0.121 | 1.235 |
|  | vehicle vs. 6 mg/kg oxaliplatin + 0.5 g/kg LEP-1 | -0.068 | 2.121 | 0.091 | 1.293 |
|  | vehicle vs. 6 mg/kg oxaliplatin + 0.5 g/kg LEP-2 | -0.098 | 2.581 | 0.094 | 1.214 |

Supporting Table 11: Effect sizes and 95% confident interval for significant results in Figure 3a.

| Days | Comparison | 95% confidence intervals | | P value | *r* |
| --- | --- | --- | --- | --- | --- |
|  |  | Lower limit | Upper limit |  |  |
| 0 day | vehicle vs. 6 mg/kg oxaliplatin | -70.712 | 70.712 | 1.000 | 0 |
|  | vehicle vs. 2 g/kg LEM | -59.412 | 82.012 | 0.996 | 0.355 |
|  | vehicle vs. 0.5 g/kg LEP-2 | -70.712 | 70.712 | 1.000 | 0 |
|  | vehicle vs. 0.25 g/kg LEP-3 | -70.712 | 70.712 | 1.000 | 0 |
|  | vehicle vs. 0.25 g/kg LEP-4 | -70.712 | 70.712 | 1.000 | 0 |
|  | vehicle vs. 30 mg/kg Duloxetine | -52.412 | 89.012 | 0.828 | 0.576 |
|  | vehicle vs. 6 mg/kg oxaliplatin + 2 g/kg LEM | -59.012 | 82.412 | 0.994 | 0.368 |
|  | vehicle vs. 6 mg/kg oxaliplatin + 0.5 g/kg LEP-2 | -70.712 | 70.712 | 1.000 | 0 |
|  | vehicle vs. 6 mg/kg oxaliplatin + 0.25 g/kg LEP-3 | -70.712 | 70.712 | 1.000 | 0 |
|  | vehicle vs. 6 mg/kg oxaliplatin + 0.25 g/kg LEP-4 | -58.012 | 83.412 | 0.988 | 0.399 |
|  | vehicle vs. 6 mg/kg oxaliplatin + 30 mg/kg Duloxetine | -70.712 | 70.712 | 1.000 | 0 |
| 3 day | vehicle vs. 6 mg/kg oxaliplatin | 0.687 | 142.112 | 0.0016 | 1.223 |
|  | vehicle vs. 2 g/kg LEM | -53.462 | 87.962 | 0.999 | 0.295 |
|  | vehicle vs. 0.5 g/kg LEP-2 | -56.762 | 84.662 | 1.000 | 0.239 |
|  | vehicle vs. 0.25 g/kg LEP-3 | -70.712 | 70.712 | 1.000 | 0 |
|  | vehicle vs. 0.25 g/kg LEP-4 | -54.962 | 86.462 | 1.000 | 0.269 |
|  | vehicle vs. 30 mg/kg Duloxetine | -40.112 | 101.312 | 0.905 | 0.524 |
|  | vehicle vs. 6 mg/kg oxaliplatin + 2 g/kg LEM | -44.862 | 96.562 | 0.972 | 0.442 |
|  | vehicle vs. 6 mg/kg oxaliplatin + 0.5 g/kg LEP-2 | -70.712 | 70.712 | 1.000 | 0 |
|  | vehicle vs. 6 mg/kg oxaliplatin + 0.25 g/kg LEP-3 | -24.562 | 116.862 | 0.327 | 0.790 |
|  | vehicle vs. 6 mg/kg oxaliplatin + 0.25 g/kg LEP-4 | -70.712 | 70.712 | 1.000 | 0 |
|  | vehicle vs. 6 mg/kg oxaliplatin + 30 mg/kg Duloxetine | -51.662 | 89.762 | 0.998 | 0.326 |
| 6 day | vehicle vs. 6 mg/kg oxaliplatin | -4.112 | 137.312 | < 0.001 | 1.370 |
|  | vehicle vs. 2 g/kg LEM | -70.712 | 70.712 | 1.000 | 0 |
|  | vehicle vs. 0.5 g/kg LEP-2 | -59.512 | 81.912 | 1.000 | 0.230 |
|  | vehicle vs. 0.25 g/kg LEP-3 | -70.712 | 70.712 | 1.000 | 0 |
|  | vehicle vs. 0.25 g/kg LEP-4 | -49.512 | 91.912 | 0.975 | 0.436 |
|  | vehicle vs. 30 mg/kg Duloxetine | -70.712 | 70.712 | 1.000 | 0 |
|  | vehicle vs. 6 mg/kg oxaliplatin + 2 g/kg LEM | -70.712 | 70.712 | 1.000 | 0 |
|  | vehicle vs. 6 mg/kg oxaliplatin + 0.5 g/kg LEP-2 | -70.712 | 70.712 | 1.000 | 0 |
|  | vehicle vs. 6 mg/kg oxaliplatin + 0.25 g/kg LEP-3 | -20.562 | 110.862 | 0.005 | 1.120 |
|  | vehicle vs. 6 mg/kg oxaliplatin + 0.25 g/kg LEP-4 | -55.862 | 85.562 | 0.999 | 0.305 |
|  | vehicle vs. 6 mg/kg oxaliplatin + 30 mg/kg Duloxetine | -70.712 | 70.712 | 1.000 | 0 |
| 9 day | vehicle vs. 6 mg/kg oxaliplatin | -3.712 | 137.712 | < 0.001 | 1.378 |
|  | vehicle vs. 2 g/kg LEM | -70.712 | 70.712 | 1.000 | 0 |
|  | vehicle vs. 0.5 g/kg LEP-2 | -70.712 | 70.712 | 1.000 | 0 |
|  | vehicle vs. 0.25 g/kg LEP-3 | -70.712 | 70.712 | 1.000 | 0 |
|  | vehicle vs. 0.25 g/kg LEP-4 | -60.912 | 80.512 | 1.000 | 0.201 |
|  | vehicle vs. 30 mg/kg Duloxetine | -70.712 | 70.712 | 1.000 | 0 |
|  | vehicle vs. 6 mg/kg oxaliplatin + 2 g/kg LEM | -60.512 | 80.912 | 1.000 | 0.209 |
|  | vehicle vs. 6 mg/kg oxaliplatin + 0.5 g/kg LEP-2 | -70.712 | 70.712 | 1.000 | 0 |
|  | vehicle vs. 6 mg/kg oxaliplatin + 0.25 g/kg LEP-3 | -13.712 | 127.712 | 0.004 | 1.172 |
|  | vehicle vs. 6 mg/kg oxaliplatin + 0.25 g/kg LEP-4 | -70.712 | 70.712 | 1.000 | 0 |
|  | vehicle vs. 6 mg/kg oxaliplatin + 30 mg/kg Duloxetine | -70.712 | 70.712 | 1.000 | 0 |
| 12 day | vehicle vs. 6 mg/kg oxaliplatin | -4.912 | 136.512 | < 0.001 | 1.354 |
|  | vehicle vs. 2 g/kg LEM | -70.712 | 70.712 | 1.000 | 0 |
|  | vehicle vs. 0.5 g/kg LEP-2 | -70.712 | 70.712 | 1.000 | 0 |
|  | vehicle vs. 0.25 g/kg LEP-3 | -70.712 | 70.712 | 1.000 | 0 |
|  | vehicle vs. 0.25 g/kg LEP-4 | -60.912 | 80.512 | 1.000 | 0.201 |
|  | vehicle vs. 30 mg/kg Duloxetine | -70.712 | 70.712 | 1.000 | 0 |
|  | vehicle vs. 6 mg/kg oxaliplatin + 2 g/kg LEM | -60.512 | 80.912 | 1.000 | 0.209 |
|  | vehicle vs. 6 mg/kg oxaliplatin + 0.5 g/kg LEP-2 | -70.712 | 70.712 | 1.000 | 0 |
|  | vehicle vs. 6 mg/kg oxaliplatin + 0.25 g/kg LEP-3 | -12.512 | 128.912 | 0.003 | 1.197 |
|  | vehicle vs. 6 mg/kg oxaliplatin + 0.25 g/kg LEP-4 | -70.712 | 70.712 | 1.000 | 0 |
|  | vehicle vs. 6 mg/kg oxaliplatin + 30 mg/kg Duloxetine | -70.712 | 70.712 | 1.000 | 0 |
| 15 day | vehicle vs. 6 mg/kg oxaliplatin | -1.212 | 140.212 | < 0.001 | 1.269 |
|  | vehicle vs. 2 g/kg LEM | -70.712 | 70.712 | 1.000 | 0 |
|  | vehicle vs. 0.5 g/kg LEP-2 | -70.712 | 70.712 | 1.000 | 0 |
|  | vehicle vs. 0.25 g/kg LEP-3 | -70.712 | 70.712 | 1.000 | 0 |
|  | vehicle vs. 0.25 g/kg LEP-4 | -61.812 | 79.612 | 1.000 | 0.162 |
|  | vehicle vs. 30 mg/kg Duloxetine | -39.812 | 101.612 | 0.847 | 0.564 |
|  | vehicle vs. 6 mg/kg oxaliplatin + 2 g/kg LEM | -59.612 | 81.812 | 1.000 | 0.202 |
|  | vehicle vs. 6 mg/kg oxaliplatin + 0.5 g/kg LEP-2 | -70.712 | 70.712 | 1.000 | 0 |
|  | vehicle vs. 6 mg/kg oxaliplatin + 0.25 g/kg LEP-3 | -7.2125 | 134.212 | 0.005 | 1.160 |
|  | vehicle vs. 6 mg/kg oxaliplatin + 0.25 g/kg LEP-4 | -70.712 | 70.712 | 1.000 | 0 |
|  | vehicle vs. 6 mg/kg oxaliplatin + 30 mg/kg Duloxetine | -56.612 | 84.812 | 1.000 | 0.257 |

Supporting Table 12: Effect sizes and 95% confident interval for significant results in Figure 3c.

| Days | Comparison | 95% confidence intervals | | P value | *d* |
| --- | --- | --- | --- | --- | --- |
|  |  | Lower limit | Upper limit |  |  |
| 0 day | vehicle vs. 6 mg/kg oxaliplatin | -1.787 | 0.212 | 0.413 | 0.712 |
|  | vehicle vs. 2 g/kg LEM | -1.187 | 0.387 | 0.866 | 0.477 |
|  | vehicle vs. 0.5 g/kg LEP-2 | -0.987 | 0.587 | 0.999 | 0.281 |
|  | vehicle vs. 0.25 g/kg LEP-3 | -0.587 | 0.987 | 0.999 | 0.277 |
|  | vehicle vs. 0.25 g/kg LEP-4 | -0.987 | 0.587 | 0.999 | 0.255 |
|  | vehicle vs. 30 mg/kg Duloxetine | -1.387 | 0.187 | 0.324 | 0.814 |
|  | vehicle vs. 6 mg/kg oxaliplatin + 2 g/kg LEM | -1.087 | 0.487 | 0.981 | 0.454 |
|  | vehicle vs. 6 mg/kg oxaliplatin + 0.5 g/kg LEP-2 | -1.087 | 0.487 | 0.981 | 0.416 |
|  | vehicle vs. 6 mg/kg oxaliplatin + 0.25 g/kg LEP-3 | -1.0875 | 0.487 | 0.981 | 0.416 |
|  | vehicle vs. 6 mg/kg oxaliplatin + 0.25 g/kg LEP-4 | -0.987 | 0.587 | 0.999 | 0.311 |
|  | vehicle vs. 6 mg/kg oxaliplatin + 30 mg/kg Duloxetine | -0.887 | 0.687 | 1.000 | 0.135 |
| 3 day | vehicle vs. 6 mg/kg oxaliplatin | -4.340 | -2.460 | < 0.001 | 5.831 |
|  | vehicle vs. 2 g/kg LEM | -1.040 | 0.840 | 1.000 | 0.175 |
|  | vehicle vs. 0.5 g/kg LEP-2 | -0.440 | 1.440 | 0.827 | 0.636 |
|  | vehicle vs. 0.25 g/kg LEP-3 | -0.840 | 1.040 | 1.000 | 0.149 |
|  | vehicle vs. 0.25 g/kg LEP-4 | -1.040 | 0.840 | 1.000 | 0.151 |
|  | vehicle vs. 30 mg/kg Duloxetine | -1.440 | 0.440 | 0.827 | 0.792 |
|  | vehicle vs. 6 mg/kg oxaliplatin + 2 g/kg LEM | -0.940 | 0.940 | 1.000 | 0 |
|  | vehicle vs. 6 mg/kg oxaliplatin + 0.5 g/kg LEP-2 | -1.140 | 0.740 | 1.000 | 0.242 |
|  | vehicle vs. 6 mg/kg oxaliplatin + 0.25 g/kg LEP-3 | -3.040 | -1.160 | < 0.001 | 3.374 |
|  | vehicle vs. 6 mg/kg oxaliplatin + 0.25 g/kg LEP-4 | -1.190 | 0.690 | 0.999 | 0.393 |
|  | vehicle vs. 6 mg/kg oxaliplatin + 30 mg/kg Duloxetine | -1.090 | 0.790 | 1.000 | 0.267 |
| 6 day | vehicle vs. 6 mg/kg oxaliplatin | -5.568 | -3.531 | < 0.001 | 7.152 |
|  | vehicle vs. 2 g/kg LEM | -1.418 | 0.618 | 0.976 | 0.711 |
|  | vehicle vs. 0.5 g/kg LEP-2 | -0.918 | 1.118 | 1.000 | 0.129 |
|  | vehicle vs. 0.25 g/kg LEP-3 | -1.018 | 1.018 | 1.000 | 0 |
|  | vehicle vs. 0.25 g/kg LEP-4 | -1.118 | 0.918 | 1.000 | 0.139 |
|  | vehicle vs. 30 mg/kg Duloxetine | -1.518 | 0.518 | 0.890 | 1.088 |
|  | vehicle vs. 6 mg/kg oxaliplatin + 2 g/kg LEM | -1.518 | 0.518 | 0.890 | 0.582 |
|  | vehicle vs. 6 mg/kg oxaliplatin + 0.5 g/kg LEP-2 | -1.018 | 1.018 | 1.000 | 0 |
|  | vehicle vs. 6 mg/kg oxaliplatin + 0.25 g/kg LEP-3 | -4.418 | -2.381 | < 0.001 | 6.046 |
|  | vehicle vs. 6 mg/kg oxaliplatin + 0.25 g/kg LEP-4 | -1.318 | 0.718 | 0.998 | 0.410 |
|  | vehicle vs. 6 mg/kg oxaliplatin + 30 mg/kg Duloxetine | -1.418 | 0.618 | 0.976 | 0.616 |
| 9 day | vehicle vs. 6 mg/kg oxaliplatin | -5.678 | -3.621 | < 0.001 | 5.281 |
|  | vehicle vs. 2 g/kg LEM | -0.928 | 1.128 | 1.000 | 0.153 |
|  | vehicle vs. 0.5 g/kg LEP-2 | -0.928 | 1.128 | 1.000 | 0.117 |
|  | vehicle vs. 0.25 g/kg LEP-3 | -0.628 | 1.428 | 0.978 | 0.584 |
|  | vehicle vs. 0.25 g/kg LEP-4 | -0.928 | 1.128 | 1.000 | 0.153 |
|  | vehicle vs. 30 mg/kg Duloxetine | -1.228 | 0.828 | 1.000 | 0.321 |
|  | vehicle vs. 6 mg/kg oxaliplatin + 2 g/kg LEM | -1.228 | 0.828 | 1.000 | 0.254 |
|  | vehicle vs. 6 mg/kg oxaliplatin + 0.5 g/kg LEP-2 | -0.428 | 1.628 | 0.725 | 0.717 |
|  | vehicle vs. 6 mg/kg oxaliplatin + 0.25 g/kg LEP-3 | -4.928 | -2.871 | < 0.001 | 4.664 |
|  | vehicle vs. 6 mg/kg oxaliplatin + 0.25 g/kg LEP-4 | -1.228 | 0.828 | 1.000 | 0.254 |
|  | vehicle vs. 6 mg/kg oxaliplatin + 30 mg/kg Duloxetine | -1.478 | 0.578 | 0.947 | 0.590 |
| 12 day | vehicle vs. 6 mg/kg oxaliplatin | -5.678 | -3.621 | < 0.001 | 5.281 |
|  | vehicle vs. 2 g/kg LEM | -0.928 | 1.128 | 1.000 | 0.153 |
|  | vehicle vs. 0.5 g/kg LEP-2 | -0.928 | 1.128 | 1.000 | 0.117 |
|  | vehicle vs. 0.25 g/kg LEP-3 | -0.628 | 1.428 | 0.978 | 0.584 |
|  | vehicle vs. 0.25 g/kg LEP-4 | -0.928 | 1.128 | 1.000 | 0.153 |
|  | vehicle vs. 30 mg/kg Duloxetine | -1.228 | 0.828 | 1.000 | 0.321 |
|  | vehicle vs. 6 mg/kg oxaliplatin + 2 g/kg LEM | -1.228 | 0.828 | 1.000 | 0.254 |
|  | vehicle vs. 6 mg/kg oxaliplatin + 0.5 g/kg LEP-2 | -0.428 | 1.628 | 0.725 | 0.717 |
|  | vehicle vs. 6 mg/kg oxaliplatin + 0.25 g/kg LEP-3 | -4.928 | -2.871 | < 0.001 | 4.664 |
|  | vehicle vs. 6 mg/kg oxaliplatin + 0.25 g/kg LEP-4 | -1.228 | 0.828 | 1.000 | 0.254 |
|  | vehicle vs. 6 mg/kg oxaliplatin + 30 mg/kg Duloxetine | -1.478 | 0.578 | 0.947 | 0.590 |
| 15 day | vehicle vs. 6 mg/kg oxaliplatin | -5.678 | -3.621 | < 0.001 | 5.281 |
|  | vehicle vs. 2 g/kg LEM | -0.928 | 1.128 | 1.000 | 0.153 |
|  | vehicle vs. 0.5 g/kg LEP-2 | -0.928 | 1.128 | 1.000 | 0.117 |
|  | vehicle vs. 0.25 g/kg LEP-3 | -0.628 | 1.428 | 0.978 | 0.584 |
|  | vehicle vs. 0.25 g/kg LEP-4 | -0.928 | 1.128 | 1.000 | 0.153 |
|  | vehicle vs. 30 mg/kg Duloxetine | -1.228 | 0.828 | 1.000 | 0.321 |
|  | vehicle vs. 6 mg/kg oxaliplatin + 2 g/kg LEM | -1.228 | 0.828 | 1.000 | 0.254 |
|  | vehicle vs. 6 mg/kg oxaliplatin + 0.5 g/kg LEP-2 | -0.428 | 1.628 | 0.725 | 0.717 |
|  | vehicle vs. 6 mg/kg oxaliplatin + 0.25 g/kg LEP-3 | -4.928 | -2.871 | < 0.001 | 4.664 |
|  | vehicle vs. 6 mg/kg oxaliplatin + 0.25 g/kg LEP-4 | -1.228 | 0.828 | 1.000 | 0.254 |
|  | vehicle vs. 6 mg/kg oxaliplatin + 30 mg/kg Duloxetine | -1.478 | 0.578 | 0.947 | 0.590 |

Supporting Table 13: Effect sizes and 95% confident interval for significant results in Figure 3d.

| Days | Comparison | 95% confidence intervals | | P value | *d* |
| --- | --- | --- | --- | --- | --- |
|  |  | Lower limit | Upper limit |  |  |
| 0 day | vehicle vs. 6 mg/kg oxaliplatin | -0.971 | 0.371 | 0.939 | 0.619 |
|  | vehicle vs. 2 g/kg LEM | -0.871 | 0.471 | 0.998 | 0.556 |
|  | vehicle vs. 0.5 g/kg LEP-2 | -0.571 | 0.771 | 1.000 | 0.149 |
|  | vehicle vs. 0.25 g/kg LEP-3 | -0.571 | 0.771 | 1.000 | 0.149 |
|  | vehicle vs. 0.25 g/kg LEP-4 | -0.571 | 0.771 | 1.000 | 0.196 |
|  | vehicle vs. 30 mg/kg Duloxetine | -0.771 | 0.571 | 1.000 | 0.206 |
|  | vehicle vs. 6 mg/kg oxaliplatin + 2 g/kg LEM | -0.871 | 0.471 | 0.998 | 0.367 |
|  | vehicle vs. 6 mg/kg oxaliplatin + 0.5 g/kg LEP-2 | -0.971 | 0.371 | 0.939 | 0.454 |
|  | vehicle vs. 6 mg/kg oxaliplatin + 0.25 g/kg LEP-3 | -0.771 | 0.571 | 1.000 | 0.175 |
|  | vehicle vs. 6 mg/kg oxaliplatin + 0.25 g/kg LEP-4 | -0.571 | 0.771 | 1.000 | 0.149 |
|  | vehicle vs. 6 mg/kg oxaliplatin + 30 mg/kg Duloxetine | -0.971 | 0.371 | 0.939 | 0.619 |
| 3 day | vehicle vs. 6 mg/kg oxaliplatin | -3.330 | -0.869 | < 0.001 | 2.892 |
|  | vehicle vs. 2 g/kg LEM | -0.530 | 1.930 | 0.756 | 1.244 |
|  | vehicle vs. 0.5 g/kg LEP-2 | -0.730 | 1.730 | 0.969 | 0.487 |
|  | vehicle vs. 0.25 g/kg LEP-3 | -1.230 | 1.230 | 1.000 | 0 |
|  | vehicle vs. 0.25 g/kg LEP-4 | -1.330 | 1.130 | 1.000 | 0.125 |
|  | vehicle vs. 30 mg/kg Duloxetine | -1.330 | 1.130 | 1.000 | 0.125 |
|  | vehicle vs. 6 mg/kg oxaliplatin + 2 g/kg LEM | -0.930 | 1.530 | 1.000 | 0.418 |
|  | vehicle vs. 6 mg/kg oxaliplatin + 0.5 g/kg LEP-2 | -0.030 | 2.430 | 0.063 | 1.175 |
|  | vehicle vs. 6 mg/kg oxaliplatin + 0.25 g/kg LEP-3 | -2.830 | -0.369 | 0.002 | 2.203 |
|  | vehicle vs. 6 mg/kg oxaliplatin + 0.25 g/kg LEP-4 | -1.130 | 1.330 | 1.000 | 0.126 |
|  | vehicle vs. 6 mg/kg oxaliplatin + 30 mg/kg Duloxetine | -1.480 | 0.980 | 1.000 | 0.332 |
| 6 day | vehicle vs. 6 mg/kg oxaliplatin | -4.874 | -2.025 | < 0.001 | 3.434 |
|  | vehicle vs. 2 g/kg LEM | -1.524 | 1.324 | 1.000 | 0.116 |
|  | vehicle vs. 0.5 g/kg LEP-2 | -0.924 | 1.924 | 0.990 | 0.454 |
|  | vehicle vs. 0.25 g/kg LEP-3 | -1.674 | 1.174 | 1.000 | 0.259 |
|  | vehicle vs. 0.25 g/kg LEP-4 | -1.624 | 1.224 | 1.000 | 0.227 |
|  | vehicle vs. 30 mg/kg Duloxetine | -1.324 | 1.524 | 1.000 | 0.121 |
|  | vehicle vs. 6 mg/kg oxaliplatin + 2 g/kg LEM | -1.824 | 1.024 | 0.999 | 0.415 |
|  | vehicle vs. 6 mg/kg oxaliplatin + 0.5 g/kg LEP-2 | -1.574 | 1.274 | 1.000 | 0.159 |
|  | vehicle vs. 6 mg/kg oxaliplatin + 0.25 g/kg LEP-3 | -4.474 | -1.625 | < 0.001 | 3.111 |
|  | vehicle vs. 6 mg/kg oxaliplatin + 0.25 g/kg LEP-4 | -1.224 | 1.624 | 1.000 | 0.180 |
|  | vehicle vs. 6 mg/kg oxaliplatin + 30 mg/kg Duloxetine | -2.274 | 0.574 | 0.696 | 1.058 |
| 9 day | vehicle vs. 6 mg/kg oxaliplatin | -5.159 | -2.740 | < 0.001 | 4.673 |
|  | vehicle vs. 2 g/kg LEM | -0.609 | 1.809 | 0.883 | 0.924 |
|  | vehicle vs. 0.5 g/kg LEP-2 | -0.909 | 1.509 | 1.000 | 0.309 |
|  | vehicle vs. 0.25 g/kg LEP-3 | -1.109 | 1.309 | 1.000 | 0.144 |
|  | vehicle vs. 0.25 g/kg LEP-4 | -0.909 | 1.509 | 1.000 | 0.448 |
|  | vehicle vs. 30 mg/kg Duloxetine | -0.709 | 1.709 | 0.965 | 0.857 |
|  | vehicle vs. 6 mg/kg oxaliplatin + 2 g/kg LEM | -1.509 | 0.909 | 1.000 | 0.387 |
|  | vehicle vs. 6 mg/kg oxaliplatin + 0.5 g/kg LEP-2 | -0.909 | 1.509 | 1.000 | 0.309 |
|  | vehicle vs. 6 mg/kg oxaliplatin + 0.25 g/kg LEP-3 | -4.709 | -2.290 | < 0.001 | 4.642 |
|  | vehicle vs. 6 mg/kg oxaliplatin + 0.25 g/kg LEP-4 | -1.209 | 1.209 | 1.000 | 0 |
|  | vehicle vs. 6 mg/kg oxaliplatin + 30 mg/kg Duloxetine | -1.609 | 0.809 | 0.994 | 0.616 |
| 12 day | vehicle vs. 6 mg/kg oxaliplatin | -5.124 | -2.775 | < 0.001 | 4.673 |
|  | vehicle vs. 2 g/kg LEM | -0.574 | 1.774 | 0.861 | 0.924 |
|  | vehicle vs. 0.5 g/kg LEP-2 | -0.874 | 1.474 | 0.999 | 0.309 |
|  | vehicle vs. 0.25 g/kg LEP-3 | -1.074 | 1.274 | 1.000 | 0.144 |
|  | vehicle vs. 0.25 g/kg LEP-4 | -0.874 | 1.474 | 0.999 | 0.448 |
|  | vehicle vs. 30 mg/kg Duloxetine | -0.674 | 1.674 | 0.957 | 0.857 |
|  | vehicle vs. 6 mg/kg oxaliplatin + 2 g/kg LEM | -1.474 | 0.874 | 0.999 | 0.387 |
|  | vehicle vs. 6 mg/kg oxaliplatin + 0.5 g/kg LEP-2 | -0.874 | 1.474 | 0.999 | 0.309 |
|  | vehicle vs. 6 mg/kg oxaliplatin + 0.25 g/kg LEP-3 | -5.074 | -2.725 | < 0.001 | 6.689 |
|  | vehicle vs. 6 mg/kg oxaliplatin + 0.25 g/kg LEP-4 | -1.174 | 1.174 | 1.000 | 0 |
|  | vehicle vs. 6 mg/kg oxaliplatin + 30 mg/kg Duloxetine | -1.574 | 0.774 | 0.992 | 0.616 |
| 15 day | vehicle vs. 6 mg/kg oxaliplatin | -5.124 | -2.775 | < 0.001 | 4.673 |
|  | vehicle vs. 2 g/kg LEM | -0.574 | 1.774 | 0.861 | 0.924 |
|  | vehicle vs. 0.5 g/kg LEP-2 | -0.874 | 1.474 | 0.999 | 0.309 |
|  | vehicle vs. 0.25 g/kg LEP-3 | -1.074 | 1.274 | 1.000 | 0.144 |
|  | vehicle vs. 0.25 g/kg LEP-4 | -0.874 | 1.474 | 0.999 | 0.448 |
|  | vehicle vs. 30 mg/kg Duloxetine | -0.674 | 1.674 | 0.957 | 0.857 |
|  | vehicle vs. 6 mg/kg oxaliplatin + 2 g/kg LEM | -1.474 | 0.874 | 0.999 | 0.387 |
|  | vehicle vs. 6 mg/kg oxaliplatin + 0.5 g/kg LEP-2 | -0.874 | 1.474 | 0.999 | 0.309 |
|  | vehicle vs. 6 mg/kg oxaliplatin + 0.25 g/kg LEP-3 | -5.124 | -2.775 | < 0.001 | 6.748 |
|  | vehicle vs. 6 mg/kg oxaliplatin + 0.25 g/kg LEP-4 | -1.174 | 1.174 | 1.000 | 0 |
|  | vehicle vs. 6 mg/kg oxaliplatin + 30 mg/kg Duloxetine | -1.574 | 0.774 | 0.992 | 0.616 |

Supporting Table 14: Effect sizes and 95% confident interval for significant results in Figure 3e.

| Days | Comparison | 95% confidence intervals | | P value | *d* |
| --- | --- | --- | --- | --- | --- |
|  |  | Lower limit | Upper limit |  |  |
| 0 day | vehicle vs. 6 mg/kg oxaliplatin | -0.815 | -0.815 | 1.000 | 0.063 |
|  | vehicle vs. 2 g/kg LEM | -0.665 | -0.665 | 1.000 | 0.158 |
|  | vehicle vs. 0.5 g/kg LEP-2 | -0.465 | -0.465 | 0.976 | 0.454 |
|  | vehicle vs. 0.25 g/kg LEP-3 | -0.265 | -0.265 | 0.565 | 0.531 |
|  | vehicle vs. 0.25 g/kg LEP-4 | -0.765 | -0.765 | 1.000 | 0 |
|  | vehicle vs. 30 mg/kg Duloxetine | -0.965 | -0.965 | 0.999 | 0.385 |
|  | vehicle vs. 6 mg/kg oxaliplatin + 2 g/kg LEM | -0.865 | -0.865 | 1.000 | 0.163 |
|  | vehicle vs. 6 mg/kg oxaliplatin + 0.5 g/kg LEP-2 | -0.365 | -0.365 | 0.842 | 0.585 |
|  | vehicle vs. 6 mg/kg oxaliplatin + 0.25 g/kg LEP-3 | -0.665 | -0.665 | 1.000 | 0.144 |
|  | vehicle vs. 6 mg/kg oxaliplatin + 0.25 g/kg LEP-4 | -0.965 | -0.965 | 0.999 | 0.303 |
|  | vehicle vs. 6 mg/kg oxaliplatin + 30 mg/kg Duloxetine | -0.965 | -0.965 | 0.999 | 0.303 |
| 3 day | vehicle vs. 6 mg/kg oxaliplatin | -3.462 | -0.937 | < 0.001 | 2.786 |
|  | vehicle vs. 2 g/kg LEM | -0.662 | 1.862 | 0.910 | 0.777 |
|  | vehicle vs. 0.5 g/kg LEP-2 | -0.662 | 1.862 | 0.910 | 0.565 |
|  | vehicle vs. 0.25 g/kg LEP-3 | -1.262 | 1.262 | 1.000 | 0 |
|  | vehicle vs. 0.25 g/kg LEP-4 | -1.662 | 0.862 | 0.996 | 0.542 |
|  | vehicle vs. 30 mg/kg Duloxetine | -1.762 | 0.762 | 0.974 | 0.703 |
|  | vehicle vs. 6 mg/kg oxaliplatin + 2 g/kg LEM | -1.262 | 1.262 | 1.000 | 0 |
|  | vehicle vs. 6 mg/kg oxaliplatin + 0.5 g/kg LEP-2 | -0.262 | 2.262 | 0.268 | 0.989 |
|  | vehicle vs. 6 mg/kg oxaliplatin + 0.25 g/kg LEP-3 | -2.562 | -0.372 | 0.008 | 1.728 |
|  | vehicle vs. 6 mg/kg oxaliplatin + 0.25 g/kg LEP-4 | -1.312 | 1.212 | 1.000 | 0.062 |
|  | vehicle vs. 6 mg/kg oxaliplatin + 30 mg/kg Duloxetine | -1.412 | 1.112 | 1.000 | 0.176 |
| 6 day | vehicle vs. 6 mg/kg oxaliplatin | -5.404 | -2.795 | < 0.001 | 4.853 |
|  | vehicle vs. 2 g/kg LEM | -1.404 | 1.204 | 1.000 | 0.118 |
|  | vehicle vs. 0.5 g/kg LEP-2 | -0.804 | 1.804 | 0.980 | 0.419 |
|  | vehicle vs. 0.25 g/kg LEP-3 | -1.204 | 1.404 | 1.000 | 0.097 |
|  | vehicle vs. 0.25 g/kg LEP-4 | -2.004 | 0.604 | 0.818 | 0.846 |
|  | vehicle vs. 30 mg/kg Duloxetine | -1.704 | 0.904 | 0.997 | 0.497 |
|  | vehicle vs. 6 mg/kg oxaliplatin + 2 g/kg LEM | -1.704 | 0.904 | 0.997 | 0.451 |
|  | vehicle vs. 6 mg/kg oxaliplatin + 0.5 g/kg LEP-2 | -1.104 | 1.504 | 1.000 | 0.186 |
|  | vehicle vs. 6 mg/kg oxaliplatin + 0.25 g/kg LEP-3 | -3.904 | -1.295 | < 0.001 | 2.954 |
|  | vehicle vs. 6 mg/kg oxaliplatin + 0.25 g/kg LEP-4 | -1.454 | 1.154 | 1.000 | 0.154 |
|  | vehicle vs. 6 mg/kg oxaliplatin + 30 mg/kg Duloxetine | -1.654 | 0.954 | 0.999 | 0.403 |
| 9 day | vehicle vs. 6 mg/kg oxaliplatin | -5.257 | -2.742 | < 0.001 | 4.682 |
|  | vehicle vs. 2 g/kg LEM | -1.257 | 1.257 | 1.000 | 0 |
|  | vehicle vs. 0.5 g/kg LEP-2 | -0.957 | 1.557 | 1.000 | 0.298 |
|  | vehicle vs. 0.25 g/kg LEP-3 | -1.157 | 1.357 | 1.000 | 0.121 |
|  | vehicle vs. 0.25 g/kg LEP-4 | -1.657 | 0.857 | 0.996 | 0.518 |
|  | vehicle vs. 30 mg/kg Duloxetine | -1.657 | 0.857 | 0.996 | 0.573 |
|  | vehicle vs. 6 mg/kg oxaliplatin + 2 g/kg LEM | -1.457 | 1.057 | 1.000 | 0.274 |
|  | vehicle vs. 6 mg/kg oxaliplatin + 0.5 g/kg LEP-2 | -0.757 | 1.757 | 0.973 | 0.553 |
|  | vehicle vs. 6 mg/kg oxaliplatin + 0.25 g/kg LEP-3 | -5.257 | -2.742 | < 0.001 | 4.298 |
|  | vehicle vs. 6 mg/kg oxaliplatin + 0.25 g/kg LEP-4 | -1.257 | 1.257 | 1.000 | 0 |
|  | vehicle vs. 6 mg/kg oxaliplatin + 30 mg/kg Duloxetine | -1.457 | 1.057 | 1.000 | 0.257 |
| 12 day | vehicle vs. 6 mg/kg oxaliplatin | -5.776 | -3.423 | < 0.001 | 5.594 |
|  | vehicle vs. 2 g/kg LEM | -1.176 | 1.176 | 1.000 | 0 |
|  | vehicle vs. 0.5 g/kg LEP-2 | -0.876 | 1.476 | 0.999 | 0.298 |
|  | vehicle vs. 0.25 g/kg LEP-3 | -1.076 | 1.276 | 1.000 | 0.121 |
|  | vehicle vs. 0.25 g/kg LEP-4 | -1.576 | 0.776 | 0.992 | 0.518 |
|  | vehicle vs. 30 mg/kg Duloxetine | -1.576 | 0.776 | 0.992 | 0.573 |
|  | vehicle vs. 6 mg/kg oxaliplatin + 2 g/kg LEM | -1.376 | 0.976 | 1.000 | 0.274 |
|  | vehicle vs. 6 mg/kg oxaliplatin + 0.5 g/kg LEP-2 | -0.676 | 1.676 | 0.957 | 0.553 |
|  | vehicle vs. 6 mg/kg oxaliplatin + 0.25 g/kg LEP-3 | -5.376 | -3.023 | < 0.001 | 5.307 |
|  | vehicle vs. 6 mg/kg oxaliplatin + 0.25 g/kg LEP-4 | -1.176 | 1.176 | 1.000 | 0 |
|  | vehicle vs. 6 mg/kg oxaliplatin + 30 mg/kg Duloxetine | -1.376 | 0.976 | 1.000 | 0.257 |
| 15 day | vehicle vs. 6 mg/kg oxaliplatin | -6.280 | -3.919 | < 0.001 | 6.488 |
|  | vehicle vs. 2 g/kg LEM | -1.180 | 1.180 | 1.000 | 0 |
|  | vehicle vs. 0.5 g/kg LEP-2 | -0.880 | 1.480 | 0.999 | 0.298 |
|  | vehicle vs. 0.25 g/kg LEP-3 | -1.080 | 1.280 | 1.000 | 0.121 |
|  | vehicle vs. 0.25 g/kg LEP-4 | -1.580 | 0.780 | 0.992 | 0.518 |
|  | vehicle vs. 30 mg/kg Duloxetine | -1.580 | 0.780 | 0.992 | 0.573 |
|  | vehicle vs. 6 mg/kg oxaliplatin + 2 g/kg LEM | -1.380 | 0.980 | 1.000 | 0.274 |
|  | vehicle vs. 6 mg/kg oxaliplatin + 0.5 g/kg LEP-2 | -0.680 | 1.680 | 0.958 | 0.553 |
|  | vehicle vs. 6 mg/kg oxaliplatin + 0.25 g/kg LEP-3 | -6.180 | -3.819 | < 0.001 | 5.989 |
|  | vehicle vs. 6 mg/kg oxaliplatin + 0.25 g/kg LEP-4 | -1.180 | 1.180 | 1.000 | 0 |
|  | vehicle vs. 6 mg/kg oxaliplatin + 30 mg/kg Duloxetine | -1.380 | 0.980 | 1.000 | 0.257 |

Supporting Table 15: Effect sizes and 95% confident interval for significant results in Figure 3b.

| Days | Comparison | 95% confidence intervals | | P value | *d* |
| --- | --- | --- | --- | --- | --- |
|  |  | Lower limit | Upper limit |  |  |
| 0 day | vehicle vs. 6 mg/kg oxaliplatin | -0.179 | 1.940 | 0.114 | 0.666 |
|  | vehicle vs. 2 g/kg LEM | -0.910 | 0.850 | 1.000 | 0.034 |
|  | vehicle vs. 0.5 g/kg LEP-2 | -0.640 | 1.120 | 0.999 | 0.420 |
|  | vehicle vs. 0.25 g/kg LEP-3 | -0.789 | 2.550 | 0.684 | 0.916 |
|  | vehicle vs. 0.25 g/kg LEP-4 | -0.680 | 1.080 | 1.000 | 0.338 |
|  | vehicle vs. 30 mg/kg Duloxetine | -0.249 | 2.010 | 0.411 | 0.913 |
|  | vehicle vs. 6 mg/kg oxaliplatin + 2 g/kg LEM | -1.390 | 0.370 | 0.735 | 0.718 |
|  | vehicle vs. 6 mg/kg oxaliplatin + 0.5 g/kg LEP-2 | -0.560 | 1.200 | 0.987 | 0.380 |
|  | vehicle vs. 6 mg/kg oxaliplatin + 0.25 g/kg LEP-3 | -0.880 | 0.880 | 1.000 | 0 |
|  | vehicle vs. 6 mg/kg oxaliplatin + 0.25 g/kg LEP-4 | -0.530 | 1.230 | 0.974 | 0.509 |
|  | vehicle vs. 6 mg/kg oxaliplatin + 30 mg/kg Duloxetine | -0.269 | 1.830 | 0.259 | 0.812 |
| 3 day | vehicle vs. 6 mg/kg oxaliplatin | -0.552 | 1.944 | 0.651 | 0.992 |
|  | vehicle vs. 2 g/kg LEM | -0.504 | 1.384 | 0.921 | 0.527 |
|  | vehicle vs. 0.5 g/kg LEP-2 | -0.604 | 1.284 | 0.988 | 0.497 |
|  | vehicle vs. 0.25 g/kg LEP-3 | -0.395 | 1.684 | 0.441 | 0.770 |
|  | vehicle vs. 0.25 g/kg LEP-4 | -0.584 | 1.304 | 0.981 | 0.533 |
|  | vehicle vs. 30 mg/kg Duloxetine | -0.244 | 1.644 | 0.367 | 0.990 |
|  | vehicle vs. 6 mg/kg oxaliplatin + 2 g/kg LEM | -0.364 | 1.524 | 0.658 | 0.806 |
|  | vehicle vs. 6 mg/kg oxaliplatin + 0.5 g/kg LEP-2 | -0.544 | 1.344 | 0.958 | 0.418 |
|  | vehicle vs. 6 mg/kg oxaliplatin + 0.25 g/kg LEP-3 | -0.352 | 1.124 | 0.334 | 0.876 |
|  | vehicle vs. 6 mg/kg oxaliplatin + 0.25 g/kg LEP-4 | -0.024 | 1.864 | 0.064 | 1.249 |
|  | vehicle vs. 6 mg/kg oxaliplatin + 30 mg/kg Duloxetine | -0.555 | 1.444 | 0.241 | 0.815 |
| 6 day | vehicle vs. 6 mg/kg oxaliplatin | -1.439 | 3.320 | 0.874 | 0.747 |
|  | vehicle vs. 2 g/kg LEM | -0.179 | 1.960 | 0.114 | 1.106 |
|  | vehicle vs. 0.5 g/kg LEP-2 | -0.010 | 1.870 | 0.056 | 1.503 |
|  | vehicle vs. 0.25 g/kg LEP-3 | -0.779 | 2.660 | 0.741 | 1.176 |
|  | vehicle vs. 0.25 g/kg LEP-4 | -0.179 | 2.060 | 0.241 | 1.165 |
|  | vehicle vs. 30 mg/kg Duloxetine | -0.369 | 2.250 | 0.512 | 0.912 |
|  | vehicle vs. 6 mg/kg oxaliplatin + 2 g/kg LEM | -0.389 | 2.270 | 0.498 | 0.934 |
|  | vehicle vs. 6 mg/kg oxaliplatin + 0.5 g/kg LEP-2 | -0.189 | 2.070 | 0.211 | 0.927 |
|  | vehicle vs. 6 mg/kg oxaliplatin + 0.25 g/kg LEP-3 | -1.049 | 2.930 | 0.891 | 0.930 |
|  | vehicle vs. 6 mg/kg oxaliplatin + 0.25 g/kg LEP-4 | -0.899 | 2.780 | 0.912 | 0.561 |
|  | vehicle vs. 6 mg/kg oxaliplatin + 30 mg/kg Duloxetine | -0.649 | 2.530 | 0.697 | 0.838 |
| 9 day | vehicle vs. 6 mg/kg oxaliplatin | -0.972 | 3.187 | 0.641 | 0.523 |
|  | vehicle vs. 2 g/kg LEM | -0.172 | 2.387 | 0.574 | 0.882 |
|  | vehicle vs. 0.5 g/kg LEP-2 | -0.407 | 1.807 | 0.615 | 0.890 |
|  | vehicle vs. 0.25 g/kg LEP-3 | -0.572 | 2.787 | 0.547 | 0.449 |
|  | vehicle vs. 0.25 g/kg LEP-4 | -0.272 | 2.487 | 0.203 | 0.677 |
|  | vehicle vs. 30 mg/kg Duloxetine | -0.107 | 2.107 | 0.118 | 0.841 |
|  | vehicle vs. 6 mg/kg oxaliplatin + 2 g/kg LEM | -0.552 | 2.767 | 0.611 | 0.586 |
|  | vehicle vs. 6 mg/kg oxaliplatin + 0.5 g/kg LEP-2 | -0.027 | 2.187 | 0.063 | 1.342 |
|  | vehicle vs. 6 mg/kg oxaliplatin + 0.25 g/kg LEP-3 | -1.472 | 3.687 | 0.891 | 0.566 |
|  | vehicle vs. 6 mg/kg oxaliplatin + 0.25 g/kg LEP-4 | -1.952 | 4.167 | 0.911 | 0.458 |
|  | vehicle vs. 6 mg/kg oxaliplatin + 30 mg/kg Duloxetine | -0.067 | 2.147 | 0.087 | 1.135 |
| 12 day | vehicle vs. 6 mg/kg oxaliplatin | -0.972 | 3.187 | 0.641 | 0.523 |
|  | vehicle vs. 2 g/kg LEM | -0.172 | 2.387 | 0.574 | 0.882 |
|  | vehicle vs. 0.5 g/kg LEP-2 | -0.407 | 1.807 | 0.615 | 0.890 |
|  | vehicle vs. 0.25 g/kg LEP-3 | -0.572 | 2.787 | 0.547 | 0.449 |
|  | vehicle vs. 0.25 g/kg LEP-4 | -0.272 | 2.487 | 0.203 | 0.677 |
|  | vehicle vs. 30 mg/kg Duloxetine | -0.107 | 2.107 | 0.118 | 0.841 |
|  | vehicle vs. 6 mg/kg oxaliplatin + 2 g/kg LEM | -0.552 | 2.767 | 0.611 | 0.586 |
|  | vehicle vs. 6 mg/kg oxaliplatin + 0.5 g/kg LEP-2 | -0.027 | 2.187 | 0.063 | 1.342 |
|  | vehicle vs. 6 mg/kg oxaliplatin + 0.25 g/kg LEP-3 | -1.472 | 3.687 | 0.891 | 0.566 |
|  | vehicle vs. 6 mg/kg oxaliplatin + 0.25 g/kg LEP-4 | -1.952 | 4.167 | 0.911 | 0.458 |
|  | vehicle vs. 6 mg/kg oxaliplatin + 30 mg/kg Duloxetine | -0.067 | 2.147 | 0.087 | 1.135 |
| 15 day | vehicle vs. 6 mg/kg oxaliplatin | -0.972 | 3.187 | 0.641 | 3.023 |
|  | vehicle vs. 2 g/kg LEM | -0.172 | 2.387 | 0.574 | 1.482 |
|  | vehicle vs. 0.5 g/kg LEP-2 | -0.407 | 1.807 | 0.615 | 0.890 |
|  | vehicle vs. 0.25 g/kg LEP-3 | -0.572 | 2.787 | 0.547 | 2.449 |
|  | vehicle vs. 0.25 g/kg LEP-4 | -0.272 | 2.487 | 0.203 | 1.773 |
|  | vehicle vs. 30 mg/kg Duloxetine | -0.107 | 2.107 | 0.118 | 1.411 |
|  | vehicle vs. 6 mg/kg oxaliplatin + 2 g/kg LEM | -0.552 | 2.767 | 0.611 | 2.086 |
|  | vehicle vs. 6 mg/kg oxaliplatin + 0.5 g/kg LEP-2 | -0.027 | 2.187 | 0.063 | 1.342 |
|  | vehicle vs. 6 mg/kg oxaliplatin + 0.25 g/kg LEP-3 | -1.472 | 3.687 | 0.891 | 2.566 |
|  | vehicle vs. 6 mg/kg oxaliplatin + 0.25 g/kg LEP-4 | -1.952 | 4.167 | 0.911 | 4.958 |
|  | vehicle vs. 6 mg/kg oxaliplatin + 30 mg/kg Duloxetine | -0.067 | 2.147 | 0.087 | 1.135 |

Supporting Table 16: Effect sizes and 95% confident interval for significant results in Figure 4a and 4b.

| mRNA | Comparison | 95% confidence intervals | | P value | *d* |
| --- | --- | --- | --- | --- | --- |
|  |  | Lower limit | Upper limit |  |  |
| Cav3.2 | vehicle vs. 6 mg/kg oxaliplatin | 22.720 | 33.256 | < 0.001 | 16.674 |
|  | vehicle vs. 0.25 g/kg LEP-4 | -4.998 | 5.537 | 0.998 | 0.556 |
|  | vehicle vs. 6 mg/kg oxaliplatin + 0.25 g/kg LEP-4 | -5.232 | 5.304 | 1.000 | 0.056 |
| NR2A | vehicle vs. 6 mg/kg oxaliplatin | 13.112 | 19.078 | < 0.001 | 12.799 |
|  | vehicle vs. 0.25 g/kg LEP-4 | -2.922 | 3.043 | 1.000 | 0.094 |
|  | vehicle vs. 6 mg/kg oxaliplatin + 0.25 g/kg LEP-4 | -2.728 | 3.238 | 0.991 | 0.436 |

Supporting Table 17: Effect sizes and 95% confident interval for significant results in Figure 4d and 4f.

| Sample | Comparison | 95% confidence intervals | | P value | *d* |
| --- | --- | --- | --- | --- | --- |
|  |  | Lower limit | Upper limit |  |  |
| DRG | vehicle vs. 6 mg/kg oxaliplatin | 1.666 | 2.298 | < 0.001 | 4.493 |
|  | vehicle vs. 0.25 g/kg LEP-4 | -0.368 | 0.263 | 0.944 | 0.139 |
|  | vehicle vs. 6 mg/kg oxaliplatin + 0.25 g/kg LEP-4 | -0.329 | 0.302 | 0.999 | 0.033 |
| Spinal cord L4~L6 | vehicle vs. 6 mg/kg oxaliplatin | 1.836 | 2.758 | < 0.001 | 4.811 |
|  | vehicle vs. 0.25 g/kg LEP-4 | -0.515 | 0.406 | 0.978 | 0.129 |
|  | vehicle vs. 6 mg/kg oxaliplatin + 0.25 g/kg LEP-4 | -0.537 | 0.383 | 0.942 | 0.199 |

Supporting Table 18: Effect sizes and 95% confident interval for significant results in Figure 5a.

| Days | Comparison | 95% confidence intervals | | P value | *r* |
| --- | --- | --- | --- | --- | --- |
|  |  | Lower limit | Upper limit |  |  |
| 0 day | vehicle vs. 6 mg/kg oxaliplatin | -34.553 | 18.553 | 0.085 | 0.655 |
|  | vehicle vs. 0.25 g/kg LEP-4 | -34.553 | 18.553 | 0.085 | 0.655 |
|  | vehicle vs. 6 mg/kg oxaliplatin + 0.25 g/kg LEP-4 | -34.553 | 18.553 | 0.085 | 0.655 |
|  | 6 mg/kg oxaliplatin vs. 6 mg/kg oxaliplatin + 0.25 g/kg LEP-4 | -26.553 | 26.553 | 1.000 | 0 |
| 3 day | vehicle vs. 6 mg/kg oxaliplatin | -6.553 | 46.553 | 0.009 | 0.986 |
|  | vehicle vs. 0.25 g/kg LEP-4 | -26.553 | 26.553 | 1.000 | 0 |
|  | vehicle vs. 6 mg/kg oxaliplatin + 0.25 g/kg LEP-4 | -1.553 | 51.553 | < 0.001 | 1.144 |
|  | 6 mg/kg oxaliplatin vs. 6 mg/kg oxaliplatin + 0.25 g/kg LEP-4 | -16.553 | 36.553 | 0.241 | 0.457 |
| 6 day | vehicle vs. 6 mg/kg oxaliplatin | -8.953 | 44.153 | 0.006 | 0.785 |
|  | vehicle vs. 0.25 g/kg LEP-4 | -29.553 | 23.553 | 0.949 | 0.133 |
|  | vehicle vs. 6 mg/kg oxaliplatin + 0.25 g/kg LEP-4 | -7.1537 | 45.953 | 0.002 | 0.865 |
|  | 6 mg/kg oxaliplatin vs. 6 mg/kg oxaliplatin + 0.25 g/kg LEP-4 | -24.753 | 28.353 | 0.988 | 0.080 |
| 9 day | vehicle vs. 6 mg/kg oxaliplatin | -1.553 | 51.553 | < 0.001 | 1.144 |
|  | vehicle vs. 0.25 g/kg LEP-4 | -26.553 | 26.553 | 1.000 | 0 |
|  | vehicle vs. 6 mg/kg oxaliplatin + 0.25 g/kg LEP-4 | -1.553 | 51.553 | < 0.001 | 1.144 |
|  | 6 mg/kg oxaliplatin vs. 6 mg/kg oxaliplatin + 0.25 g/kg LEP-4 | -8.953 | 44.153 | 0.006 | 0.785 |
| 12 day | vehicle vs. 6 mg/kg oxaliplatin | -5.053 | 48.053 | < 0.001 | 1.106 |
|  | vehicle vs. 0.25 g/kg LEP-4 | -26.553 | 26.553 | 1.000 | 0 |
|  | vehicle vs. 6 mg/kg oxaliplatin + 0.25 g/kg LEP-4 | -22.0537 | 31.053 | 0.784 | 0.231 |
|  | 6 mg/kg oxaliplatin vs. 6 mg/kg oxaliplatin + 0.25 g/kg LEP-4 | -43.553 | 9.553 | 0.002 | 0.874 |
| 15 day | vehicle vs. 6 mg/kg oxaliplatin | -2.553 | 50.553 | < 0.001 | 1.125 |
|  | vehicle vs. 0.25 g/kg LEP-4 | -21.903 | 31.203 | 0.813 | 0.218 |
|  | vehicle vs. 6 mg/kg oxaliplatin + 0.25 g/kg LEP-4 | -19.203 | 33.903 | 0.498 | 0.344 |
|  | 6 mg/kg oxaliplatin vs. 6 mg/kg oxaliplatin + 0.25 g/kg LEP-4 | -43.203 | 9.903 | 0.007 | 0.781 |
| 18 day | vehicle vs. 6 mg/kg oxaliplatin | -5.553 | 47.553 | < 0.001 | 1.109 |
|  | vehicle vs. 0.25 g/kg LEP-4 | -26.553 | 26.553 | 1.000 | 0 |
|  | vehicle vs. 6 mg/kg oxaliplatin + 0.25 g/kg LEP-4 | -23.553 | 29.553 | 0.918 | 0.158 |
|  | 6 mg/kg oxaliplatin vs. 6 mg/kg oxaliplatin + 0.25 g/kg LEP-4 | -44.553 | 8.553 | < 0.001 | 0.950 |
| 21 day | vehicle vs. 6 mg/kg oxaliplatin | -4.053 | 49.053 | < 0.001 | 1.108 |
|  | vehicle vs. 0.25 g/kg LEP-4 | -26.553 | 26.553 | 1.000 | 0 |
|  | vehicle vs. 6 mg/kg oxaliplatin + 0.25 g/kg LEP-4 | -19.053 | 34.053 | 0.435 | 0.369 |
|  | 6 mg/kg oxaliplatin vs. 6 mg/kg oxaliplatin + 0.25 g/kg LEP-4 | -36.553 | 16.553 | 0.009 | 0.738 |
| 24 day | vehicle vs. 6 mg/kg oxaliplatin | -8.253 | 44.853 | 0.001 | 0.901 |
|  | vehicle vs. 0.25 g/kg LEP-4 | -30.753 | 22.353 | 0.836 | 0.206 |
|  | vehicle vs. 6 mg/kg oxaliplatin + 0.25 g/kg LEP-4 | -27.453 | 25.653 | 0.998 | 0.044 |
|  | 6 mg/kg oxaliplatin vs. 6 mg/kg oxaliplatin + 0.25 g/kg LEP-4 | -45.753 | 7.353 | < 0.001 | 0.945 |

Supporting Table 19: Effect sizes and 95% confident interval for significant results in Figure 5c.

| Days | Comparison | 95% confidence intervals | | P value | *d* |
| --- | --- | --- | --- | --- | --- |
|  |  | Lower limit | Upper limit |  |  |
| 0 day | vehicle vs. 6 mg/kg oxaliplatin | -0.397 | 0.697 | 0.881 | 0.431 |
|  | vehicle vs. 0.25 g/kg LEP-4 | -0.247 | 0.847 | 0.463 | 0.610 |
|  | vehicle vs. 6 mg/kg oxaliplatin + 0.25 g/kg LEP-4 | -0.997 | 0.097 | 0.139 | 0.611 |
|  | 6 mg/kg oxaliplatin vs. 6 mg/kg oxaliplatin + 0.25 g/kg LEP-4 | -1.147 | 0.052 | 0.071 | 0.924 |
| 3 day | vehicle vs. 6 mg/kg oxaliplatin | -2.205 | -0.494 | 0.001 | 3.884 |
|  | vehicle vs. 0.25 g/kg LEP-4 | -1.155 | 0.555 | 0.781 | 0.379 |
|  | vehicle vs. 6 mg/kg oxaliplatin + 0.25 g/kg LEP-4 | -2.705 | -0.994 | < 0.001 | 3.307 |
|  | 6 mg/kg oxaliplatin vs. 6 mg/kg oxaliplatin + 0.25 g/kg LEP-4 | -1.355 | 0.355 | 0.405 | 0.759 |
| 6 day | vehicle vs. 6 mg/kg oxaliplatin | -4.888 | -3.011 | < 0.001 | 5.248 |
|  | vehicle vs. 0.25 g/kg LEP-4 | -1.188 | 0.688 | 0.889 | 0.281 |
|  | vehicle vs. 6 mg/kg oxaliplatin + 0.25 g/kg LEP-4 | -5.238 | -3.361 | < 0.001 | 4.628 |
|  | 6 mg/kg oxaliplatin vs. 6 mg/kg oxaliplatin + 0.25 g/kg LEP-4 | -1.288 | 0.588 | 0.748 | 0.416 |
| 9 day | vehicle vs. 6 mg/kg oxaliplatin | -5.254 | -3.345 | < 0.001 | 6.441 |
|  | vehicle vs. 0.25 g/kg LEP-4 | -0.854 | 1.054 | 0.992 | 0.105 |
|  | vehicle vs. 6 mg/kg oxaliplatin + 0.25 g/kg LEP-4 | -4.054 | -2.145 | < 0.001 | 4.238 |
|  | 6 mg/kg oxaliplatin vs. 6 mg/kg oxaliplatin + 0.25 g/kg LEP-4 | 0.245 | 2.154 | 0.009 | 1.701 |
| 12 day | vehicle vs. 6 mg/kg oxaliplatin | -5.801 | -3.590 | < 0.001 | 5.174 |
|  | vehicle vs. 0.25 g/kg LEP-4 | -1.259 | 0.959 | 0.983 | 0.145 |
|  | vehicle vs. 6 mg/kg oxaliplatin + 0.25 g/kg LEP-4 | -3.709 | -1.490 | < 0.001 | 3.380 |
|  | 6 mg/kg oxaliplatin vs. 6 mg/kg oxaliplatin + 0.25 g/kg LEP-4 | 0.990 | 3.209 | < 0.001 | 2.621 |
| 15 day | vehicle vs. 6 mg/kg oxaliplatin | -5.948 | -3.751 | < 0.001 | 5.540 |
|  | vehicle vs. 0.25 g/kg LEP-4 | -1.548 | 0.648 | 0.690 | 0.579 |
|  | vehicle vs. 6 mg/kg oxaliplatin + 0.25 g/kg LEP-4 | -3.448 | -1.251 | < 0.001 | 3.230 |
|  | 6 mg/kg oxaliplatin vs. 6 mg/kg oxaliplatin + 0.25 g/kg LEP-4 | 1.401 | 3.598 | < 0.001 | 2.435 |
| 18 day | vehicle vs. 6 mg/kg oxaliplatin | -5.499 | -3.600 | < 0.001 | 6.995 |
|  | vehicle vs. 0.25 g/kg LEP-4 | -1.199 | 0.699 | 0.893 | 0.278 |
|  | vehicle vs. 6 mg/kg oxaliplatin + 0.25 g/kg LEP-4 | -2.599 | -0.700 | < 0.001 | 2.107 |
|  | 6 mg/kg oxaliplatin vs. 6 mg/kg oxaliplatin + 0.25 g/kg LEP-4 | 1.950 | 3.849 | < 0.001 | 3.659 |
| 21 day | vehicle vs. 6 mg/kg oxaliplatin | -5.628 | -4.271 | < 0.001 | 7.379 |
|  | vehicle vs. 0.25 g/kg LEP-4 | -0.828 | 0.528 | 0.933 | 0.202 |
|  | vehicle vs. 6 mg/kg oxaliplatin + 0.25 g/kg LEP-4 | -2.628 | -1.271 | < 0.001 | 4.100 |
|  | 6 mg/kg oxaliplatin vs. 6 mg/kg oxaliplatin + 0.25 g/kg LEP-4 | 2.321 | 3.678 | < 0.001 | 4.550 |
| 24 day | vehicle vs. 6 mg/kg oxaliplatin | -6.254 | -4.645 | < 0.001 | 7.890 |
|  | vehicle vs. 0.25 g/kg LEP-4 | -1.654 | 0.452 | 0.498 | 0.790 |
|  | vehicle vs. 6 mg/kg oxaliplatin + 0.25 g/kg LEP-4 | -2.654 | -1.045 | < 0.001 | 2.201 |
|  | 6 mg/kg oxaliplatin vs. 6 mg/kg oxaliplatin + 0.25 g/kg LEP-4 | 2.795 | 4.404 | < 0.001 | 4.40 |

Supporting Table 20: Effect sizes and 95% confident interval for significant results in Figure 5d.

| Days | Comparison | 95% confidence intervals | | P value | *d* |
| --- | --- | --- | --- | --- | --- |
|  |  | Lower limit | Upper limit |  |  |
| 0 day | vehicle vs. 6 mg/kg oxaliplatin | -0.696 | 0.996 | 0.964 | 0.213 |
|  | vehicle vs. 0.25 g/kg LEP-4 | -1.096 | 0.596 | 0.856 | 0.423 |
|  | vehicle vs. 6 mg/kg oxaliplatin + 0.25 g/kg LEP-4 | -1.096 | 0.596 | 0.856 | 0.304 |
|  | 6 mg/kg oxaliplatin vs. 6 mg/kg oxaliplatin + 0.25 g/kg LEP-4 | -1.246 | 0.446 | 0.586 | 0.419 |
| 3 day | vehicle vs. 6 mg/kg oxaliplatin | -2.745 | -1.254 | < 0.001 | 3.935 |
|  | vehicle vs. 0.25 g/kg LEP-4 | -0.845 | 0.645 | 0.984 | 0.133 |
|  | vehicle vs. 6 mg/kg oxaliplatin + 0.25 g/kg LEP-4 | -3.045 | -1.554 | < 0.001 | 3.137 |
|  | 6 mg/kg oxaliplatin vs. 6 mg/kg oxaliplatin + 0.25 g/kg LEP-4 | -1.045 | 0.445 | 0.702 | 0.409 |
| 6 day | vehicle vs. 6 mg/kg oxaliplatin | -5.852 | -2.947 | < 0.001 | 4.309 |
|  | vehicle vs. 0.25 g/kg LEP-4 | -1.002 | 1.902 | 0.838 | 0.372 |
|  | vehicle vs. 6 mg/kg oxaliplatin + 0.25 g/kg LEP-4 | -5.902 | -2.997 | < 0.001 | 3.802 |
|  | 6 mg/kg oxaliplatin vs. 6 mg/kg oxaliplatin + 0.25 g/kg LEP-4 | -1.502 | 1.402 | 1.000 | 0.055 |
| 9 day | vehicle vs. 6 mg/kg oxaliplatin | -5.221 | -3.278 | < 0.001 | 6.263 |
|  | vehicle vs. 0.25 g/kg LEP-4 | -0.171 | 1.771 | 0.138 | 0.834 |
|  | vehicle vs. 6 mg/kg oxaliplatin + 0.25 g/kg LEP-4 | -3.471 | -1.528 | < 0.001 | 3.418 |
|  | 6 mg/kg oxaliplatin vs. 6 mg/kg oxaliplatin + 0.25 g/kg LEP-4 | 0.778 | 2.721 | < 0.001 | 2.444 |
| 12 day | vehicle vs. 6 mg/kg oxaliplatin | -5.176 | -2.623 | < 0.001 | 4.046 |
|  | vehicle vs. 0.25 g/kg LEP-4 | -0.826 | 1.726 | 0.778 | 0.374 |
|  | vehicle vs. 6 mg/kg oxaliplatin + 0.25 g/kg LEP-4 | -3.176 | -0.623 | 0.002 | 1.971 |
|  | 6 mg/kg oxaliplatin vs. 6 mg/kg oxaliplatin + 0.25 g/kg LEP-4 | 0.723 | 3.276 | 0.001 | 3.130 |
| 15 day | vehicle vs. 6 mg/kg oxaliplatin | -5.560 | -4.039 | < 0.001 | 5.793 |
|  | vehicle vs. 0.25 g/kg LEP-4 | -0.410 | 1.110 | 0.606 | 0.393 |
|  | vehicle vs. 6 mg/kg oxaliplatin + 0.25 g/kg LEP-4 | -2.710 | -1.189 | < 0.001 | 2.605 |
|  | 6 mg/kg oxaliplatin vs. 6 mg/kg oxaliplatin + 0.25 g/kg LEP-4 | 2.089 | 3.610 | < 0.001 | 4.563 |
| 18 day | vehicle vs. 6 mg/kg oxaliplatin | -5.807 | -3.992 | < 0.001 | 7.206 |
|  | vehicle vs. 0.25 g/kg LEP-4 | -0.407 | 1.407 | 0.457 | 0.520 |
|  | vehicle vs. 6 mg/kg oxaliplatin + 0.25 g/kg LEP-4 | -2.557 | -0.742 | < 0.001 | 2.642 |
|  | 6 mg/kg oxaliplatin vs. 6 mg/kg oxaliplatin + 0.25 g/kg LEP-4 | 2.342 | 4.157 | < 0.001 | 5.607 |
| 21 day | vehicle vs. 6 mg/kg oxaliplatin | -5.371 | -3.728 | < 0.001 | 6.843 |
|  | vehicle vs. 0.25 g/kg LEP-4 | -0.271 | 1.371 | 0.289 | 0.616 |
|  | vehicle vs. 6 mg/kg oxaliplatin + 0.25 g/kg LEP-4 | -2.221 | -0.578 | < 0.001 | 1.729 |
|  | 6 mg/kg oxaliplatin vs. 6 mg/kg oxaliplatin + 0.25 g/kg LEP-4 | 2.328 | 3.971 | < 0.001 | 4.586 |
| 24 day | vehicle vs. 6 mg/kg oxaliplatin | -5.642 | -3.457 | < 0.001 | 6.843 |
|  | vehicle vs. 0.25 g/kg LEP-4 | -0.842 | 1.342 | 0.926 | 0.267 |
|  | vehicle vs. 6 mg/kg oxaliplatin + 0.25 g/kg LEP-4 | -2.642 | -0.457 | < 0.001 | 1.599 |
|  | 6 mg/kg oxaliplatin vs. 6 mg/kg oxaliplatin + 0.25 g/kg LEP-4 | 1.907 | 4.092 | < 0.001 | 3.452 |

Supporting Table 21: Effect sizes and 95% confident interval for significant results in Figure 5e.

| Days | Comparison | 95% confidence intervals | | P value | *d* |
| --- | --- | --- | --- | --- | --- |
|  |  | Lower limit | Upper limit |  |  |
| 0 day | vehicle vs. 6 mg/kg oxaliplatin | -0.962 | 0.362 | 0.619 | 0.519 |
|  | vehicle vs. 0.25 g/kg LEP-4 | -0.762 | 0.562 | 0.977 | 0.136 |
|  | vehicle vs. 6 mg/kg oxaliplatin + 0.25 g/kg LEP-4 | -1.062 | 0.262 | 0.378 | 0.528 |
|  | 6 mg/kg oxaliplatin vs. 6 mg/kg oxaliplatin + 0.25 g/kg LEP-4 | -0.762 | 0.562 | 0.977 | 0.141 |
| 3 day | vehicle vs. 6 mg/kg oxaliplatin | -3.407 | -1.692 | < 0.001 | 3.219 |
|  | vehicle vs. 0.25 g/kg LEP-4 | -0.757 | 0.957 | 0.989 | 0.108 |
|  | vehicle vs. 6 mg/kg oxaliplatin + 0.25 g/kg LEP-4 | -3.607 | -1.892 | < 0.001 | 3.228 |
|  | 6 mg/kg oxaliplatin vs. 6 mg/kg oxaliplatin + 0.25 g/kg LEP-4 | -1.057 | 0.657 | 0.922 | 0.273 |
| 6 day | vehicle vs. 6 mg/kg oxaliplatin | -4.567 | -2.232 | < 0.001 | 3.906 |
|  | vehicle vs. 0.25 g/kg LEP-4 | -1.017 | 1.317 | 0.986 | 0.138 |
|  | vehicle vs. 6 mg/kg oxaliplatin + 0.25 g/kg LEP-4 | -4.717 | -2.382 | < 0.001 | 4.111 |
|  | 6 mg/kg oxaliplatin vs. 6 mg/kg oxaliplatin + 0.25 g/kg LEP-4 | -1.317 | 1.017 | 0.986 | 0.186 |
| 9 day | vehicle vs. 6 mg/kg oxaliplatin | -4.361 | -3.439 | < 0.001 | 6.691 |
|  | vehicle vs. 0.25 g/kg LEP-4 | -0.311 | 0.611 | 0.817 | 0.263 |
|  | vehicle vs. 6 mg/kg oxaliplatin + 0.25 g/kg LEP-4 | -2.161 | -1.239 | < 0.001 | 2.974 |
|  | 6 mg/kg oxaliplatin vs. 6 mg/kg oxaliplatin + 0.25 g/kg LEP-4 | 1.739 | 2.661 | < 0.001 | 3.375 |
| 12 day | vehicle vs. 6 mg/kg oxaliplatin | -4.949 | -2.450 | < 0.001 | 3.457 |
|  | vehicle vs. 0.25 g/kg LEP-4 | -1.449 | 1.049 | 0.973 | 0.173 |
|  | vehicle vs. 6 mg/kg oxaliplatin + 0.25 g/kg LEP-4 | -2.899 | -0.400 | 0.006 | 1.621 |
|  | 6 mg/kg oxaliplatin vs. 6 mg/kg oxaliplatin + 0.25 g/kg LEP-4 | 0.800 | 3.299 | < 0.001 | 3.355 |
| 15 day | vehicle vs. 6 mg/kg oxaliplatin | -4.812 | -3.187 | < 0.001 | 6.074 |
|  | vehicle vs. 0.25 g/kg LEP-4 | -0.712 | 0.912 | 0.987 | 0.143 |
|  | vehicle vs. 6 mg/kg oxaliplatin + 0.25 g/kg LEP-4 | -1.962 | -0.337 | 0.003 | 1.509 |
|  | 6 mg/kg oxaliplatin vs. 6 mg/kg oxaliplatin + 0.25 g/kg LEP-4 | 2.037 | 3.662 | < 0.001 | 3.242 |
| 18 day | vehicle vs. 6 mg/kg oxaliplatin | -4.906 | -3.093 | < 0.001 | 6.260 |
|  | vehicle vs. 0.25 g/kg LEP-4 | -0.906 | 0.906 | 1.000 | 0 |
|  | vehicle vs. 6 mg/kg oxaliplatin + 0.25 g/kg LEP-4 | -1.306 | 0.506 | 0.638 | 0.429 |
|  | 6 mg/kg oxaliplatin vs. 6 mg/kg oxaliplatin + 0.25 g/kg LEP-4 | 2.693 | 4.506 | < 0.001 | 3.862 |
| 21 day | vehicle vs. 6 mg/kg oxaliplatin | -4.719 | -2.980 | < 0.001 | 6.754 |
|  | vehicle vs. 0.25 g/kg LEP-4 | -0.819 | 0.919 | 0.999 | 0.066 |
|  | vehicle vs. 6 mg/kg oxaliplatin + 0.25 g/kg LEP-4 | -1.519 | 0.219 | 0.202 | 0.735 |
|  | 6 mg/kg oxaliplatin vs. 6 mg/kg oxaliplatin + 0.25 g/kg LEP-4 | 2.330 | 4.069 | < 0.001 | 3.828 |
| 24 day | vehicle vs. 6 mg/kg oxaliplatin | -4.677 | -3.022 | < 0.001 | 4.542 |
|  | vehicle vs. 0.25 g/kg LEP-4 | -0.627 | 1.027 | 0.914 | 0.213 |
|  | vehicle vs. 6 mg/kg oxaliplatin + 0.25 g/kg LEP-4 | -1.627 | 0.027 | 0.061 | 0.956 |
|  | 6 mg/kg oxaliplatin vs. 6 mg/kg oxaliplatin + 0.25 g/kg LEP-4 | 2.222 | 3.877 | < 0.001 | 4.892 |

Supporting Table 22: Effect sizes and 95% confident interval for significant results in Figure 5b.

| Days | Comparison | 95% confidence intervals | | P value | *d* |
| --- | --- | --- | --- | --- | --- |
|  |  | Lower limit | Upper limit |  |  |
| 0 day | vehicle vs. 6 mg/kg oxaliplatin | -1.101 | 0.441 | 0.661 | 0.389 |
|  | vehicle vs. 0.25 g/kg LEP-4 | -1.291 | 0.251 | 0.283 | 0.705 |
|  | vehicle vs. 6 mg/kg oxaliplatin + 0.25 g/kg LEP-4 | -1.511 | 0.268 | 0.215 | 0.804 |
|  | 6 mg/kg oxaliplatin vs. 6 mg/kg oxaliplatin + 0.25 g/kg LEP-4 | -1.181 | 0.438 | 0.631 | 0.429 |
| 3 day | vehicle vs. 6 mg/kg oxaliplatin | -0.790 | 0.870 | 0.999 | 0.048 |
|  | vehicle vs. 0.25 g/kg LEP-4 | -1.320 | 0.340 | 0.397 | 0.641 |
|  | vehicle vs. 6 mg/kg oxaliplatin + 0.25 g/kg LEP-4 | -1.410 | 0.449 | 0.541 | 0.769 |
|  | 6 mg/kg oxaliplatin vs. 6 mg/kg oxaliplatin + 0.25 g/kg LEP-4 | -1.450 | 0.489 | 0.501 | 0.862 |
| 6 day | vehicle vs. 6 mg/kg oxaliplatin | -0.801 | 0.581 | 0.973 | 0.135 |
|  | vehicle vs. 0.25 g/kg LEP-4 | -1.341 | 0.041 | 0.072 | 0.842 |
|  | vehicle vs. 6 mg/kg oxaliplatin + 0.25 g/kg LEP-4 | -1.811 | 0.428 | 0.261 | 0.484 |
|  | 6 mg/kg oxaliplatin vs. 6 mg/kg oxaliplatin + 0.25 g/kg LEP-4 | -1.701 | 0.318 | 0.212 | 0.382 |
| 9 day | vehicle vs. 6 mg/kg oxaliplatin | -0.938 | 0.538 | 0.885 | 0.252 |
|  | vehicle vs. 0.25 g/kg LEP-4 | -0.808 | 0.668 | 0.994 | 0.094 |
|  | vehicle vs. 6 mg/kg oxaliplatin + 0.25 g/kg LEP-4 | -1.518 | 0.041 | 0.085 | 0.951 |
|  | 6 mg/kg oxaliplatin vs. 6 mg/kg oxaliplatin + 0.25 g/kg LEP-4 | -1.318 | 0.158 | 0.168 | 0.725 |
| 12 day | vehicle vs. 6 mg/kg oxaliplatin | -1.135 | 0.595 | 0.835 | 0.308 |
|  | vehicle vs. 0.25 g/kg LEP-4 | -1.045 | 0.685 | 0.943 | 0.223 |
|  | vehicle vs. 6 mg/kg oxaliplatin + 0.25 g/kg LEP-4 | -1.075 | 0.655 | 0.914 | 0.247 |
|  | 6 mg/kg oxaliplatin vs. 6 mg/kg oxaliplatin + 0.25 g/kg LEP-4 | -0.805 | 0.925 | 0.998 | 0.067 |
| 15 day | vehicle vs. 6 mg/kg oxaliplatin | -0.510 | 1.370 | 0.611 | 0.483 |
|  | vehicle vs. 0.25 g/kg LEP-4 | -1.080 | 0.800 | 0.978 | 0.169 |
|  | vehicle vs. 6 mg/kg oxaliplatin + 0.25 g/kg LEP-4 | -0.850 | 1.030 | 0.994 | 0.112 |
|  | 6 mg/kg oxaliplatin vs. 6 mg/kg oxaliplatin + 0.25 g/kg LEP-4 | -1.280 | 0.600 | 0.765 | 0.368 |
| 18 day | vehicle vs. 6 mg/kg oxaliplatin | -0.880 | 0.920 | 1.000 | 0.023 |
|  | vehicle vs. 0.25 g/kg LEP-4 | -1.440 | 0.360 | 0.384 | 0.723 |
|  | vehicle vs. 6 mg/kg oxaliplatin + 0.25 g/kg LEP-4 | -1.410 | 0.390 | 0.434 | 0.683 |
|  | 6 mg/kg oxaliplatin vs. 6 mg/kg oxaliplatin + 0.25 g/kg LEP-4 | -1.430 | 0.370 | 0.400 | 0.567 |
| 21 day | vehicle vs. 6 mg/kg oxaliplatin | -1.002 | 1.002 | 1.000 | 0 |
|  | vehicle vs. 0.25 g/kg LEP-4 | -1.572 | 0.432 | 0.430 | 0.809 |
|  | vehicle vs. 6 mg/kg oxaliplatin + 0.25 g/kg LEP-4 | -1.632 | 0.372 | 0.342 | 0.693 |
|  | 6 mg/kg oxaliplatin vs. 6 mg/kg oxaliplatin + 0.25 g/kg LEP-4 | -1.632 | 0.372 | 0.342 | 0.610 |
| 24 day | vehicle vs. 6 mg/kg oxaliplatin | -1.249 | 0.589 | 0.769 | 0.360 |
|  | vehicle vs. 0.25 g/kg LEP-4 | -1.349 | 0.489 | 0.594 | 0.597 |
|  | vehicle vs. 6 mg/kg oxaliplatin + 0.25 g/kg LEP-4 | -1.689 | 0.149 | 0.128 | 0.977 |
|  | 6 mg/kg oxaliplatin vs. 6 mg/kg oxaliplatin + 0.25 g/kg LEP-4 | -1.359 | 0.479 | 0.576 | 0.458 |
